# Design, synthesis, and *in vitro* and *in silico* study of 1-benzyl-indole hybrid thiosemicarbazones as competitive tyrosinase inhibitors†

**DOI:** 10.1039/d4ra05015k

**Published:** 2024-09-06

**Authors:** Zahra Batool, Saeed Ullah, Ajmal Khan, Farhan Siddique, Sumaira Nadeem, Abdulrahman Alshammari, Norah A. Albekairi, Rimsha Talib, Ahmed Al-Harrasi, Zahid Shafiq

**Affiliations:** a Institute of Chemical Sciences, Bahauddin Zakariya University Multan-60800 Pakistan zahidshafiq@bzu.edu.pk; b Natural and Medical Sciences Research Centre, University of Nizwa P.O. Box 33, PC 616, Birkat Al Mauz Nizwa Sultanate of Oman aharrasi@unizwa.edu.om; c Department of Pharmaceutical Chemistry, Faculty of Pharmacy, Bahauddin Zakariya University Multan-60800 Pakistan; d Department of Pharmacy, The Women University Multan 60000 Pakistan; e Department of Pharmacology and Toxicology, College of Pharmacy, King Saud University Post bezBox 2455 Riyadh 11451 Saudi Arabia; f Department of Chemical and Biological Engineering, College of Engineering, Korea University 145 Anam-ro, Seongbuk-gu Seoul 02841 Republic of Korea

## Abstract

Developing new anti-tyrosinase drugs seems crucial for the medical and industrial fields since irregular melanin synthesis is linked to the resurgence of several skin conditions, including melanoma, and the browning of fruits and vegetables. A novel series of N-1 and C-3 substituted indole-based thiosemicarbazones 5(a–r) are synthesized and further analyzed for their inhibition potential against tyrosinase enzyme through *in vitro* assays. The synthesized compounds displayed very good to moderate inhibition with half maximal inhibitory concentration in the range of 12.40 ± 0.26 μM to 47.24 ± 1.27 μM. Among all the derivatives 5k displayed the highest inhibitory activity. According to SAR analysis, the derivatives with 4-substitution at the benzyl or phenyl ring of the thiosemicarbazones exhibited better inhibitory potential against tyrosinase. *In silico* analysis (including ADMET prediction and molecular docking) was conducted and compared with the standard drug (kojic acid). These findings may help the hunt for new melanogenesis inhibitors that the food and cosmetics industries may find valuable.

## Abstract 2

Developing new anti-tyrosinase drugs seems crucial for the medical and industrial fields since irregular melanin synthesis is linked to the resurgence of several skin conditions, including melanoma, and the browning of fruits and vegetables.

## Graphical abstract

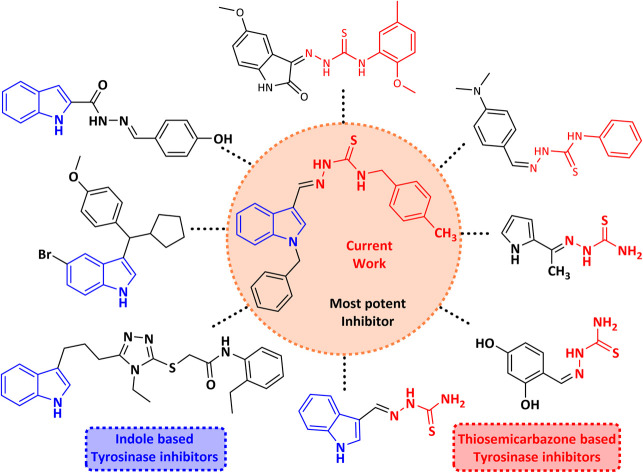

## 1. Introduction

Melanocytes create melanin; tyrosine is a significant rate-limiting enzyme for the synthesis of melanin (EC 1.14.18.1) which has a vital function in shielding the skin from UV ray damage.^1,2^ Melanin is a vital biopolymer controlling skin, eye, and hair color.^3–5^ Tyrosinase is a metalloenzyme that has two bifunctional copper ions in its catalytic site.^6,7^ It controls the key steps in the melanin biosynthesis pathway.^8,9^ Excessive UV exposure results in aberrant melanin synthesis and deposition, often leading to skin pigmentation as in lentigo, freckles, melisma, and other skin conditions.^10,11^ It catalytically oxidizes l-tyrosine to dopaquinone, which regulates the first two steps of melanin biosynthesis:^12^ firstly, it converts l-tyrosine to 3,4-dihydroxyphenylalanine (l-DOPA); secondly, it further oxidizes l-DOPA to dopaquinone, which is a crucial intermediate in the biosynthesis of melanin and is subsequently converted to melanin pigments through related chemical reactions.^13–15^ Thus, tyrosinase inhibition is needed to reduce melanin production.^16,17^ Other than their use in pigmentation disorders, they are also crucial in the food sector. Enzymatic browning is also caused by tyrosinase's ability to catalyze the conversion of phenolic compounds found in fruits and vegetables into quinones. For this reason, regulation of tyrosinase activity is essential for maintaining the quality of fruits and vegetables.^18–20^ Numerous tyrosinase inhibitors, both natural and synthetic, with a broad range of structural variations have been found.^21–26^ However, only a few tyrosinase inhibitors, such as arbutin, glabridin, paeonol, and kojic acid, are employed as effective anti-tyrosinase agents in the food, cosmetics, and pharmaceutical sectors. However, they still have shortcomings, such as limited stability, skin irritation, and poor effectiveness. Thus, the development of safer and more potent tyrosinase inhibitors is necessary.

The indole scaffold, which has a pyrrole structure parallel to benzene, is a principal structural nuclei in drug discovery because of its unique ability to mimic peptide structure and interact with enzymes.^27–31^ Additionally, it has been reported by previous researchers that the activity of indole analogs with N substitution, such as phenyl and benzyl substitution, has enhanced markedly.^32,33^ Recently, a series of novel 1-substituted indole-3-carboxaldehyde thiosemicarbazones were synthesized and investigated as potential anticancer and antimycobacterial agents.^34^ Another study reported two series of *N*-substituted indole derivatives *i.e. N*-substituted indole-based chalcones and *N*-substituted indole-based hydrazide–hydrazones which proved to be potential antimicrobial and antileishmanial agents.^35^*N*-substituted indole derivatives showed increased anticancer activity against numerous cancer cell lines in comparison to the unsubstituted indole derivatives.^36,37^ Certain indole compounds have also been identified as prospective tyrosinase inhibitors in previous investigations.^27,29–31^ Furthermore, indole and its analogs are found in abundance in nature and exhibit high levels of safety. According to the aforementioned studies, indole can act as a fundamental framework for the development of novel tyrosinase inhibitors.

**Fig. 1:**
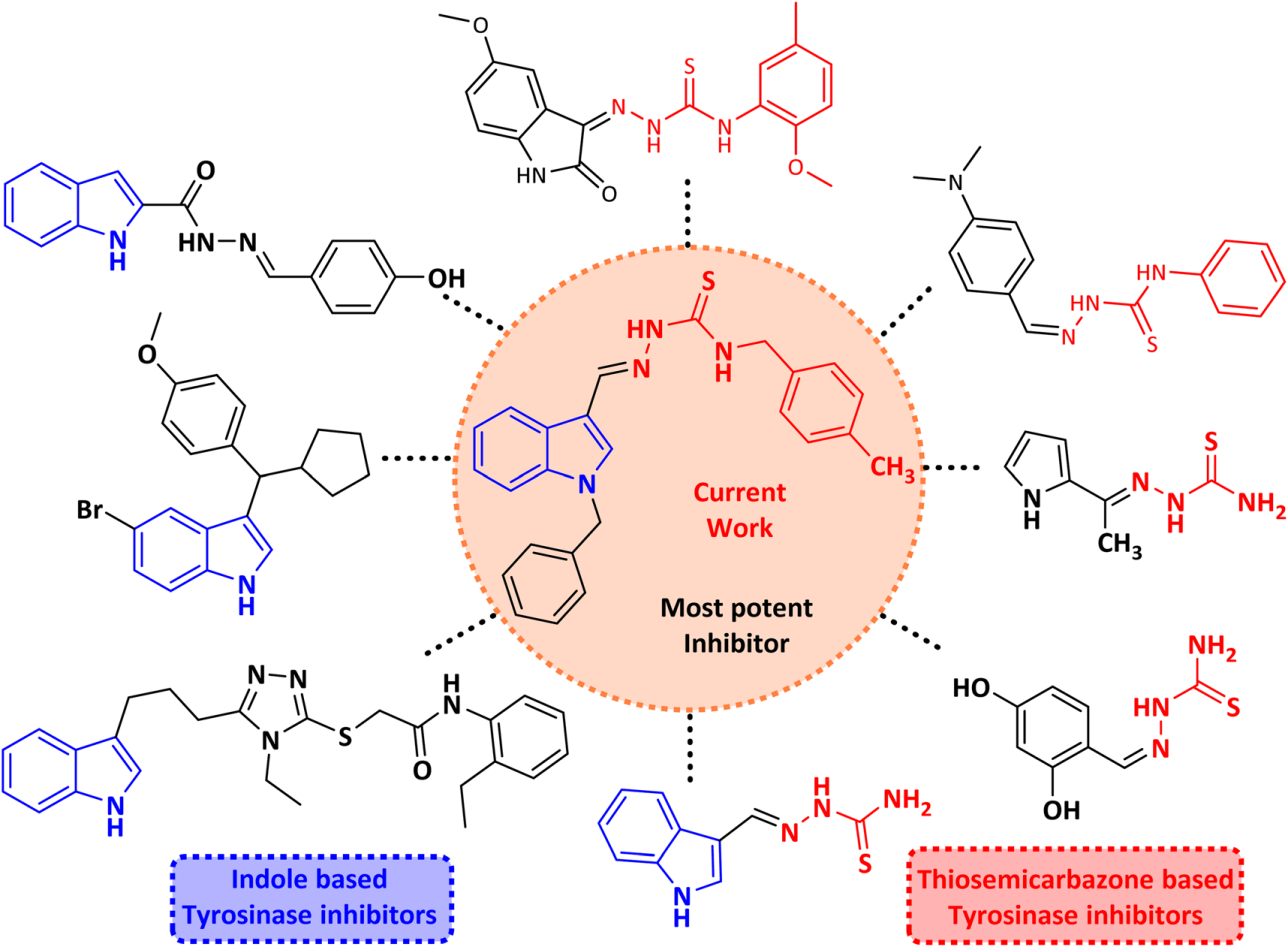
Reported indole and thiosemicarbazone as tyrosinase inhibitors.

Thiosemicarbazones are a fascinating class of compounds. Numerous pharmacological and biological features have been discovered including their efficacy as antibacterial,^38^ antimalarial,^39^ and anticancer^40^ agents, *etc.* On the other hand, thiosemicarbazones also belong to one of the main families of tyrosinase inhibitors because of their typical structural component and capacity for the chelation of copper ions in the tyrosinase enzyme's active site. The structure–activity relationship studies demonstrated that thiosemicarbazide moiety was a crucial component for the determination of the inhibitory action of tyrosinase since the thiosemicarbazide scaffold has the potential to combine the copper ions at the tyrosinase active site successfully.^41^ A literature study has suggested that heterocyclic TSCs were more potent than their aromatic derivatives.^42^ Multiple researchers have recently reported that an extensive range of thiosemicarbazones are effective tyrosinase inhibitors (Fig. 1).^43–51^ In recent work, Masuri *et al.* evaluated the tyrosinase inhibition ability of two new bishydroxylated and two new monohydroxylated derivatives of (1*E*)-2-(1-(2-oxo-2*H*-chromen-3-yl)ethylidene)hydrazine-1-carbothioamide. Interestingly, compared to the reference substance, kojic acid, these compounds exhibit stronger tyrosinase–inhibitory activities.^52^ In another study, several thioquinolines conjugated to thiosemicarbazide and aryl-substituted were synthesized. The most potent derivative bearing 4-chlorophenyl ring demonstrated an IC_50_ value of 25.75 ± 0.19 μM compared to that of kojic acid as the positive control (IC_50_ = 34.93 ± 0.06 μM).^53^ In another work, methyl 4-pyridyl ketone thiosemicarbazone (4-PT), a potent and secure tyrosinase inhibitor and anti-browning agent, was synthesized.^54^

Molecular hybridization, the basis for drug development, is the process of fusing two or more active molecules to produce a new molecule that can inherit the beneficial structure of its parent molecule. This approach may boost the potency of the molecule. So, to keep striving towards the synthesis of potent tyrosinase inhibitors,^55–57^ we report herein the synthesis of novel *N*-benzyl indole-based thiosemicarbazones by the incorporation of *N*-benzyl indole with thiosemicarbazide derivatives. These compounds were also investigated for their potency against tyrosinase enzyme. Molecular docking, ADMET, kinetics, and structure–activity relationships were also studied. Tyrosinase is a multi-copper enzyme usually distributed in different organisms and plays a significant role in melanogenesis and enzymatic browning. Therefore, inhibitors of tyrosinase can be beneficial in cosmetics as well as medicinal industries due to its depigmentation nature, and also in food and agriculture industries as anti-browning compounds.^58^ Molecular docking is a versatile computational technique that has the potential to predict the binding affinity of a ligand to its corresponding receptor proteins. Glide docking studies (Table S1†) have been extensively employed to explore these ligand–receptor interactions.^59^ The use of molecular docking investigations will enable the identification of potential inhibitors^60^ and furnish an avenue to explore the binding mechanism of the inhibitors with the target protein. By comparing the efficacy of the newly discovered inhibitors with the standard drug, and the co-crystallized ligand, we can evaluate their probable role in tyrosinase enzyme inhibition.

## 2. Results and discussion

### 2.1. Chemistry

Indole and thiosemicarbazone skeletons are linked to various biological and pharmacological activities, therefore it's logical to incorporate the two moieties into a single molecular frame to synthesize more powerful biologically active molecules, like 5(a–r).^61^ The target molecules have been obtained by the modification at the N–H position of indole 3 carbaldehyde. The modification mainly includes the substitution by benzylation (benzyl bromide) in place of hydrogen at N–H of indole and the generation of thiosemicarbazones at C-3 with thiosemicarbazide to elevate its biological activity.

**Scheme 1:**
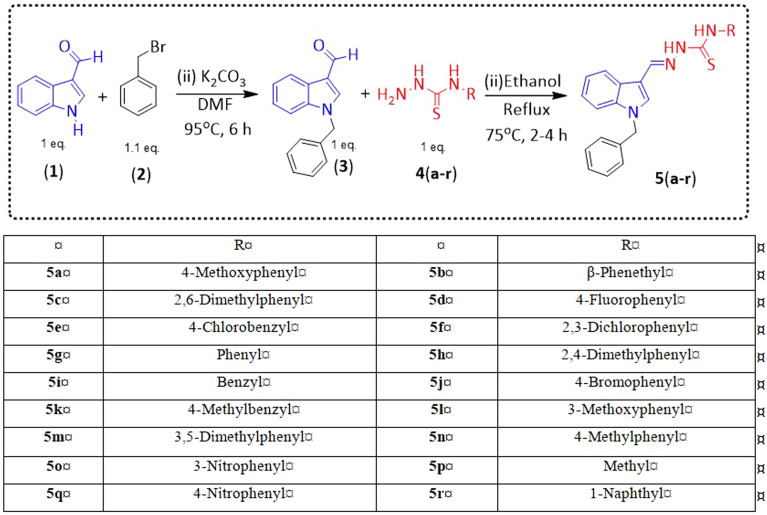
Synthetic route for the preparation of thiosemicarbazones.

The targeted compounds 5(a–s) were accomplished by the substitution at the N–H position which was done by the reaction of indole 3-carbaldehyde (1) with benzyl bromide (2) using anhydrous K_2_CO_3_ and DMF at 90 °C for 6 h, gives *N*-benzyl 3-formyl indole (3). The synthesis of thiosemicarbazones at C-3 was followed by the reaction of compound (3) with thiosemicarbazides 4(a–r). The synthetic route in the synthesis of 1-benzyl indole-based thiosemicarbazones 5(a–r) was displayed in Scheme 1. All the derivatives were characterized by various advanced spectroscopic techniques. A singlet is seen at 5.45–5.49 ppm which can be attributed to the CH_2_ group of the benzyl substitution at indole. A singlet is observed at 8.47–7.99 ppm range which can be attributed to the azomethine hydrogen, finalizing the condensation with aldehydes. NH hydrazine is also a significant signal found as a singlet in the 11.99–11.19 ppm range.

### 2.2. Pharmacology

#### 2.2.1. Biological activity

Following the above-described technique, the inhibitory activity of each target molecule was assessed on tyrosinase utilizing kojic acid as a positive control. The data of inhibitory activity expressed as an IC_50_ value, was displayed in Table 1. The results indicated that the target compounds displayed IC_50_ in the range of 12.40 ± 0.26 μM to 47.24 ± 1.27 μM. The dose response curves of all compounds (5a–r) are presented in Fig. S1.† Among the target compounds, 5k, 5q, 5f, 5d, and 5o exhibited exceptional potential than the standard, kojic acid with an IC_50_ value of 18.30 ± 0.41 μM. Derivative 5k displayed the highest inhibitory activity against tyrosinase enzyme with IC_50_ = 12.40 ± 0.26 μM, followed by 5q with (IC_50_ = 15.26 ± 0.30 μM), 5f (IC_50_ = 15.28 ± 0.37 μM), 5d (IC_50_ = 16.76 ± 0.38 μM) and 5o (IC_50_ = 17.10 ± 0.28 μM). Compound 5a showed lower activity than the standard followed by 5n 5j, 5c, 5h, 5b, and 5l which showed almost similar inhibitory activities with IC_50_ ranging from 26.11 ± 0.47 μM to 31.64 ± 1.26 μM. Compounds 5e, 5r, 5i, 5g, 5b, 5m and 5r showed much lower activities than kojic acid with IC_50_ values as follows:

**Table 1:** Structures, yield and IC_50_ values of the synthesized compounds against tyrosinase^a^

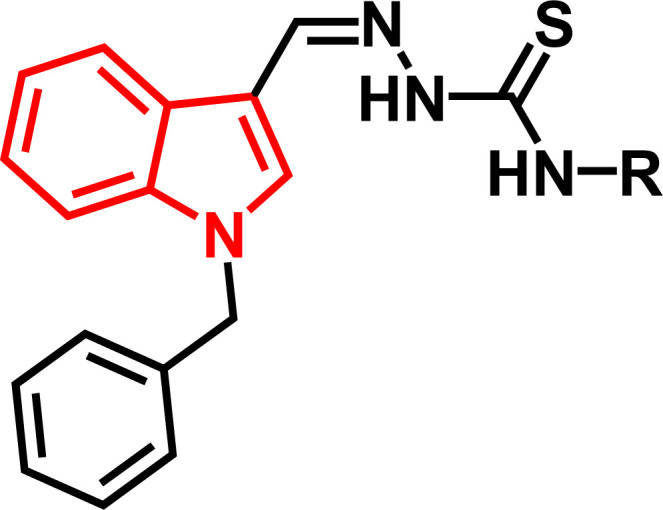				
Compounds	Structure	Yield (%)	Percent inhibition (0.5 mM)	IC_50_ ± μM (SEM)
5a	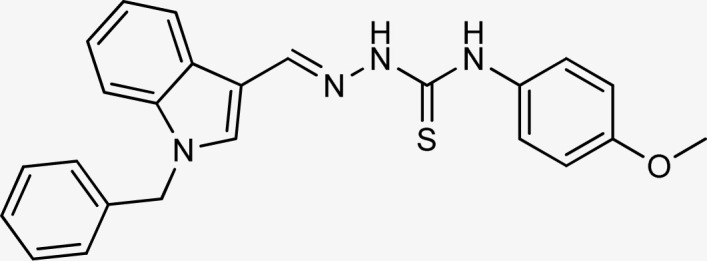	81	87.15	24.16 ± 0.38
5b	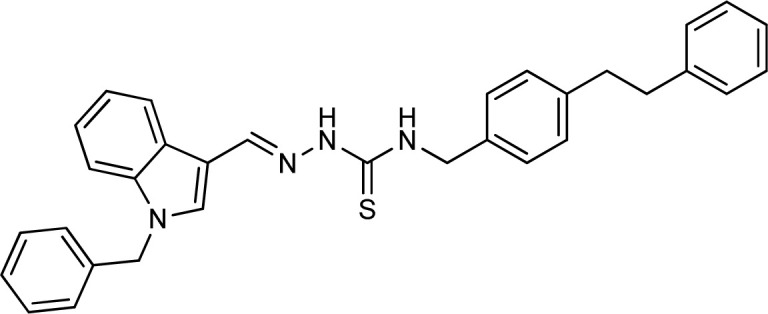	87	78.35	45.38 ± 0.75
5c	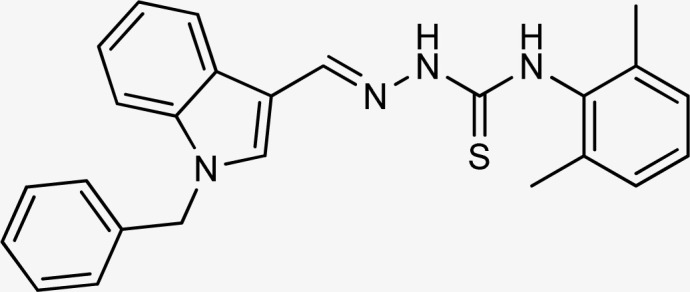	92	85.66	27.40 ± 0.60
5d	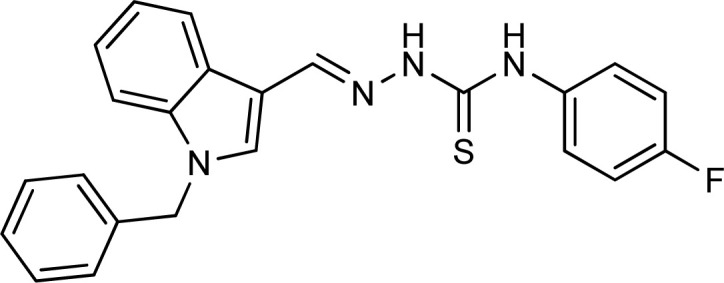	83	90.47	16.76 ± 0.38
5e	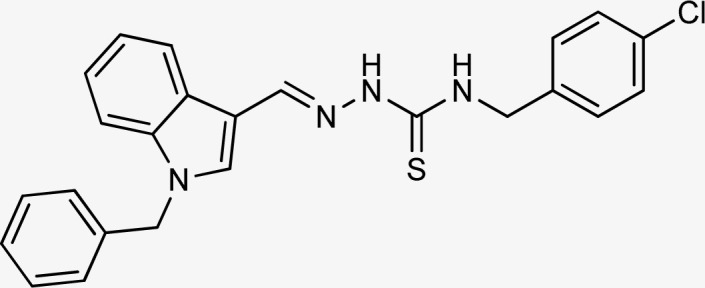	77	84.73	33.64 ± 1.10
5f	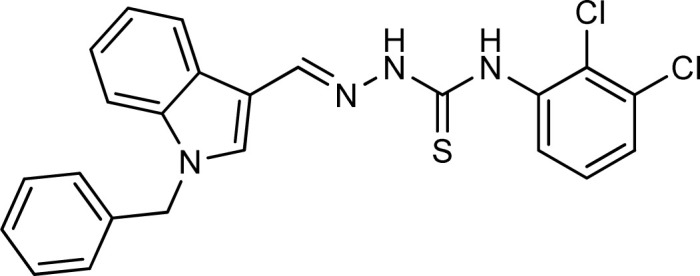	84	90.83	15.28 ± 0.37
5g	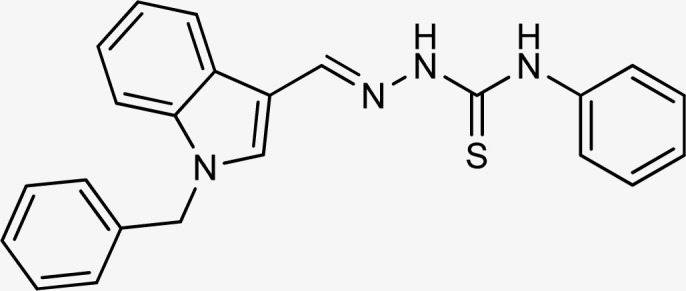	87	81.47	38.26 ± 1.34
5h	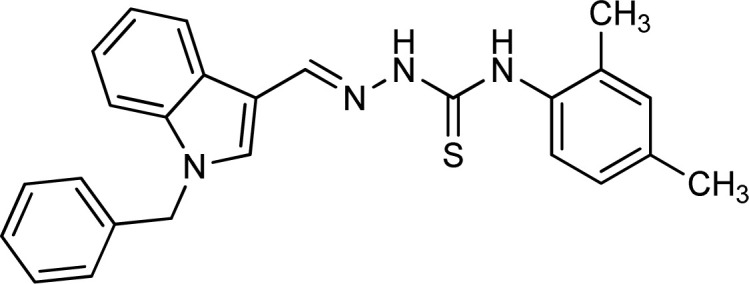	86.5	83.76	29.71 ± 0.72
5i	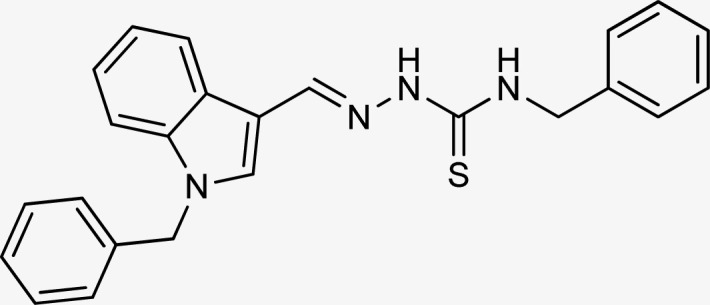	66	82.71	36.72 ± 1.48
5j	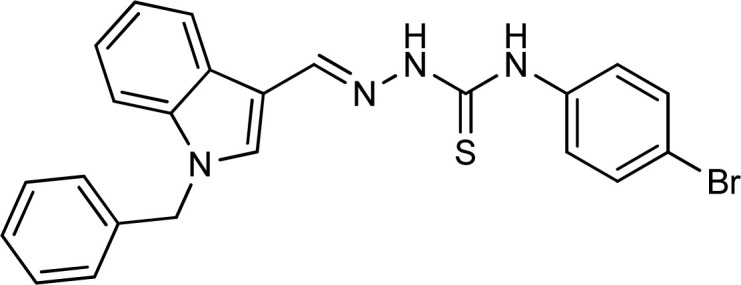	100	84.19	26.83 ± 0.29
5k	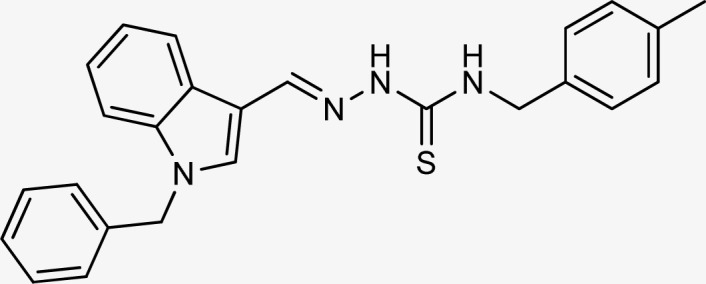	87	90.84	12.40 ± 0.26
5l	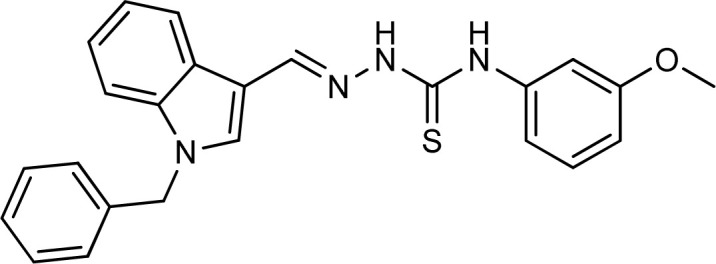	75	84.39	31.64 ± 1.26
5m	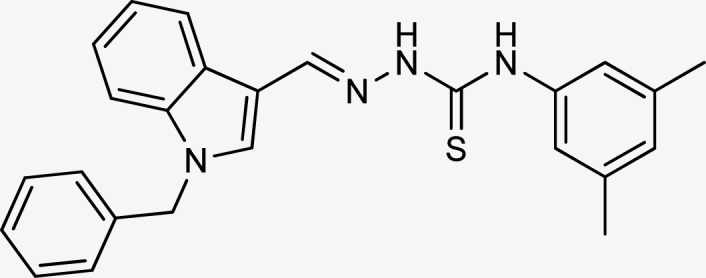	59	76.35	47.24 ± 1.27
5n	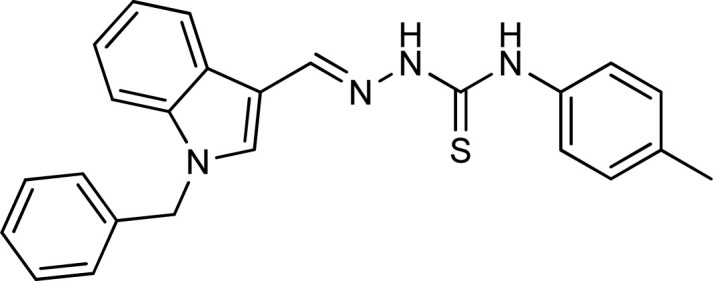	72	86.54	26.11 ± 0.47
5o	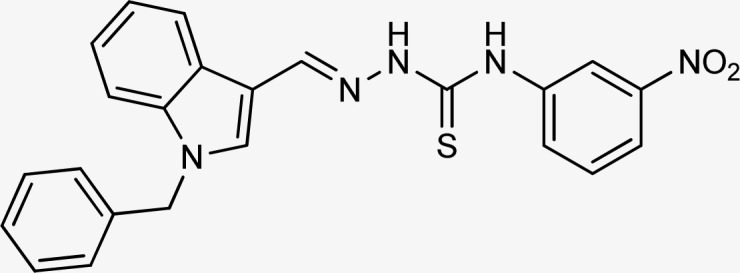	89	91.34	17.10 ± 0.28
5p	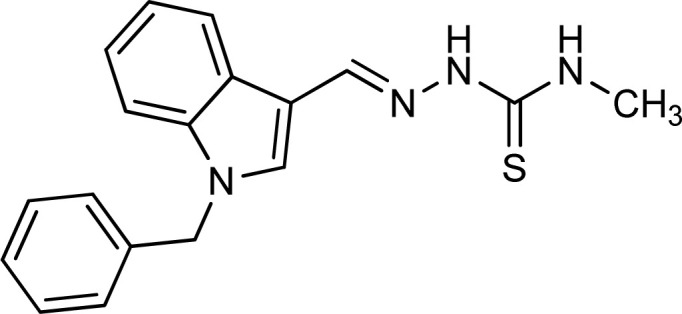	72	46.20	N/A
5q	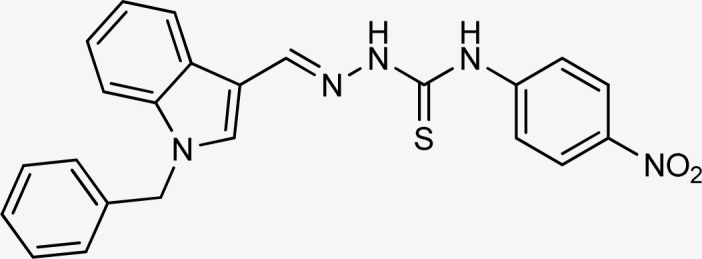	67	91.53	15.26 ± 0.30
5r	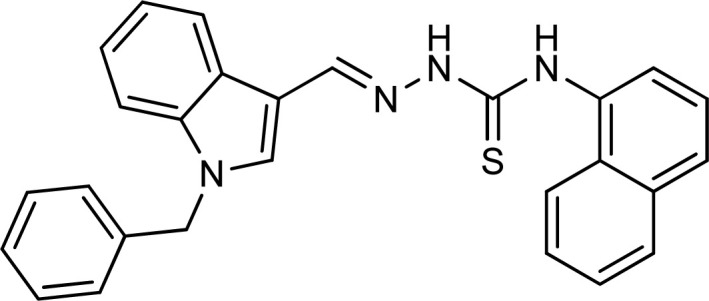	66	77.86	34.58 ± 0.81
Standard	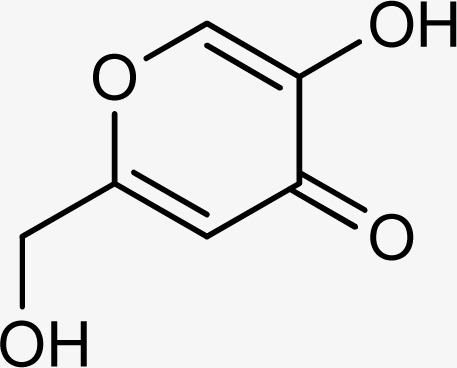		Kojic acid	18.30 ± 0.41

a Reaction conditions (i) K_2_CO_3_, DMF, 95 °C, 6 h (ii) ethanol, 75 °C reflux, 2–4 h.

5e IC_50_ = 33.64 ± 1.10 μM

5r IC_50_ = 34.58 ± 0.81 μM

5i IC_50_ = 36.72 ± 1.48 μM

5g IC_50_ = 38.26 ± 1.34 μM

5b IC_50_ = 45.38 ± 0.75 μM

Compound 5m was the least potent member of the series with an IC_50_ value of 47.24 ± 1.27 μM.

#### 2.2.2. Structure–activity relationship

Because the thiosemicarbazide scaffold was able to successfully combine the two copper ions at the tyrosinase active site, the SAR tests demonstrated that it was a crucial component for determining the tyrosinase inhibitory action. Several 1-benzyl substituted indole-based thiosemicarbazones were reported for their inhibitory action against mushroom tyrosinase in an attempt to increase the activity. The inhibitory potential of the thiosemicarbazones is explored by changing the R group on the thiosemicarbazide moiety.

In a study by Thanigaimalai *et al.* designed a series of naphthaldehyde-based thiosemicarbazones, the finding suggested that the hydrophobicity of the substituent on hydrazine had a significant role in the inhibitory effect against melanogenesis. Moreover, the activity was greatly enhanced when either of these hydrogens was replaced at N^1^ or N^3^.^62^ Based on this study, we will investigate the effect of substitutions of varying hydrophobicity on the inhibitory potential against tyrosinase.

The R group is varied with aromatic, non-aromatic, and aliphatic groups. The compounds 5i and 5g with benzyl and phenyl rings respectively showed almost similar inhibitory potential. The inhibitory potential is further collapsed in compound 5r when 1-naphthyl is used. Interestingly in compound 5p when methyl group is used, no inhibitory activity is seen. On increasing the hydrophobicity from methyl to phenyl and benzyl, a significant increase can be seen in the potency. While increasing it further to naphthyl substitution, the potency is collapsed which can be attributed to the steric hindrance with the active site of the tyrosinase enzyme.

**Fig. 2:**
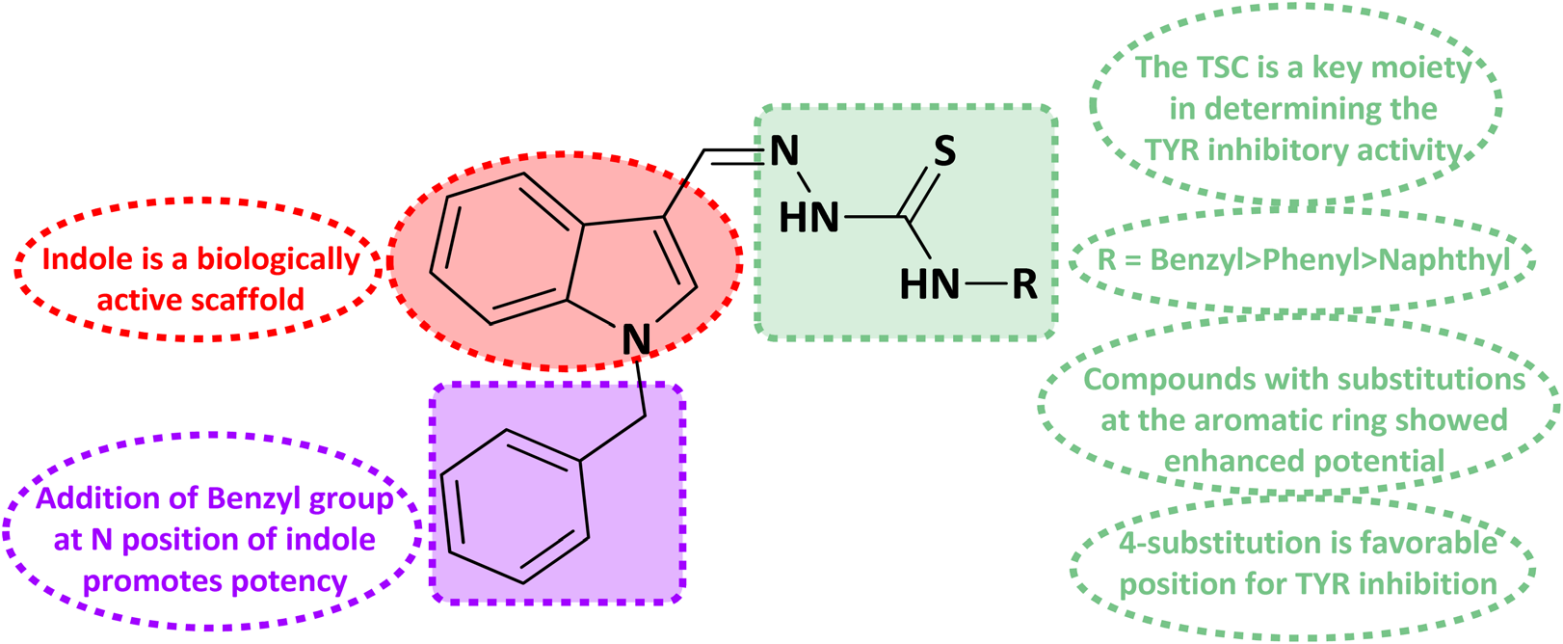
Structure–activity relationship of the synthesized thiosemicarbazones.

The compounds 5q and 5o with a nitro group substitution on the phenyl ring displayed excellent inhibitory potency better than the reference standard kojic acid (IC_50_ = 18.30 ± 0.41 μM). However, a nitro group at the *para* position 5q (IC_50_ = 15.26 ± 0.30 μM) slightly outshined 5o (IC_50_ = 17.10 ± 0.28 μM) with the nitro group at the *meta* position. Compounds 5a and 5l containing methoxy substitution at the *para* and *meta* positions of the phenyl ring respectively also followed the same pattern. 5a showed better activity with IC_50_ value 24.16 ± 0.38 μM than 5l with IC_50_ value of 31.64 ± 1.26 μM. In the case of methyl substitution on the phenyl ring, the compound 5n with methyl substitution at the *para* position (IC_50_ = 26.11 ± 0.47 μM) put on similar inhibitory potency against tyrosinase as the kojic acid. Interestingly with the increase in no. of substitutions, potency is somewhat decreased. Compound 5c with 2,6 dimethyl substitution (IC_50_ = 27.40 ± 0.60 μM) and 5h with 2,4 dimethyl substitution (IC_50_ = 29.71 ± 0.72 μM) showed moderately lower activity than 5n with 4 methyl substitution. While in compound 5m with 3,5 dimethyl substitutions, activity showed a steep decline with an IC_50_ value of 47.24 ± 1.27 μM. In the case of our study, a certain relationship can be seen between the inhibition and the structure as shown in Fig. 2. In all of the above-mentioned cases, compounds with *p*-substitution showed superior potency.

In the case of halogen-substituted derivatives, compound 5d, which is the third most potent derivative of the series with *p*-fluoro substitution on the phenyl ring displayed higher potency than 5j containing *p*-bromo substitution. The result is consistent with the findings of Liu *et al.* who synthesized novel thiourea derivatives with sulfur-containing heterocyclic scaffolds. The study also reported that the compounds containing fluorine atoms at the phenyl ring exhibited higher tyrosinase inhibitory activities.^63^

Compound 5k with 4-methyl benzyl substitution displayed the highest potency in the entire series. While compound 5e with 4-chloro benzyl substitution displayed moderately lower activity. It can be seen that with the increase in electronegativity of the substituents, inhibitory potential is decreased. In the case of unsubstituted benzyl in compound 5i, activity is lower than both the substituted derivatives 5k and 5e. It may be suggested that the substitutions favor inhibitory potency.

### 2.3. Kinetics

**Fig. 3:**
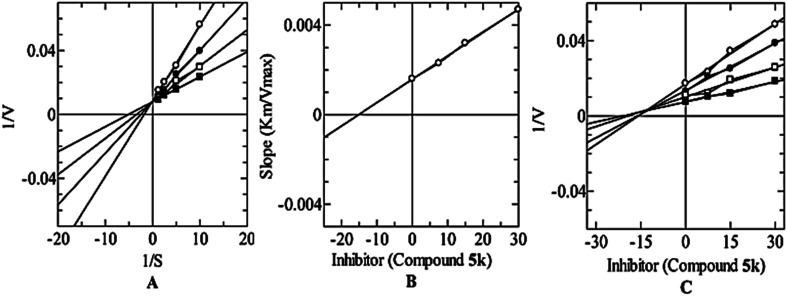
Mode of inhibition of tyrosinase by compound 5k (A) Lineweaver–Burk plot of reciprocal of rate of reaction (V) *vs.* reciprocal of the substrate (l-Dopa) in the absence of (■), and in the presence of 30.00 μM (○), 15.00 μM (●), and 7.50 μM (□), of compound 5k (B) secondary replot of LineWeaver–Burk plot between the slopes of each line on LineWeaver–Burk plot *vs.* different concentrations of compound 5k (C) Dixon plot of reciprocal of rate of reaction (V) *vs.* different concentrations of compound 5k.

A kinetic study of the most potent compound 5k was performed to explore its mechanism of action. Derivative 5k was identified as a competitive type of inhibitor with a *K*_i_ value of 10.20 ± 0.006 μM. Such type of inhibitor binds with the active site residues of the tyrosinase hence, usually, this type of inhibition increases the *K*_m_ value of the enzyme while there is no effect on the *V*_max_ value of the enzyme and remains constant (Fig. 3).

### 2.4. Molecular docking

Molecular docking is a worth mentioning method to evaluate the binding affinity, as well as the interaction parameters of investigated ligands in this study. This tool presents significant insights into the intended binding capabilities of the ligands under consideration with the specified target protein.^64^ The glide module was incorporated to execute a molecular docking study, and the outcomes were then assessed based on corresponding glide score values as shown in Table 2 for the hit compounds 5k, the standard drug kojic acid, and the co-crystallized ligand of the chosen tyrosinase target protein, the glide score data of all the studied ligands is presented in (Table S2†).

**Table 2:** gScore, Emodel, HBI residues, polar and hydrophobic amino acid residues for the hit compound (5k), the standard drug (kojic acid), and the co-crystallized ligand along with the RMSD values of 5k, and the standard drug (kojic acid) against the co-crystallized ligand^a^

Ligands	gScore (kcal mol^−1^)	Emodel (kcal mol^−1^)	HBI residue (distance Å)	Polar interacting amino acids	Hydrophobic interacting amino acid residues	RMSD (Å)
5k	−5.157	−63.013	Asp357 (2.02)	Gln307, Thr308, Thr360, Ser364	Ile17, Tyr311, Trp358, Phe368, Val371	2.71
Kojic acid	−4.549	−34.051	Thr308 (1.96), Asp312 (2.00), Glu356 (2.08)	Gln307, Thr308	Ala304, Tyr311, Tyr314, Trp358	3.08
Co-crystallized ligand	−0.129	−19.187	Asp357 (1.94), Glu359 (1.98), Lys379 (1.85)	Ser364	Trp358, Phe368, Val371	

a Abbreviation: HBI, hydrogen bonding interactions.

The poses retrieved after docking were examined visually, and the associated binding interactions of the synthesized compounds with the binding pocket residues were analyzed with the help of ligand–interaction diagrams (3D & 2D). The docking scores are tabulated as negative values, the lower the docking score value and glide energy value, the higher would be the binding affinity and *vice versa*. It was thus established that most of the reported compounds displayed high glide docking scores against the chosen target protein under study and all the synthesized compounds are superior in binding affinity as compared to the co-crystallized ligand, whereas the compounds 5k, 5c, 5b, and 5a are even better than the standard drug (kojic acid) under investigation as displayed in Table S1.†

**Fig. 4:**
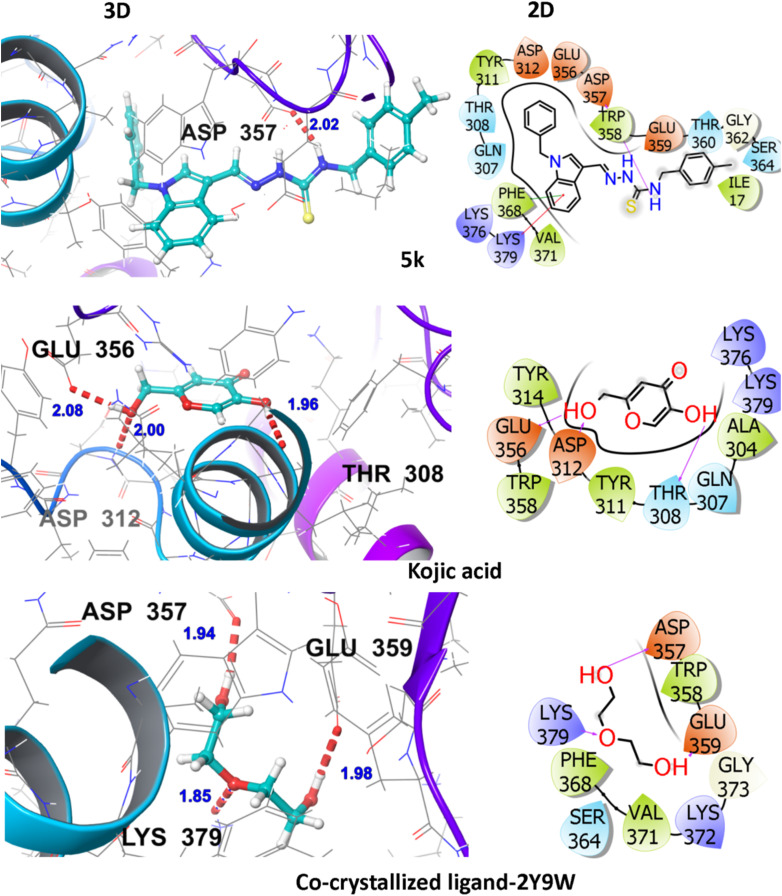
3D and 2D visualization of compound-5k, kojic acid and co-crystalized ligand with target protein (PDB ID: 2Y9W).

The hit compound 5k showed an appreciable binding interactive profile with tyrosinase target protein having a gScore of −5.157 kcal mol^−1^. Asp357 (2.02 Å) amino acid residue is engaged in hydrogen bonding and Gln307, Thr308, Thr360, and Ser364 are the polar interacting amino acid residues. Ile17, Tyr311, Trp358, Phe368, and Val371 are the hydrophobic interacting residues. The hydrophobic interactions are of vital significance for drug-target binding as they are involved in stabilization of the ligand at the binding interface. The visual display of standard drug-kojic acid for the said tyrosinase inhibition activity shows a gScore of −4.549 kcal mol^−1^. It establishes hydrogen bonding with Thr308 (1.96 Å), Asp312 (2.00 Å), and Glu356 (2.08 Å). In addition, Ala304, Tyr311, Tyr314, Trp358 are hydrophobically engaged amino acids whereas Gln307, and Thr308 are polar amino acids as shown in Fig. 4.These interactions highlight the consistent binding pattern between the hit compound (5k), the standard drug (kojic acid) and the co-crystallized ligand. These findings thus establishes that the 5k exhibits the superior tyrosinase inhibition activity as compared to the standard drug and the co-crystallized ligand among all the investigated ligands. The Asp357 is participant in hydrogen bonding in the co-crystallized ligand and the hit compound, 5k whereas the Thr308 is commonly observed polar contacts in both 5k and the standard drug whereas the Ser364 is found in both the 5k and co-crystallized. Furthermore, as far as the hydrophobic profile is concerned, Tyr311 is commonly visualized in both the 5k and the standard drug, whereas Phe368, and Val371 are the common residues in the 5k and the co-crystallized. Trp358 is commonly viewed hydrophobic interactions in all these investigated compounds. Fig. S2† further highlights various interactions in the other hit compounds.

### 2.5. Validation of the docking protocol

For each ligand–reference pair, whenever a pose is classified as a good solution, it predicts that the scoring function regenerates the crystallographic binding orientation.

**Fig. 5:**
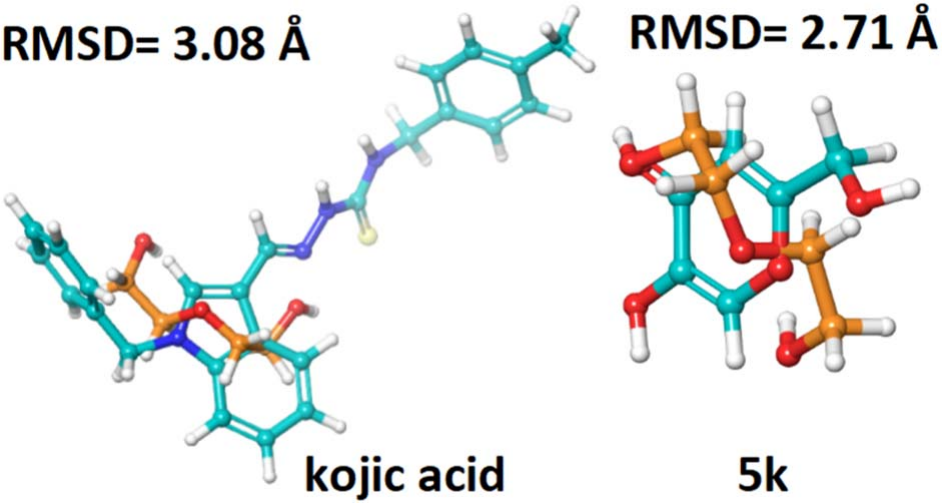
RMSD values of the hit compound (5k), and the reference compound kojic acid (cyan color) interacting with the co-crystallized ligand (orange color).

The RMSD values for the hit compound are within the acceptable limit of less than 3 Å, with 5k at 2.71 Å. The reference compound kojic acid touches the standard limit, with an RMSD of 3.08 Å as seen in Table 2 and Fig. 5.

### 2.6. ADMET analysis

**Fig. 6:**
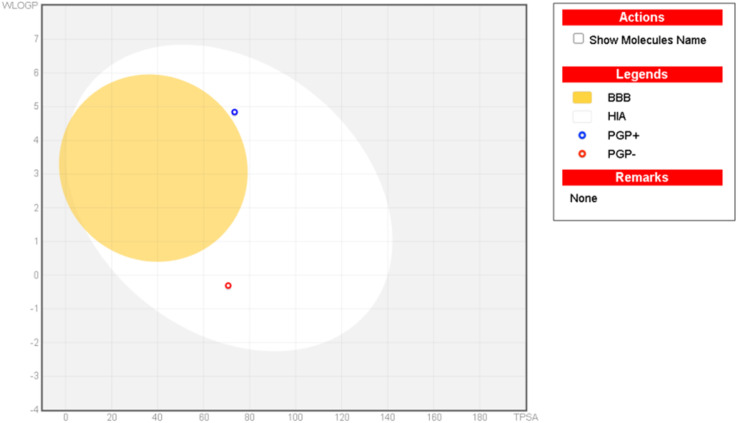
Boiled-egg plot (plot of WLOGP against TPSA) of the hit compound-5k and standard-kojic acid from SwissADME.

Our aims and objectives emphasized to assess the ADMET properties of the investigated compounds, the hit compound 5k is showing good binding interactions with the target receptor in glide molecular docking assay along with the ADMET profile of standard drug, kojic acid as presented in Table 3 while the detailed ADMET properties of all investigated ligands are shown in Table S3†. The compound (5e) demonstrated considerable lipid solubility followed by compounds 5i, and 5a (Table S3†) with XLOGP3 as high as 6.09. It was then evaluated that the investigated compounds exhibit poor absorption when their TPSA is greater than 140 Å^2^, and all the investigated compounds showed compliance with this standard. In addition, all these compounds have no more than 5 HBDs, no more than 10 HBAs, and molecular weight is less than 500 dalton. The favorable “drug-likeness” is adhered to Lipinski, Ghose, Veber, and Egan rules with a zero-violation trend, shown by most of these compounds. The synthesized compounds under study possess a high likelihood of passive absorption through the gastrointestinal tract with 0.55 bioavailability, and they do not cross the blood–brain barrier (except compound 5p). In addition, these compounds are not the substrates for *p*-glycoprotein, which is a key factor in drug efflux except for the compounds (5b, 5c, 5e, 5i, and 5k). Most of these compounds showed inhibition of CYP enzymes, thus exhibiting a significant interactive profile with drug-metabolizing enzymes, and associated toxicity (Table 3). In addition, all these compounds have no more than 5 HBDs, no more than 10 HBAs, and a corresponding molecular weight is less than 500 dalton presenting them the suitable drug candidates. In addition, the boiled-egg plot was executed to validate the GI absorption, as well as the BBB permeation characteristics of the hit compound 5e, experimentally proved hit compound 5k, and standard kojic acid. In addition, it is established from the plot that none of the compounds has passed through the BBB but they lie within the GI absorption premises (Fig. 6). Fig. S3† shows that compound 5p is the only compound from the investigated list of compounds that can cross the BBB.

**Table 3:** The ADMET properties showing absorption, bioavailability, metabolism, lipinski violations, and synthetic accessibility of hit ligands and kojic acid (standard drug)^a^

			Absorption					Metabolism						
Ligand	TPSA	ESOL LogS	XLOGP3	GI absorption	BBB	P-gp substrate	BA	CYP1A2 inhibitor	CYP2C19 inhibitor	CYP2C9 inhibitor	CYP2D6 inhibitor	CYP3A4 inhibitor	Lipinski # violations	SA
Kojic acid	33.13	−0.7	−0.64	High	No	No	0.55	No	No	No	No	No	0	2.53
5k	73.44	−5.62	5.13	High	No	Yes	0.55	Yes	Yes	Yes	Yes	Yes	0	3.3

a Abbreviations: TPSA, topological polar surface area; BBB, blood–brain barrier; BA, bioavailability; SA, synthetic accessibility; ADMET, absorption, distribution, metabolism, excretion, and toxicity; GI, gastrointestinal tract.

## 3. Conclusion

In the current study, a series of *N*-benzyl indole-based thiosemicarbazones were designed, synthesized, and characterized by spectroscopic techniques (^1^H NMR and ^13^C NMR). The derivates were screened for their inhibition potential against tyrosinase enzyme. All synthesized compounds present an outstanding to moderate inhibition potency compared to kojic acid as a positive control. These encouraging findings may lead to preclinical testing of compounds 5k in the hopes of creating a novel class of tyrosinase inhibitors. Because they address the need for effective and safe therapies for abnormal pigmentation and associated health risks, these results have implications for the pharmaceutical and cosmetics sectors.

## 4. Experimental

### 4.1. Chemistry

#### 4.1.1. General

For the synthesis of 1-benzyl indole-based thiosemicarbazones, all the starting materials were bought from Sigma-Aldrich. Chemicals and solvents including ethanol, methanol, glacial acetic acid, petroleum ether, and ethyl acetate were bought from Merck and used in their original form. Silica gel plates with aluminium backs were utilized to check the reaction progress and completion. A Bruker Ascend 600 MHz NMR spectrometer was operated to get 1-H and 13-C NMR spectra in deuterated solvents like CDCl_3_ and DMSO-*d*_6_ at 25 °C (600 MHz for 1H and 151 MHz for 13-C). NMR spectra were presented as chemical shifts (ppm), and coupling constants (*J*) were demonstrated in Hertz (Hz) to detail signal multiplicity. HPLC was carried out on Agilent, Germany (Liquid Chromatographic Column 150 mm × 4.6 mm (id) packed with 5-micron C18; 263 nm), and the mass spectrum was recorded on QTOF MS 6530 WITH 1260 HPLC.

#### 4.1.2. General method for the synthesis of 1-benzyl-1*H*-indole-3-carbaldehyde (3)

A mixture of indole-3-carbaldehyde (1) (10 mmol), benzyl bromide (2) (10.85 mmol), and anhydrous K_2_CO_3_ (1.4 g) in DMF (10 mL) was robustly stirred and refluxed for 6 h. After the disappearance of indole-3-carbaldehyde (1) as checked by TLC. The reaction mixture was cooled down and poured into ice-cold water. The solid precipitates formed (3) were filtered, washed with water, dried out, and recrystallized from ethyl alcohol to afford aldehyde (3) in 91% yield.

#### 4.1.3. General method for the synthesis and characterization of compounds 5(a–r)

A mixture of 1-benzyl-1*H*-indole-3-carbaldehyde (3) (0.1 g, 4.2 mmol) and thiosemicarbazide derivatives (4.2 mmol) 4(a–r) was refluxed at 75 °C in 10 mL ethanol for 2–4 h with catalytic amount (2–3 drops) of acetic acid. The progress of the reaction was checked with TLC (1 : 1 petroleum ether and ethyl acetate as eluent). The solid formed was filtered, washed with ethanol, and dried to yield targeted thiosemicarbazones 5(a–r). Characterization data of each compound synthesized is given below:

##### 4.1.3.1 (*E*)-2-[(1-benzyl-1*H*-indol-3-yl)methylene]-*N*-(4-methoxyphenyl)hydrazinecarbothioamide (5a)

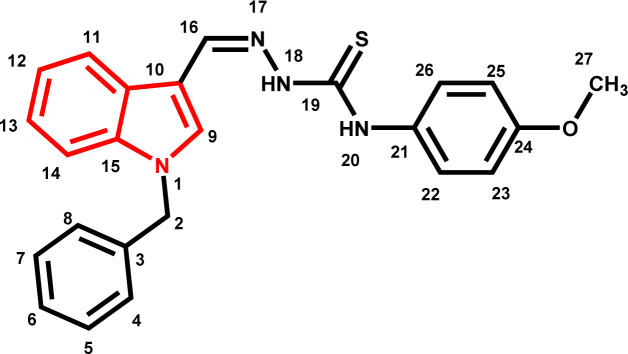



Color: off-white, yield: 81%, melting point: 217–218 °C. *δ*_H_ (600 MHz, DMSO-*d*_6_) 11.51 (1H, s, H-18), 9.50 (1H, s, H-20), 8.41 (1H, s, H-9), 8.28 (1H, d, *J* = 7.8 Hz, Ar-H), 8.08 (1H, s, H-16), 7.53 (1H, d, *J* = 8.1 Hz, Ar-H), 7.49–7.43 (2H, m, Ar-H), 7.33 (2H, t, *J* = 7.5 Hz, Ar-H), 7.30–7.25 (3H, m, Ar-H), 7.22 (1H, t, *J* = 7.6, Ar-H), 7.17 (1H, t, *J* = 7.5 Hz, Ar-H), 6.98–6.91 (2H, m, H-23, 25), 5.47 (2H, s, N-CH_2_), 3.78 (3H, s, –OCH_3_); ^13^C NMR (151 MHz, DMSO) *δ* 175.55 (C-19), 157.30 (C-16), 140.91 (CH), 137.92 (C), 137.36 (C), 134.56 (CH), 132.70 (C), 129.12 (C-5, 7), 128.07 (CH), 127.71 (C-4, 8), 127.65 (CH), 125.22 (C), 123.36 (CH), 122.72 (CH), 121.50 (CH), 113.79 (CH), 111.18 (C), 111.15 (C), 55.74 (C-2), 49.89 (C-27). HPLC: CH_3_CN : H_2_O = 80 : 20; *t*_R_: 2.051 min, purity: 99.3%. TOF MS ES+ (*m*/*z*): [M + H]^+^, calcd: 415.1592, found: 415.1598, anal. calcd for C_24_H_22_N_4_OS: C, 69.54; H, 5.35; N, 13.52; found: C, 69.59; H, 5.42; N, 13.58.

##### 4.1.3.2 (*E*)-2-[(1-benzyl-1*H*-indol-3-yl)methylene]-*N*-phenethylhydrazinecarbothioamide (5b)

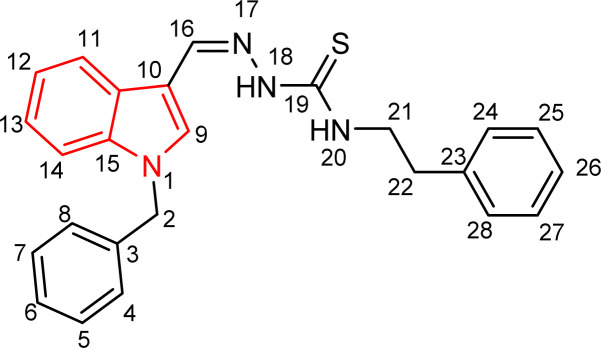



Color: white, yield: 87%, melting point: 198–201 °C. *δ*_H_ (600 MHz, DMSO-*d*_6_) 11.27 (1H, s, H-18), 8.31 (1H, s, H-9), 8.05 (1H, d, *J* = 7.9 Hz, Ar-H), 7.99 (1H, s, H-16), 7.87 (1H, t, *J* = 5.8 Hz, H-20), 7.52 (1H, d, *J* = 8.2 Hz, Ar-H), 7.33 (6H, dd, *J* = 10.0, 5.7 Hz, Ar-H), 7.29–7.21 (5H, m, Ar-H), 7.17 (1H, t, *J* = 7.5 Hz, Ar-H), 5.45 (2H, s, H-2), 3.88 (2H, q, *J* = 6.7 Hz, H-21), 2.96 (2H, t, *J* = 7.3 Hz, H-22); ^13^C NMR (151 MHz, DMSO) *δ* 176.40 (C-19), 140.62 (C-16), 139.76 (CH), 137.91 (C), 137.38 (C), 134.56 (CH), 129.11 (C-5, 7), 129.09 (CH), 128.97 (CH), 128.06 (CH), 127.66 (C-4, 8), 126.70 (CH), 125.01 (C), 123.37 (CH), 122.67 (CH), 121.49 (CH), 111.23 (C), 111.14 (C), 49.85 (C-2), 45.25 (C-21), 35.38 (C-22). HPLC: CH_3_CN : H_2_O = 80 : 20; *t*_R_: 1.814 min, purity: 99.3%. TOF MS ES+ (*m*/*z*): [M + H]^+^, calcd: 413.1799, found 413.1798, anal. calcd for C_25_H_24_N_4_S: C 72.78; H, 5.86; N, 13.58; found: C, 72.83; H, 5.92; N, 13.61.

##### 4.1.3.3 (*E*)-2-[(1-benzyl-1*H*-indol-3-yl)methylene]-*N*-(2,6-dimethylphenyl)hydrazinecarbo thioamide (5c)

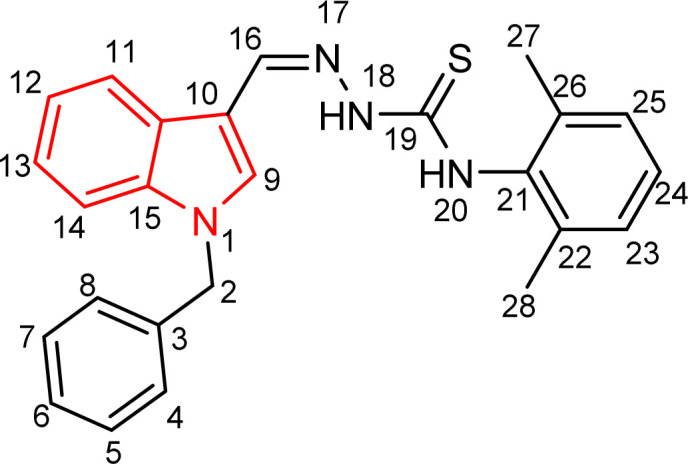



Color: white, yield: 92%, melting point: 225–227 °C. *δ*_H_ (600 MHz, DMSO-*d*_6_) 11.45 (1H, s, H-18), 9.28 (1H, s, H-20), 8.42 (1H, s, Ar-H), 8.40 (1H, s, H-9), 8.06 (1H, s, H-16), 7.50 (1H, d, *J* = 8.2 Hz, Ar-H), 7.33 (2H, t, *J* = 7.4 Hz, Ar-H), 7.26 (3H, d, *J* = 7.7 Hz, Ar-H), 7.21 (1H, t, *J* = 7.6 Hz, Ar-H), 7.15–7.10 (4H, Ar-H), 5.47 (2H, s, H-2), 2.23 (6H, s, H-27, 28); ^13^C NMR (151 MHz, DMSO) *δ* 176.12 (C-19), 140.80 (C-16), 138.11 (C), 137.99 (C), 137.35 (C), 137.16 (CH), 134.62 (CH), 129.10 (CH), 128.04 (CH), 128.00 (CH), 127.58 (CH), 127.24 (CH), 125.17 (C), 123.32 (CH), 121.38 (CH), 111.35 (C), 110.97 (C), 49.85 (C-2), 18.66 (C-27, 28). HPLC: CH_3_CN : H_2_O = 80 : 20; *t*_R_: 2.444 min, purity: 100.0%. TOF MS ES+ (*m*/*z*): [M + H]^+^, calcd: 413.1799, found: 413.1799, anal. calcd for C_25_H_24_N_4_S: C, 72.78; H, 5.86; N, 13.58; found: C, 72.83; H, 5.92; N, 13.60.

##### 4.1.3.4 (*E*)-2-[(1-benzyl-1*H*-indol-3-yl)methylene]-*N*-(4-fluorophenyl)hydrazinecarbothioamide (5d)

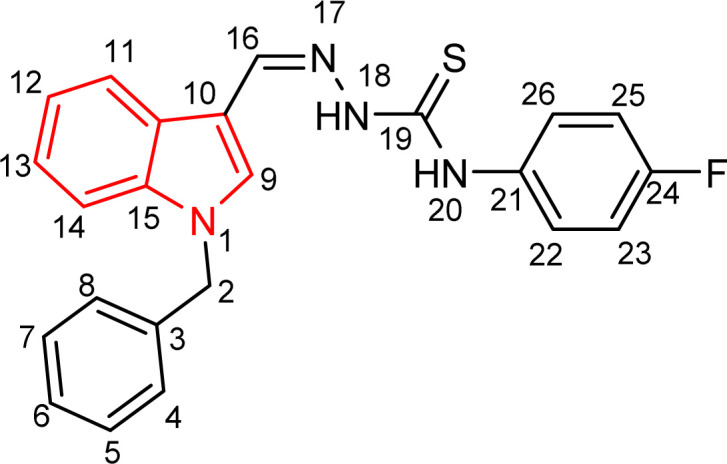



Color: white, yield: 83%, melting point: 221–223 °C. *δ*_H_ (600 MHz, DMSO-*d*_6_) 11.61 (1H, s, H-18), 9.62 (1H, s, H-20), 8.42 (1H, s, H-9), 8.29 (1H, d, *J* = 7.9 Hz, Ar-H), 8.10 (1H, s, H-16), 7.60 (2H, dt, *J* = 8.8, 3.7 Hz, Ar-H), 7.53 (1H, d, *J* = 8.3 Hz, Ar-H), 7.36–7.31 (2H, m, Ar-H), 7.28–7.27 (3H, m, Ar-H), 7.24–7.16 (4H, m, Hz, Ar-H), 5.48 (2H, s, H-2); ^13^C NMR (151 MHz, DMSO) *δ* 175.05 (C-19), 160.36 (C-16), 158.76 (d, *J*_C–F_ = 241.6 Hz, C-24), 140.85 (CH), 137.47 (C), 136.93 (C), 135.74(CH), 134.28 (CH), 128.68 (C-5, 7), 127.79 (CH), 127.64 (CH), 127.21 (CH), 124.78 (C), 122.94 (CH), 122.35 (CH), 121.08 (CH), 114.80 (CH), 114.65 (C), 110.70 (C), 49.46 (C-2). HPLC: CH_3_CN : H_2_O = 80 : 20; *t*_R_: 2.204 min, purity: 99.7%. TOF MS ES+ (*m*/*z*): [M + H]^+^, calcd: 403.1392, found: 403.11395, anal. calcd for C_23_H_19_FN_4_S: C, 68.63; H, 4.76; N, 13.92; found: C, 68.69; H, 4.83; N, 13.99.

##### 4.1.3.5 (*E*)-2-[(1-benzyl-1*H*-indol-3-yl)methylene]-*N*-(4-chlorobenzyl)hydrazinecarbothioamide (5e)

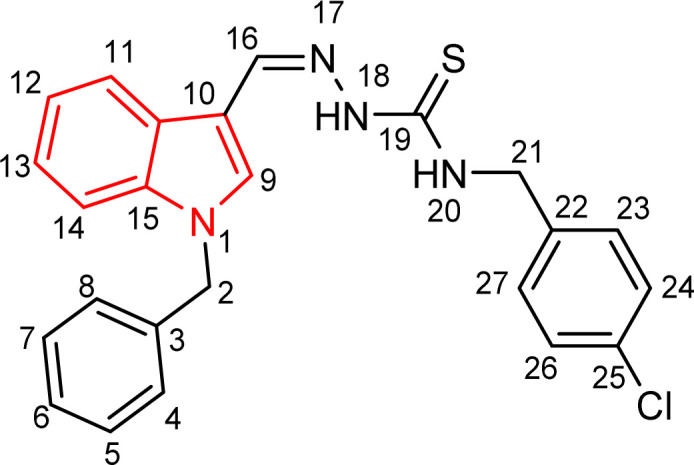



Color: white, yield: 77%, melting point: 225–227 °C. *δ*_H_ (600 MHz, DMSO-*d*_6_) 11.37 (1H, s, H-18), 8.50 (1H, t, *J* = 6.4 Hz, H-20), 8.36 (1H, s, H-9), 8.28 (1H, d, *J* = 7.9 Hz, Ar-H), 8.02 (1H, s, H-16), 7.51 (1H, d, *J* = 8.2 Hz, Ar-H), 7.41–7.38 (4H, m, Ar-H), 7.32 (2H, t, *J* = 7.6 Hz, Ar-H), 7.26 (3H, d, *J* = 7.5 Hz, Ar-H), 7.22 (1H, t, *J* = 7.7 Hz, Ar-H), 7.16 (1H, t, *J* = 7.5 Hz, Ar-H), 5.46 (2H, s, H-2), 4.89 (2H, d, *J* = 6.2 Hz, H-21); ^13^C NMR (151 MHz, DMSO) *δ* 177.05 (C-19), 140.95 (C-16), 139.35 (CH), 137.93 (C), 137.37 (C), 134.55 (CH), 131.62 (C), 129.44 (C-5, 7), 129.11 (CH), 128.59 (CH), 128.05 (CH), 127.64 (C-4, 8), 125.10 (C), 123.36 (CH), 122.91 (CH), 121.43 (CH), 111.24 (C), 111.10 (C), 49.85 (C-2), 46.48 (C-21); HPLC: CH_3_CN : H_2_O = 80 : 20; *t*_R_: 2.690 min, purity: 99.3%. TOF MS ES+ (*m*/*z*): [M + H]^+^, calcd: 433.1253, found 433.1253, anal. calcd for C_24_H_21_ClN_4_S; C, 60.93; H, 4.00; N, 12.36; found; C, 60.98; H, 4.12; N, 12.46.

##### 4.1.3.6 (*E*)-2-[(1-benzyl-1*H*-indol-3-yl)methylene]-*N*-(2,3-dichlorophenyl)hydrazinecarbo thioamide (5f)

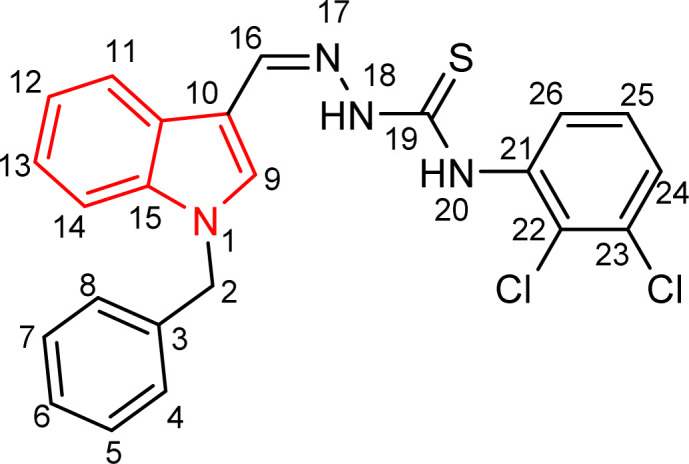



Color: greenish white, yield: 84%, melting point: 213–215 °C. *δ*_H_ (600 MHz, DMSO-*d*_6_) 11.92 (1H, s, H-18), 9.75 (1H, s, H-20), 8.45 (1H, s,H-9), 8.35 (1H, d, *J* = 7.9 Hz, Ar-H), 8.12–8.11 (1H, m, Ar-H), 8.01 (1H, s, H-16), 7.54 (2H, dd, *J* = 8.1, 1.6 Hz, Ar-H), 7.42 (1H, t, *J* = 8.1 Hz, Ar-H), 7.33 (2H, dd, *J* = 8.3, 6.8 Hz, Ar-H), 7.30–7.26 (3H, m, Ar-H), 7.25–7.24 (1H, m, Ar-H), 7.20–7.15 (1H, m, Ar-H), 5.48 (2H, s, H-2); ^13^C NMR (151 MHz, DMSO) *δ* 174.82 (C-19), 142.05 (C-16), 138.91 (CH), 137.84 (C), 137.45 (C), 135.53 (CH), 131.98 (C), 129.13 (C-5, 7), 128.09 (CH), 127.94 (C-4, 8), 127.69 (C), 127.67 (CH), 127.15 (C), 125.03 (C), 123.53 (CH), 122.81 (CH), 121.57 (CH), 111.31 (C), 110.96 (CH), 49.92 (C-2). HPLC: CH_3_CN : H_2_O = 80 : 20; *t*_R_: 4.467 min, purity: 97.7%. TOF MS ES+ (*m*/*z*): [M + H]^+^, calcd: 453.0707, found: 453.0704, anal. calcd for C_23_H_18_C_l2_N_4_S: C, 60.93; H, 4.00; N, 12.36; found: C, 60.99; H, 4.12; N, 12.42.

##### 4.1.3.7 (*E*)-2-[(1-benzyl-1*H*-indol-3-yl)methylene]-*N*-phenylhydrazinecarbothioamide (5g)

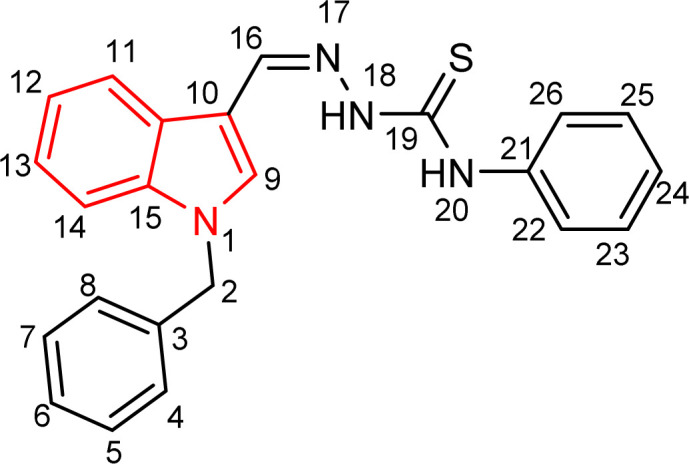



Color: white, yield: 87%, melting point: 203–205 °C. *δ*_H_ (600 MHz, DMSO-*d*_6_) 11.62 (1H, s, H-18), 9.64 (1H, s, H-20), 8.43 (1H, s, H-9), 8.27 (1H, d, *J* = 7.8 Hz, Ar-H), 8.11 (1H, s, H-16), 7.66 (2H, d, *J* = 7.9 Hz, Ar-H), 7.54 (1H, d, *J* = 8.1 Hz, Ar-H), 7.38 (2H, t, *J* = 7.7 Hz, Ar-H), 7.33 (2H, t, *J* = 7.5 Hz, Ar-H), 7.28–7.26 (3H, m, Ar-H), 7.24–7.21 (3H, m, Ar-H), 5.48 (2H, s, H-2); ^13^C NMR (151 MHz, DMSO) *δ* 175.02 (C-19), 141.15 (C-16), 139.77 (CH), 137.90 (C), 137.39 (C), 134.73 (CH), 129.13 (C-5, 7), 128.62 (CH), 128.08 (C), 127.65 (C-4, 8), 125.63 (CH), 125.45 (C), 125.23 (CH), 123.40 (CH), 122.66 (CH), 121.58 (CH), 111.21 (C), 111.14 (CH), 49.91 (C-2). HPLC: CH_3_CN : H_2_O = 80 : 20; *t*_R_: 2.284 min, purity: 99.9%. TOF MS ES+ (*m*/*z*): [M + H]^+^, calcd: 385.1486, found: 385.1887, anal. calcd for C_23_H_20_N_4_S: C, 71.85; H, 5.24; N, 14.57; found: C, 71.90; H, 5.29; N, 14.62.

##### 4.1.3.8 (*E*)-2-[(1-benzyl-1*H*-indol-3-yl)methylene]-*N*-(2,4-dimethylphenyl)hydrazinecarbo thioamide (5h)

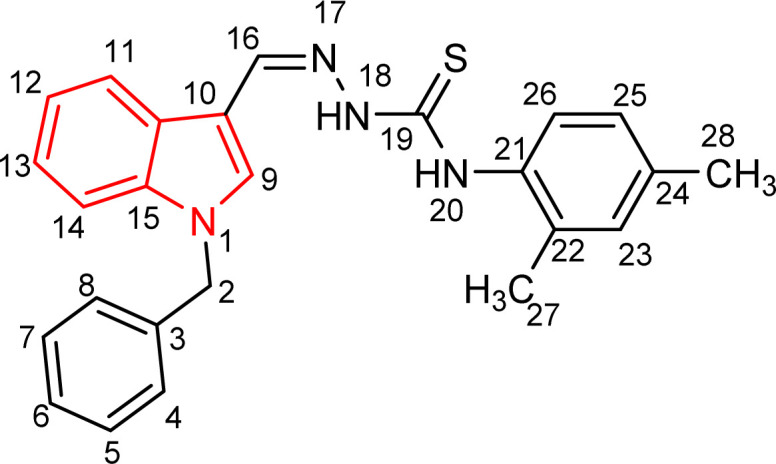



Color: white, yield: 86.5%, melting point: 180–182 °C. *δ*_H_ (600 MHz, DMSO-*d*_6_) 11.52 (1H, s, H-18), 9.34 (1H, s, H-20), 8.42 (1H, s, H-9), 8.37–8.29 (1H, m, Ar-H), 8.07 (1H, s, H-16), 7.57–7.48 (1H, m), 7.36–7.21 (3H, m), 7.27–7.20 (4H, m), 7.16–7.02 (3H, m), 5.47 (2H, s, H-2), 2.31 (3H, d, *J* = 3.6 Hz), 2.25 (3H, d, *J* = 3.6 Hz); ^13^C NMR (151 MHz, DMSO) *δ* 175.92 (C-19), 140.89 (CH), 137.94 (C-16), 137.38 (C), 136.11 (C), 135.82 (CH), 134.82 (C), 134.71 (CH), 131.04 (C), 129.11 (C-5, 7), 128.50 (CH), 128.06 (C), 127.64 (C-4, 8), 126.80 (CH), 125.16 (C), 123.37 (CH), 122.93 (CH), 121.43 (CH), 111.25 (CH), 111.12 (C), 49.88 (C-2), 21.08 (C-27), 18.27 (C-28). HPLC: CH_3_CN : H_2_O = 80 : 20; *t*_R_: 2.806 min, purity: 100.0%. TOF MS ES+ (*m*/*z*): [M + H]^+^, calcd: 413.1799, found: 413.1799, anal. calcd for C_25_H_24_N_4_S: C, 72.78; H, 5.86; N, 13.58; found: C, 72.82; H, 5.91; N, 13.63.

##### 4.1.3.9 (*E*)-*N*-benzyl-2-[(1-benzyl-1*H*-indol-3-yl)methylene]hydrazinecarbothioamide (5i)

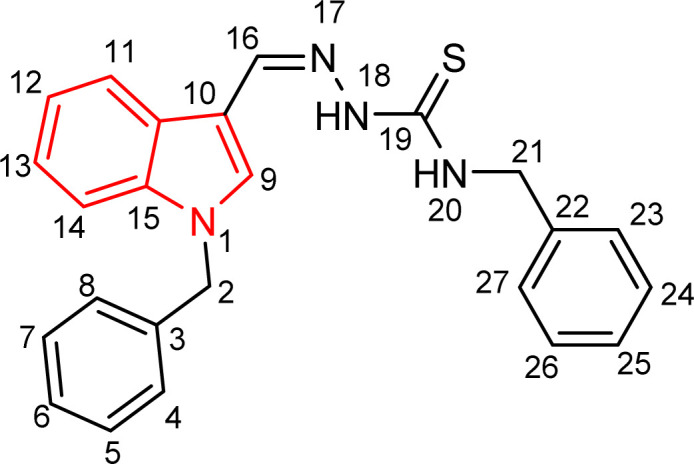



Color: white, yield: 66%, melting point: 193–195 °C. *δ*_H_ (600 MHz, DMSO-*d*_6_) 11.34 (1H, s, H-18), 8.43 (1H, t, *J* = 6.2 Hz, H-20), 8.35 (1H, s, H-9), 8.24 (1H, d, *J* = 7.9 Hz, Ar-H), 8.02 (1H, s, H-16), 7.51 (1H, d, *J* = 8.2 Hz, Ar-H), 7.39–7.30 (6H, m, Ar-H), 7.28–7.20 (4H, m, Ar-H), 7.21 (1H, t, *J* = 7.7 Hz, Ar-H), 7.13 (1H, t, *J* = 7.5 Hz, Ar-H), 5.46 (2H, s, H-2), 4.91 (2H, d, *J* = 6.1 Hz, H-21); ^13^C NMR (151 MHz, DMSO) *δ* 177.00 (C-19), 140.76 (C-16), 140.17 (CH), 137.94 (C), 137.36 (C), 134.51 (CH), 129.11 (C-5, 7), 128.68 (CH), 128.05 (CH), 127.64 (C-4, 8), 127.53 (CH), 127.16 (C), 125.09 (C), 123.34 (CH), 122.81 (CH), 121.40 (CH), 111.25 (C), 111.12 (CH), 49.84 (C-2), 47.11 (C-21). HPLC: CH_3_CN : H_2_O = 80 : 20; *t*_R_: 2.237 min, purity: 100.0%.TOF MS ES+ (*m*/*z*): [M + H]^+^, calcd: 399.1643, found: 399.1643, anal. calcd for C_24_H_22_N_4_S: C, 72.33; H, 5.56; N, 14.06; found: C, 72.39; H, 5.62; N, 14.10.

##### 4.1.3.10 (*E*)-2-[(1-benzyl-1*H*-indol-3-yl)methylene]-*N*-(4-bromophenyl)hydrazinecarbothioamide (5j)

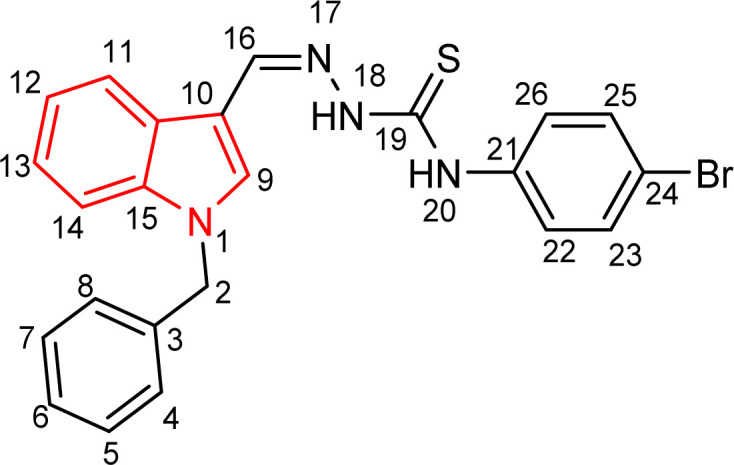



Color: greenish white, yield: 100%, melting point: 217–218 °C. *δ*_H_ (600 MHz, DMSO-*d*_6_) 11.69 (1H, s, H-18), 9.68 (1H, s, H-20), 8.43 (1H, s, H-9), 8.30–8.24 (1H, m), 8.10 (1H, s, H-16), 7.66–7.61 (2H, m), 7.58–7.51 (3H, m), 7.37–7.30 (2H, m), 7.27–7.25 (3H, m), 7.22 (1H, ddd, *J* = 8.3, 7.0, 1.3 Hz), 7.18 (1H, ddd, *J* = 8.1, 7.1, 1.1 Hz), 5.48 (2H, s, H-2); ^13^C NMR (151 MHz, DMSO) *δ* 174.92 (C-19), 141.49 (C-16), 139.26 (CH), 137.89 (C), 137.38 (C), 134.83 (CH), 131.36 (C), 129.13 (C-5, 7), 128.08 (CH), 127.69 (C-4, 8), 127.65 (CH), 125.22 (C), 123.41 (CH), 122.74 (CH), 121.56 (CH), 117.58 (CH), 111.19 (C), 111.08 (CH), 49.90 (C-2). HPLC: CH_3_CN : H_2_O = 80 : 20; *t*_R_: 3.159 min, purity: 97.3%. TOF MS ES+ (*m*/*z*): [M + H]^+^, calcd: 463.0592, found: 463.0561, anal. calcd for C_23_H_19_BrN_4_S: C, 59.61; H, 4.13; N, 12.09; found: C, 59.74; H, 4.25; N, 12.14.

##### 4.1.3.11 (*E*)-2-[(1-benzyl-1*H*-indol-3-yl)methylene]-*N*-(4-methylbenzyl)hydrazinecarbothioamide (5k)

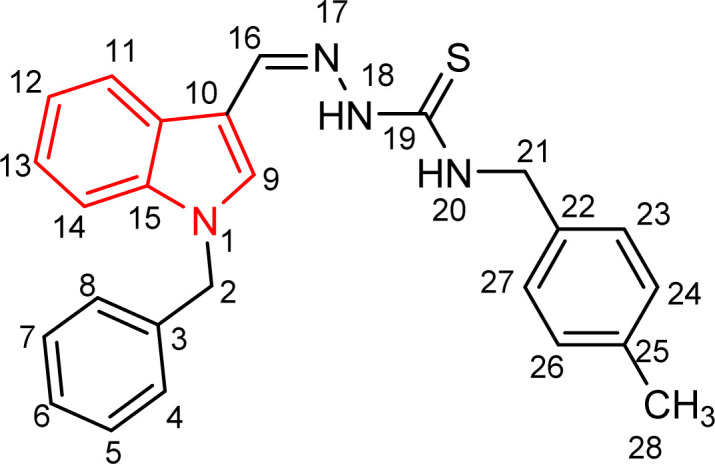



Color: white, yield: 87%, melting point: 223–225 °C. *δ*_H_ (600 MHz, DMSO-*d*_6_) 11.34 (1H, s, H-18), 8.38 (1H, t, *J* = 6.2 Hz, H-20), 8.36 (1H, s, H-9), 8.23 (1H, d, *J* = 7.9 Hz, Ar-H), 8.01 (1H, s, H-16), 7.51 (1H, d, *J* = 8.2 Hz, Ar-H), 7.34–7.30 (2H, m, Ar-H), 7.30–7.24 (5H, m, Ar-H), 7.22 (1H, t, *J* = 7.6 Hz, Ar-H), 7.18–7.11 (3H, m, Ar-H), 5.45 (2H, s, H-2), 4.87 (2H, d, *J* = 6.1 Hz, H-21), 2.28 (3H, s); ^13^C NMR (151 MHz, DMSO) *δ* 176.91 (C-19), 140.68 (C-16), 137.93 (CH), 137.36 (C), 137.07 (C), 136.22, 134.46 (CH), 129.24 (C-5, 7), 129.10, 128.05 (CH), 127.64 (C-4, 8), 127.57 (CH), 125.10 (C), 123.35 (CH), 122.78 (CH), 121.41 (CH), 111.27 (CH), 111.13 (C), 49.85 (C-2), 46.89 (C-21), 21.17 (C-28). HPLC: CH_3_CN : H_2_O = 80 : 20; *t*_R_: 2.615 min, purity: 100%. TOF MS ES+ (*m*/*z*): [M + H]^+^, calcd: 413.1799, found: 413.1798, anal. calcd for C_25_H_24_N_4_S: C, 72.78; H, 5.86; N, 13.58; found C, 72.82; H, 5.92; N, 13.62.

##### 4.1.3.12 (*E*)-2-[(1-benzyl-1*H*-indol-3-yl)methylene]-*N*-(3-methoxyphenyl)hydrazinecarbothioamide (5l)

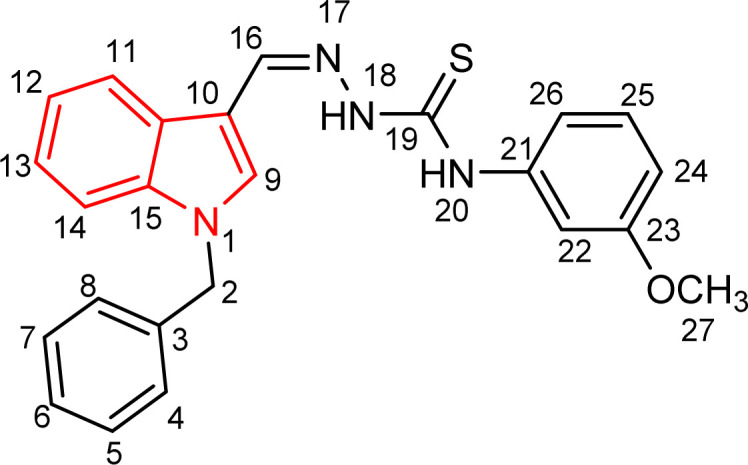



Color: off-white, yield: 75%, melting point: 187–189 °C. *δ*_H_ (600 MHz, DMSO-*d*_6_) 11.63 (1H, s, H-18), 9.60 (1H, s, H-20), 8.42 (1H, s, H-9), 8.23 (1H, dd, *J* = 7.6, 1.3 Hz, Ar-H), 8.11 (1H, s, H-16), 7.54 (1H, dt, *J* = 8.3, 1.0 Hz, Ar-H), 7.42 (1H, t, *J* = 2.2 Hz, Ar-H), 7.36–7.31 (2H, m, Ar-H), 7.30–7.25 (3H, m, Ar-H), 7.25–7.17 (3H, m, Ar-H), 6.78–6.76 (1H, m, Ar-H), 5.48 (2H, s, H-2), 3.78 (3H, s, OCH_3_); ^13^C NMR (151 MHz, DMSO) *δ* 174.71, 159.55, 141.14, 140.84, 137.90, 137.38, 134.74, 129.36, 129.13, 128.08, 127.64, 125.23, 123.40, 122.55, 121.59, 117.44, 111.24, 111.09, 110.93, 110.82, 55.61, 49.90. HPLC: CH_3_CN : H_2_O = 80 : 20; *t*_R_: 2.293 min, purity: 99.8%. TOF MS ES+ (*m*/*z*): [M + H]^+^, calcd: 415.159, found: 415.1590, anal. calcd for C_24_H_22_N_4_OS: C, 69.54; H, 5.35; N, 13.52; found: C, 69.59; H, 5.42; N, 13.58.

##### 4.1.3.13 (*E*)-2-[(1-benzyl-1*H*-indol-3-yl)methylene]-*N*-(3,5-dimethylphenyl)hydrazine carbo thioamide (5m)

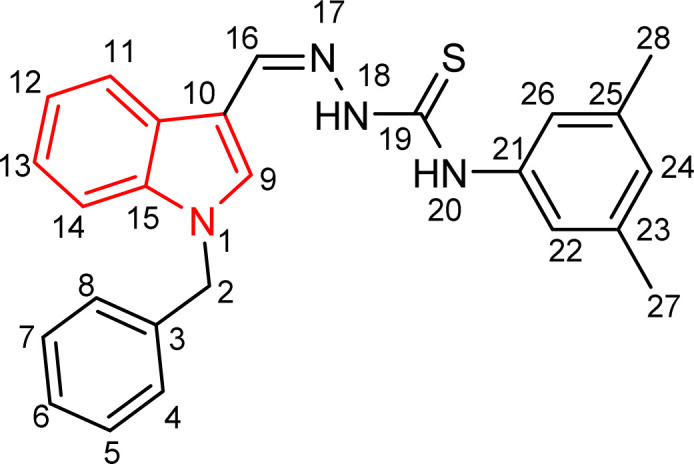



Color: white, yield: 59%, melting point: 221–223 °C. *δ*_H_ (600 MHz, DMSO-*d*_6_) 11.46 (1H, s, H-18), 9.29 (1H, s, H-20), 8.43 (1H, s, Ar-H), 8.41 (1H, s, Ar-H), 8.06 (1H, s, H-16), 7.50 (1H, d, *J* = 8.2 Hz, Ar-H), 7.34–7.31 (2H, m, Ar-H), 7.30–7.24 (3H, m, Ar-H), 7.24–7.19 (1H, m, Ar-H), 7.17–7.10 (4H, m, Ar-H), 5.47 (2H, s, H-2, Ar-H), 2.23 (6H, s, H-27, 28, Ar-H); ^13^C NMR (151 MHz, DMSO) *δ* 176.14 (C-19), 140.80 (C-16), 138.12 (CH), 137.99 (C), 137.36 (C), 137.17 (CH), 134.62 (C), 129.10 (C-5, 7), 128.04 (CH), 128.01 (C), 127.58 (C-4, 8), 127.24 (CH), 125.18 (C), 123.33 (CH), 121.39 (CH), 111.37 (CH), 110.97 (C), 49.86 (C-2), 18.67 (–CH_3_). HPLC: CH_3_CN : H_2_O = 80 : 20; *t*_R_: 2.441 min, purity: 99.8%. TOF MS ES+ (*m*/*z*): [M + H]^+^, calcd: 413.1799, found: 413.1799, anal. calcd for C_25_H_24_N_4_S: C, 72.78; H, 5.86; N, 13.58; found: C, 72.83; H, 5.91; N, 13.63.

##### 4.1.3.14 (*E*)-2-[(1-benzyl-1*H*-indol-3-yl)methylene]-*N*-(*p*-tolyl)hydrazinecarbothioamide (5n)

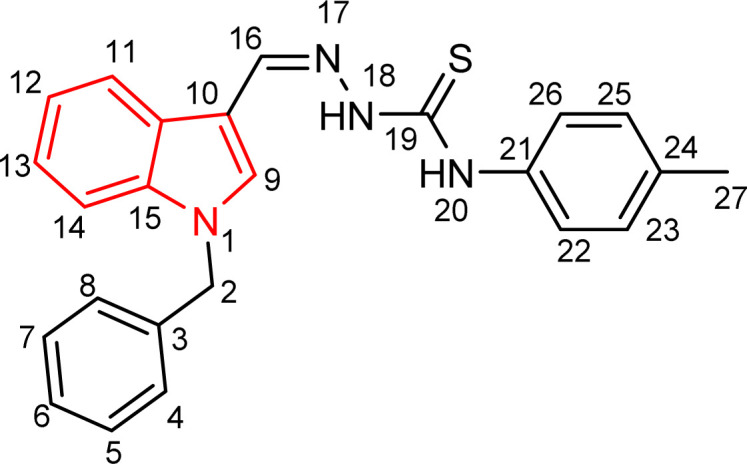



Color: white, yield: 72%, melting point: 221–223 °C. *δ*_H_ (600 MHz, DMSO-*d*_6_) 11.57 (1H, s, H-18), 9.55 (1H, s, H-20), 8.43 (1H, s, H-9), 8.26 (1H, dd, *J* = 7.7, 1.3 Hz, Ar-H), 8.10 (1H, s, H-16), 7.53 (1H, dt, *J* = 8.2, 0.9 Hz, Ar-H), 7.52–7.48 (2H, m, Ar-H), 7.35–7.31 (2H, m, Ar-H), 7.29–7.25 (3H, m, Ar-H), 7.23 (1H, ddd, *J* = 8.3, 7.0, 1.3 Hz, Ar-H), 7.21–7.16 (3H, m, Ar-H), 5.47 (2H, s, H-2), 2.32 (3H, s, CH_3_); ^13^C NMR (151 MHz, DMSO) *δ* 175.13 (C-19), 140.98 (C-16), 137.91 (CH), 137.38 (C), 137.20 (C), 134.64 (CH), 129.12 (C), 129.07 (C-5, 7), 128.07 (CH), 127.65 (C-4, 8), 125.70 (CH), 125.24 (C), 123.37 (CH), 122.62 (CH), 121.55 (CH), 111.20 (CH), 111.16 (C), 49.90 (C-2), 21.06 (C-27). HPLC: CH_3_CN : H_2_O = 80 : 20; *t*_R_: 2.652 min, purity: 99.1%. TOF MS ES+ (*m*/*z*): [M + H]^+^, calcd: 399.1643, found: 399.1643, anal. calcd for C_24_H_22_N_4_S: C, 72.33; H, 5.56; N, 14.06; found: C, 72.39; H, 5.62; N, 14.12.

##### 4.1.3.15 (*E*)-2-[(1-benzyl-1*H*-indol-3-yl)methylene]-*N*-(3-nitrophenyl)hydrazinecarbothioamide (5o)

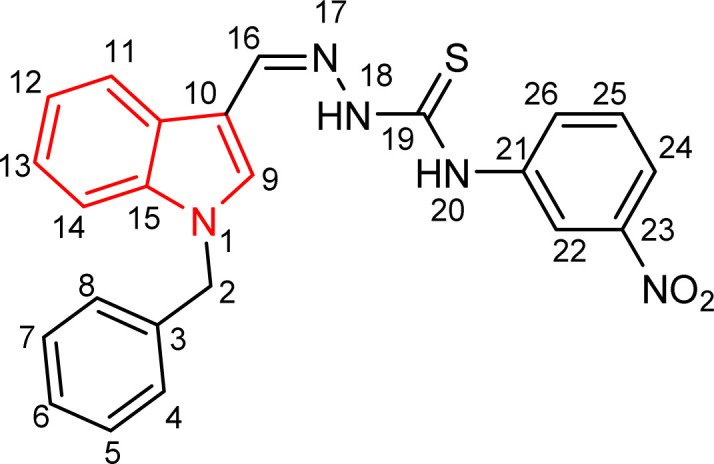



Color: dark yellow, yield: 89%, melting point: 208–210 °C. *δ*_H_ (600 MHz, DMSO-*d*_6_) 11.87 (1H, s, H-18), 9.99 (1H, s, H-20), 8.75 (1H, t, *J* = 2.2 Hz, Ar-H), 8.47 (1H, s, H-9), 8.32 (1H, d, *J* = 7.8 Hz, Ar-H), 8.12 (1H, s, H-16), 8.04 (1H, ddd, *J* = 8.2, 2.3, 1.0 Hz, Ar-H), 7.66 (1H, t, *J* = 8.1 Hz, Ar-H), 7.57–7.51 (1H, m, Ar-H), 7.36–7.31 (2H, m, Ar-H), 7.28–7.26 (3H, m, Ar-H), 7.24 (1H, ddd, *J* = 8.2, 7.0, 1.3 Hz, Ar-H), 7.20 (1H, td, *J* = 7.5, 7.1, 1.1 Hz, Ar-H), 5.49 (2H, s, H-2); ^13^C NMR (151 MHz, DMSO) *δ* 174.95 (C-19), 147.77 (C-16), 142.09 (C), 141.17 (CH), 137.87 (C), 137.41 (C), 135.03 (CH), 132.03 (C), 129.69 (C-5, 7), 129.13 (CH), 128.09 (CH), 127.65 (C-4, 8), 125.24 (C), 123.46 (CH), 122.85 (CH), 121.59 (CH), 119.77 (CH), 111.20 (CH), 111.04 (C), 49.93 (C-2). HPLC: CH_3_CN : H_2_O = 80 : 20; *t*_R_: 2.205 min, purity: 99.8%. TOF MS ES+ (*m*/*z*): [M + H]^+^, calcd: 430.1337, found: 430.1337, anal. calcd for C_23_H_19_N_5_O_2_S: C, 64.32; H, 4.46; N, 16.31; O, 7.45; S, 7.47; found: C, 64.38; H, 4.52; N, 16.37.

##### 4.1.3.16 (*E*)-2-[(1-benzyl-1*H*-indol-3-yl)methylene]-*N*-methylhydrazinecarbothioamide (5p)

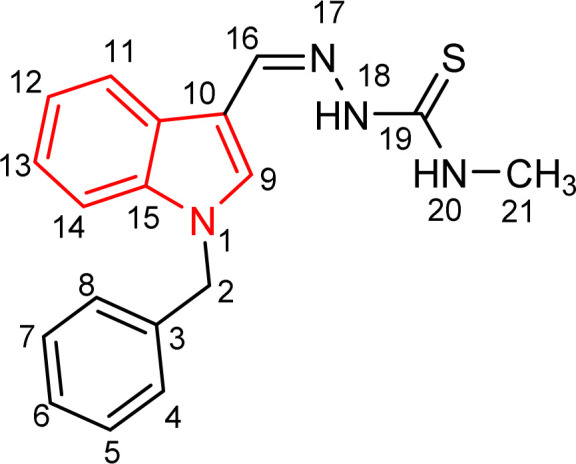



Color: white, yield: 72%, melting point: 220–221 °C. *δ*_H_ (600 MHz, DMSO-*d*_6_) 11.19 (1H, s, H-18), 8.32 (1H, S, H-9), 7.99 (1H, s, H-16), 7.94 (1H, q, *J* = 4.5 Hz, H-20), 7.51 (1H, d, *J* = 8.1 Hz, Ar-H), 7.33–7.31 (2H, m, Ar-H), 7.27–7.26 (3H, m, Ar-H), 7.25–7.21 (1H, m, Ar-H), 7.18 (1H, t, *J* = 7.5 Hz, Ar-H), 5.45 (2H, s, H-2), 3.09 (3H, d, *J* = 4.5 Hz, H-21); ^13^C NMR (151 MHz, DMSO) *δ* 177.27 (C-19), 140.35 (C-16), 137.94 (C), 137.35 (C), 134.31 (CH), 129.11 (C-5, 7), 128.05 (CH), 127.68 (C-4, 8), 125.08 (C), 123.33 (CH), 123.04 (CH), 121.38 (CH), 111.35 (CH), 111.04 (C), 49.84 (C-2), 31.52 (C-21). HPLC: CH_3_CN : H_2_O = 80 : 20; *t*_R_: 1.592 min, purity: 100.0%. TOF MS ES+ (*m*/*z*): [M + H]^+^, calcd: 323.1330, found: 323.1330, anal. calcd for C_18_H_18_N_4_S: C, 67.05; H, 5.63; N, 17.38; S, 9.94; found: C, 67.12; H, 5.69; N, 17.45.

##### 4.1.3.17 (*E*)-2-[(1-benzyl-1*H*-indol-3-yl)methylene]-*N*-(4-nitrophenyl)hydrazinecarbothioamide (5q)

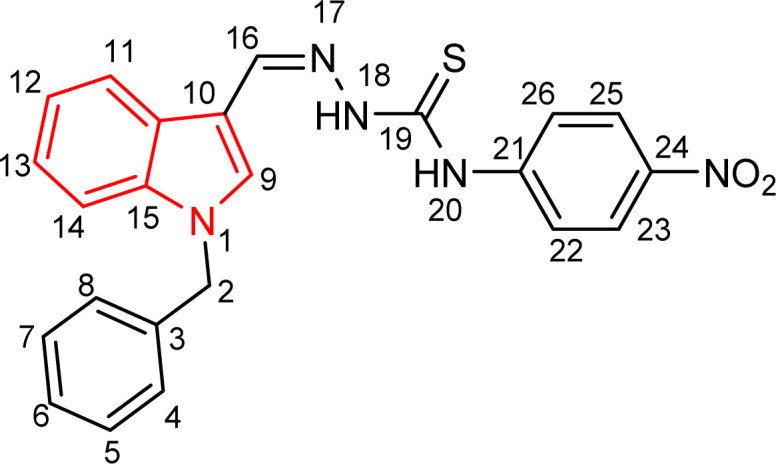



Color: dark yellow, yield: 67%, melting point: 223–225 °C. *δ*_H_ (600 MHz, DMSO-*d*_6_) 11.99 (1H, s, H-18), 10.10 (1H, s, H-20), 8.47 (1H, s, H-9), 8.29–8.21 (3H, m), 8.14 (1H, s, H-16), 8.11 (2H, d, *J* = 9.0 Hz), 7.54 (1H, d, *J* = 8.1 Hz), 7.33 (2H, t, *J* = 7.5 Hz), 7.29–7.26 (3H, m), 7.26–7.22 (1H, m), 7.22–7.18 (1H, m), 5.49 (2H, s, H-2); ^13^C NMR (151 MHz, DMSO) *δ* 174.19 (C-19), 146.25 (C-16), 143.49 (C), 142.27 (CH), 137.84 (C), 137.43 (C), 135.23 (CH), 129.13 (C-5, 7), 128.10 (CH), 127.65 (C-4, 8), 125.23 (C), 124.39 (C), 123.86 (CH), 123.49 (CH), 122.68 (CH), 121.67 (CH), 111.28 (CH), 110.96 (C), 49.94 (C-2). HPLC: CH_3_CN : H_2_O = 80 : 20; *t*_R_: 2.363 min, purity: 88.5%. TOF MS ES+ (*m*/*z*): [M + H]^+^, calcd: 430.1337, found: 430.1338, anal. calcd for C_23_H_19_N_5_O_2_S: C, 64.32; H, 4.46; N, 16.31; found: C, 64.39; H, 4.53; N, 16.36.

##### 4.1.3.18 (*E*)-2-[(1-benzyl-1*H*-indol-3-yl)methylene]-*N*-(naphthalen-1-yl)hydrazinecarbothioamide (5r)

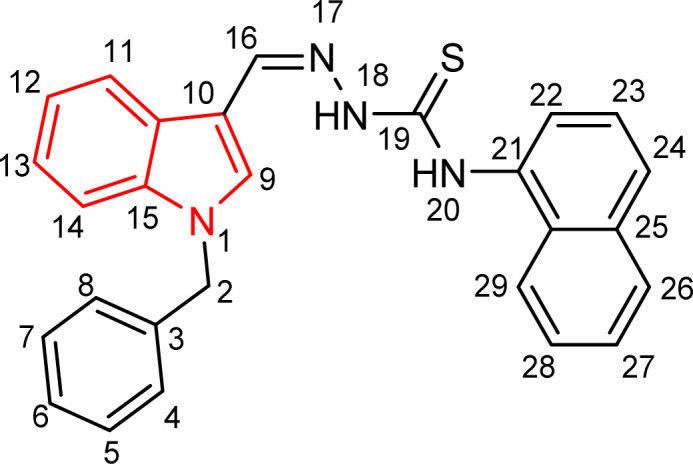



Color: white, yield: 66%, melting point: 199–201 °C. *δ*_H_ (600 MHz, DMSO-*d*_6_) 11.69 (1H, s, H-18), 9.88 (1H, s, H-20), 8.49 (1H, s, H-9), 8.39 (1H, d, *J* = 8.0 Hz), 8.12 (1H, s, H-16), 8.02–7.97 (1H, m), 7.96–7.93 (1H, m), 7.90 (1H, d, *J* = 8.2 Hz), 7.66 (1H, dt, *J* = 7.3, 1.0 Hz), 7.60–7.49 (4H, m), 7.35–7.32 (2H, m), 7.28–7.26 (3H, m), 7.21 (1H, ddd, *J* = 8.2, 7.0, 1.2 Hz), 7.11 (1H, ddd, *J* = 8.0, 7.0, 1.0 Hz), 5.48 (2H, s, H-2); ^13^C NMR (151 MHz, DMSO) *δ* 176.81 (C-19), 141.24 (C-16), 137.95 (CH), 137.40 (C), 136.23 (C), 134.86 (CH), 134.20 (C), 130.94 (CH), 129.12 (C-5, 7), 128.56 (CH), 128.07 (CH), 127.65 (C-4, 8), 127.07 (CH), 126.58 (CH), 126.52 (CH), 126.47 (CH), 125.91 (C), 125.19 (C), 123.49 (C), 123.37 (CH), 123.04 (CH), 121.42 (CH), 111.26 (CH), 111.11 (C), 49.89 (C-2). HPLC: CH_3_CN : H_2_O = 80 : 20; *t*_R_: 2.554 min, purity: 100.0%. TOF MS ES+ (*m*/*z*): [M + H]^+^, calcd: 435.1643, found: 435.1638, anal. calcd for C_27_H_22_N_4_S: C, 74.63; H, 5.10; N, 12.89; found: C, 74.69; H, 5.18; N, 12.95.

### 4.2. Tyrosinase inhibition assay

Using a spectrophotometer microplate reader, the 96-well plate tyrosinase inhibition experiment was performed by Masamoto *et al.*'s protocol.^65,66^ First, 10 μL of the test compounds and the positive control, “kojic acid” were added to the 96-well microplate. Then, add 60 μL of phosphate buffer and 10 μL of mushroom tyrosinase (30 U mL^−1^ in phosphate buffer) to the reaction mixture incubated for 10 min at 25 °C. Following incubation, 20 μL of substrate l-DOPA (0.5 mM) in phosphate buffer was added. The end product dopachrome formation was measured at 480 nm for continues 10 min with one min interval time using 96-well ELISA plate reader (Bio-Rad xMARK microplate spectrophotometer). All of the experiments were carried out in triplicate to account for potential mistakes, and the mean findings were reported in SEM. All of the *in vitro* experiment results, including % inhibition and IC_50_ values, were examined using EZ-Fit, an enzyme kinetics tool that fits curves (Perrella Scientific Inc., Amherst, USA).

The *in vitro* mechanistic analysis was carried out under comparable experimental settings to get insight into the mechanism of action of the inhibitors that were found. Four distinct doses of the substrate l-Dopa 0.1, 0.2, 0.4, and 0.8 mM were used in the kinetic analysis.

#### 4.2.1. Statistical analysis

The tools utilized to evaluate the obtained biological activity values were Excel and the SoftMax Pro.

The % inhibition was computed using the formula provided below.1
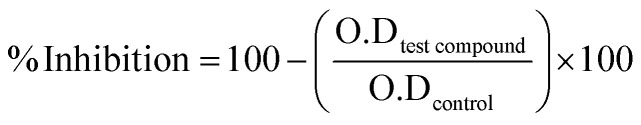


EZ-FIT (Perrella Scientific, Inc., USA) was used to calculate each tested compound's IC_50_ value. All the experiments were performed in triplicate to reduce predicted errors, and differences in the outcomes are expressed as Standard Error of Mean values (SEM).2
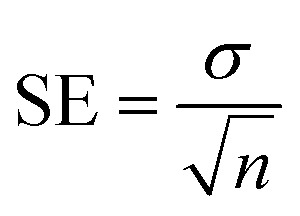


### 4.3. Kinetic analysis

The *in vitro* mechanistic study was carried out by following a similar inhibitory procedure with a similar experimental temperature and time period. The addition of four different concentrations of the substrate l-Dopa including 0.1, 0.2, 0.4, and 0.8 mM was used in the kinetic analysis.

### 4.4. Molecular docking

To explore the *in silico* tyrosinase inhibition activity, the X-ray crystal structure of tyrosinase inhibitor (PDB ID: 2Y9W) which is the crystal structure of PPO3, a tyrosinase from *Agaricus bisporus*, in deoxy-form that contains additional unknown lectin-like subunit with a resolution of 2.30 Å, was retrieved from RCSB protein data bank^67^ in PDB format. The protein was processed for molecular docking with the aid of a protein preparation wizard from the Schrodinger suite (http://www.schrodinger.com). The processing includes the stepwise rectification of missing side chains or loops, and then allocation of bond orders. The protonation states for each of the hetero-atoms were detected based on the number of hydrogen bonds, as well as the score Epik penalty. Consequently, the protein structure was refined. In addition, the partial atomic charges were computed precisely by using the OPLS4 force field. For a cluster of hydrogen-bonded species, an interactive optimization was executed, and finally, the optimized structure was saved as.mae (Maestro) file format within the specified already chosen working directory.^68^ The two-dimensional (2D) structures of the investigated compounds (ligands) were sketched by ChemDraw 20.1.1, followed by energy minimization *via* Chem3D 20.1.1, and then saved in.sdf file format. Furthermore, the LigPrep module of Schrödinger Release 2016-4 was incorporated to generate, and optimize their three-dimensional (3D) structures, employing the OPLS4 force field.^69^ The receptor grid generation tool within the Schrödinger suite was incorporated to make a grid box, the dimensions of which were accustomed to cover the co-crystallized ligand within the binding pocket of the chosen target protein. The docking process was conducted by the Glide module in standard precision (SP) mode.^70^ The glide and Emodel scores were tabulated for each ligand's optimal conformational pose and were compared with the scores of the corresponding co-crystallized ligand and the standard drug.

### 4.5. Validation of docking protocol

In our experiment, each ligand from Table 2 was docked *via* the Maestro glide software with the co-crystallized ligand of the target protein (reference). The hit compound 5k (according to the scoring function energies) was chosen for further structural analysis by calculating the root mean square deviation (RMSD) of each pose against its conformation in the crystal structure.^71^ Here, three different RMSD classifications for docking solutions: (i) good solutions when RMSD ≤ 2.0 Å, (ii) acceptable solutions when RMSD is between 2.0 and 3.0 Å, and (iii) bad solutions when RMSD ≥ 3.0 Å.

### 4.6. ADMET analysis

ADMET study is designed to predict ADMET properties (absorption, distribution, metabolism, excretion, and toxicity) of investigated compounds *via* the SwissADME web server.^72^ The compounds were evaluated by ADMET analysis based on established rules established by Lipinski *et al.*,^73^ Muegge *et al.*,^74^ Ghose *et al.*,^75^ Egan *et al.*,^76^ and Veber *et al.*^77^ In addition, a range of a variety of other characteristics was also analyzed, such as the number of hydrogen bond donors, hydrogen bond acceptors, rotatable bonds, total polar surface area, and synthetic accessibility to determine the drug likeliness of synthesized compounds.

## Data availability

The data supporting this article have been included as part of the ESI.†

## Conflicts of interest

The authors have declared no conflict of interest.

## Supplementary Material

RA-014-D4RA05015K-s001

RA-014-D4RA05015K-s002
