# Supplementary material for: Design, synthesis, and in vitro and in silico study of 1-benzyl-indole hybrid thiosemicarbazones as competitive tyrosinase inhibitors
Source: RSC Adv. 2024 Sep 6;14(39):28524–42. doi: 10.1039/d4ra05015k (PMC11378339; doi:10.1039/d4ra05015k)

Supplementary Information (SI) for RSC Advances.
This journal is © The Royal Society of Chemistry 2024

Design, synthesis, *in vitro,* and *in silico* study of *1*-benzyl-indole hybrid thiosemicarbazones as competitive tyrosinase inhibitors

Content

Dose Curve response for compounds 5a-5r 4

Figure S1. Dose curve response for compounds 5a- 5r against tyrosinase. 12

Glide molecular docking studies 12

Table S1. Glide Score, H-Bonding Interactions with Distances in Å, Polar, and Hydrophobic Interacting Residues for Investigated Ligands with target protein possessing tyrosinase activity (PDB ID: 2Y9W). 12

Figure S2. 3D and 2D representation of interaction with tyrosinase target protein (PDB ID: 2Y9W). 13

Table S2. Docking Score, glide Score, and glide emodel values of all investigated ligands with target protein possessing tyrosinase activity (PDB ID: 2Y9W). 13

Figure S3. The boiled-egg plot (plot of WLOGP against TPSA) of all investigated compounds from SwissADME web tool..20

1H NMR and 13C NMR Spectra of 5a..20

1H NMR and 13C NMR Spectra of 5b 22

1H NMR and 13C NMR Spectra of 5c..23

1H NMR and 13C NMR Spectra of 5d 24

1H NMR and 13C NMR Spectra of 5e..25

1H NMR and 13C NMR Spectra of 5f 26

H NMR and 13C NMR Spectra of 5g 27

H NMR and 13C NMR Spectra of 5h 28

1H NMR and 13C NMR Spectra of 5i 29

1H NMR and 13C NMR Spectra of 5j 30

^{1}H NMR and ^{13}C NMR Spectra of 5k 31

1H NMR and 13C NMR Spectra of 5l 32

1H NMR and 13C NMR Spectra of 5m 33

1H NMR and 13C NMR Spectra of 5n 34

^{1}H NMR and ^{13}C NMR Spectra of 5o 35

1H NMR and 13C NMR Spectra of 5p 36

1H NMR and 13C NMR Spectra of 5q 37

1H NMR and 13C NMR Spectra of 5r 38
HPLC chromatogram of 5a 39
HPLC chromatogram of 5b 39
HPLC chromatogram of 5c 40
HPLC chromatogram of 5d 40
HPLC chromatogram of 5e 40
HPLC chromatogram of 5f 41
HPLC chromatogram of 5g 41
HPLC chromatogram of 5h 42
HPLC chromatogram of 5i 42
HPLC chromatogram of 5j 43
HPLC chromatogram of 5k 44
HPLC chromatogram of 5l 44
HPLC chromatogram of 5m 44
HPLC chromatogram of 5n 45
HPLC chromatogram of 5o 45
HPLC chromatogram of 5p 46
HPLC chromatogram of 5q 46
HPLC chromatogram of 5r 47
Mass spectrum of 5a 48
Mass spectrum of 5b 48
Mass spectrum of 5c 49
Mass spectrum of 5d 49
Mass spectrum of 5e 50
Mass spectrum of 5f 50
Mass spectrum of 5g 51
Mass spectrum of 5h 51
Mass spectrum of 5i 52
Mass spectrum of 5j 52
Mass spectrum of 5k 53
Mass spectrum of 5l 53
Mass spectrum of 5m 54

Mass spectrum of 5n 54
Mass spectrum of 5o 55
Mass spectrum of 5p 55
Mass spectrum of 5q 56
Mass spectrum of 5r...56

Dose Curve response for compounds 5a-5r

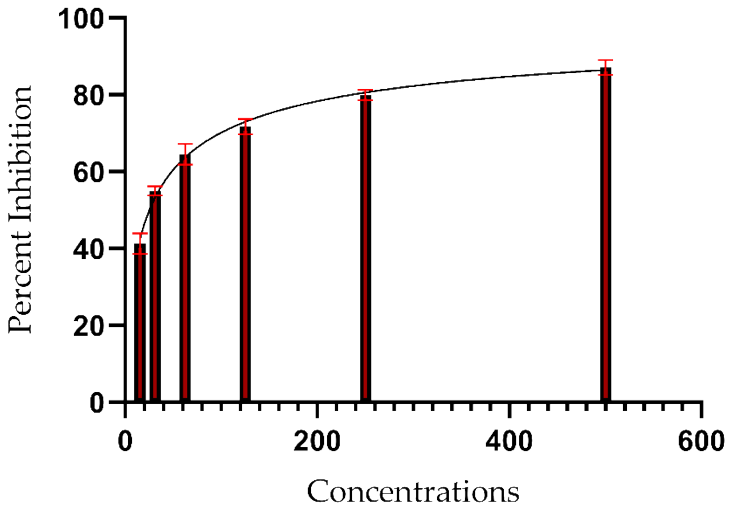

Compound **5a**

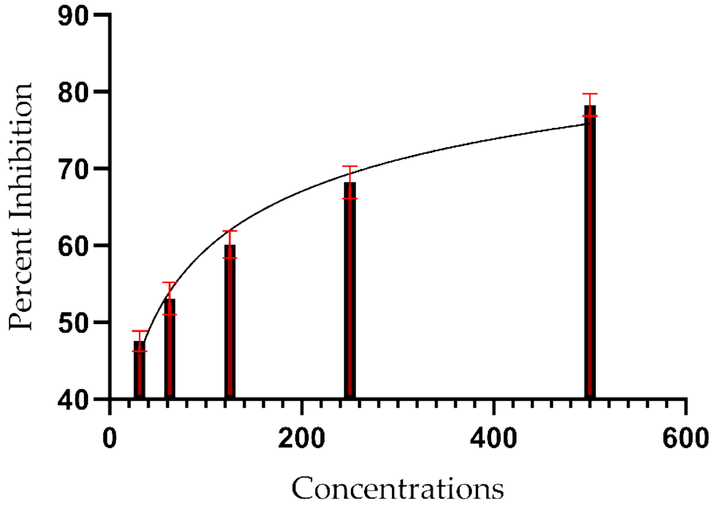

Compound **5b**

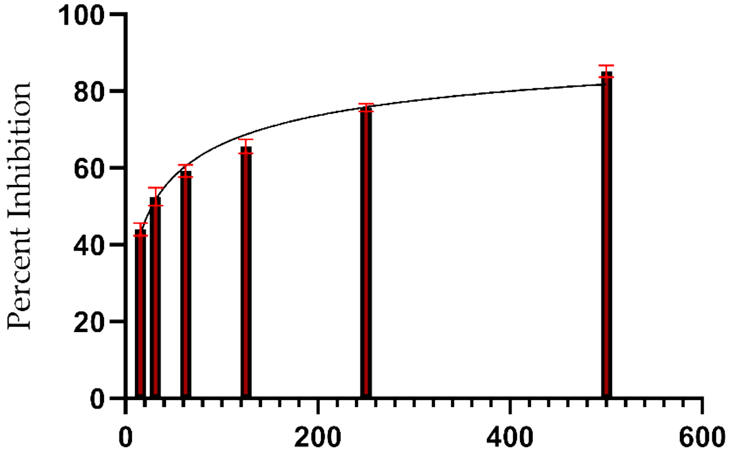

Concentrations
Compound **5c**

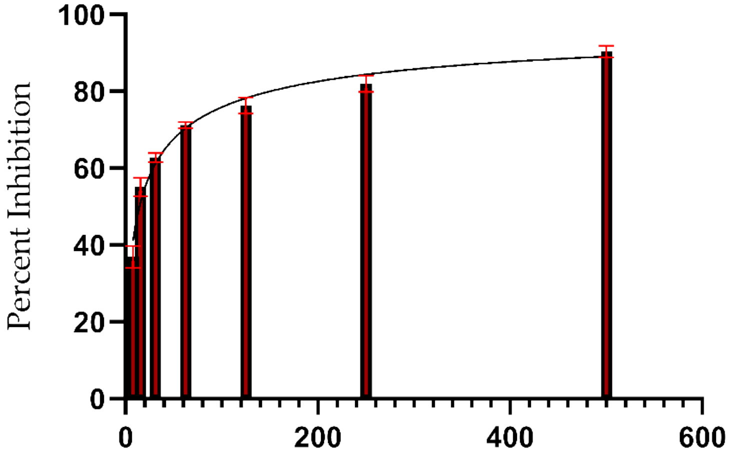

Concentrations
Compound **5d**

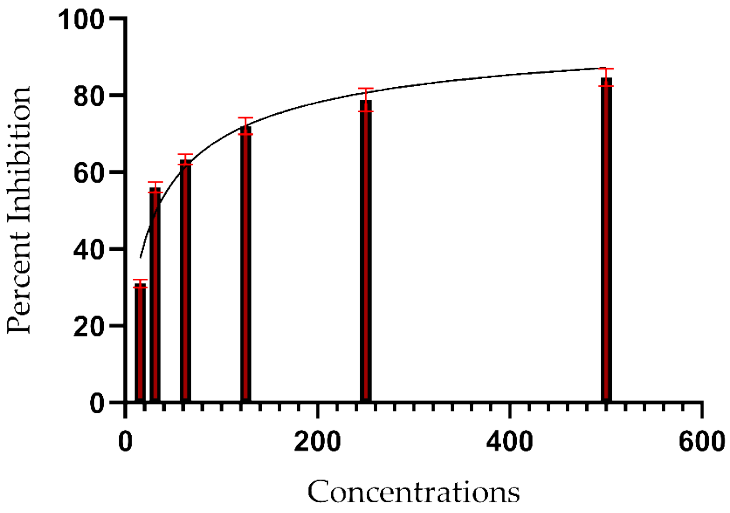

Compound **5e**

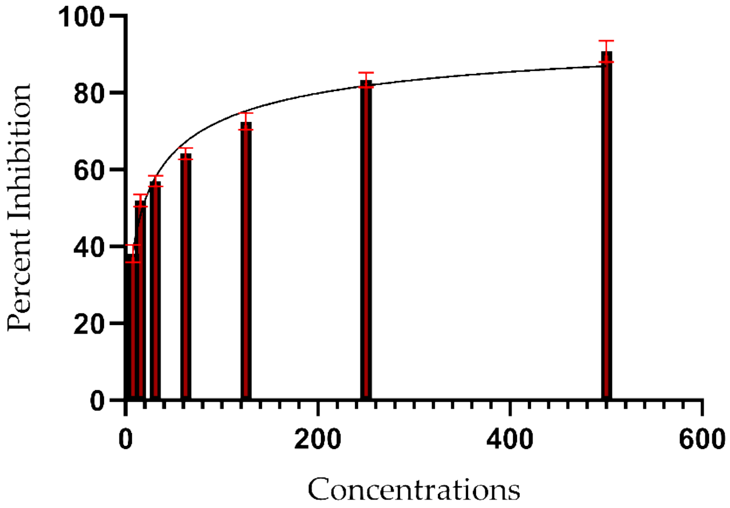

Compound **5f**

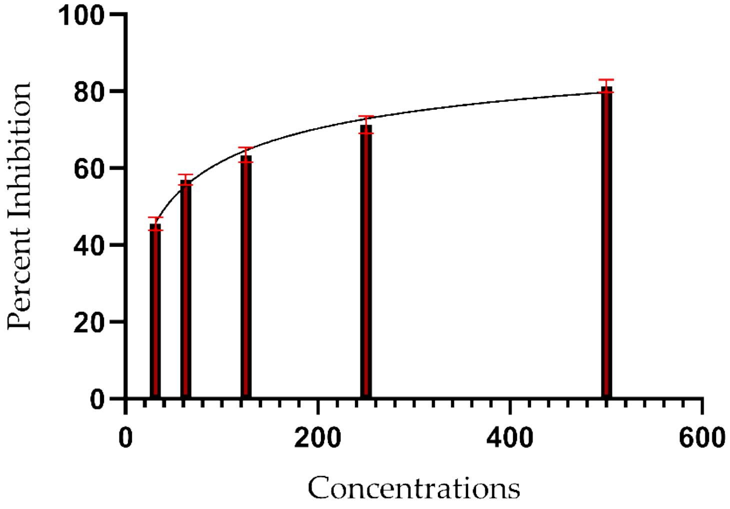

Compound **5g**

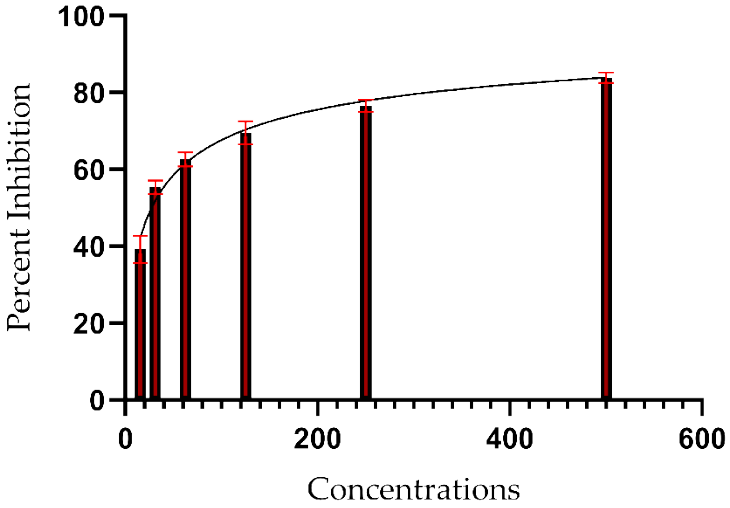

Compound **5h**

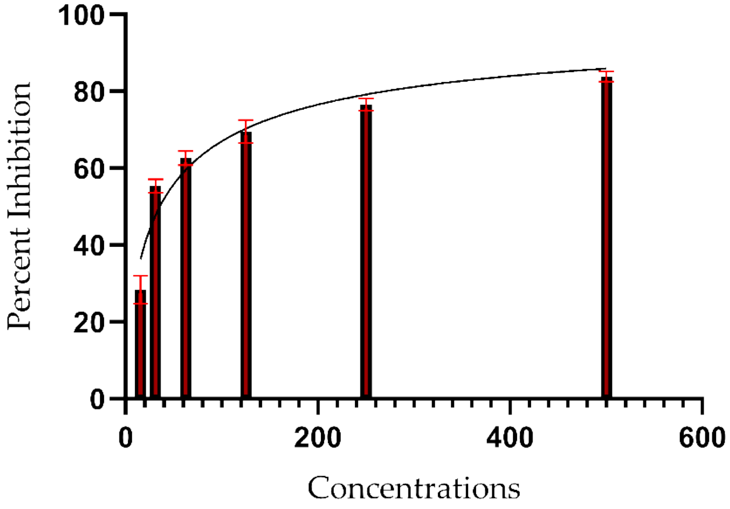

Compound **5i**

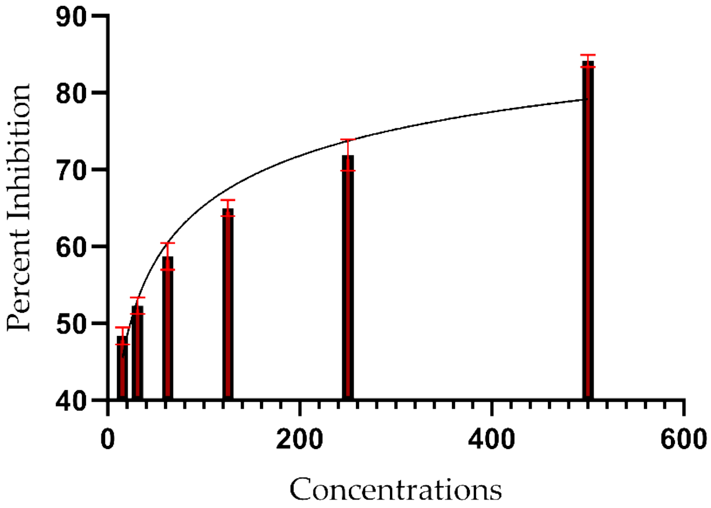

Compound **5j**

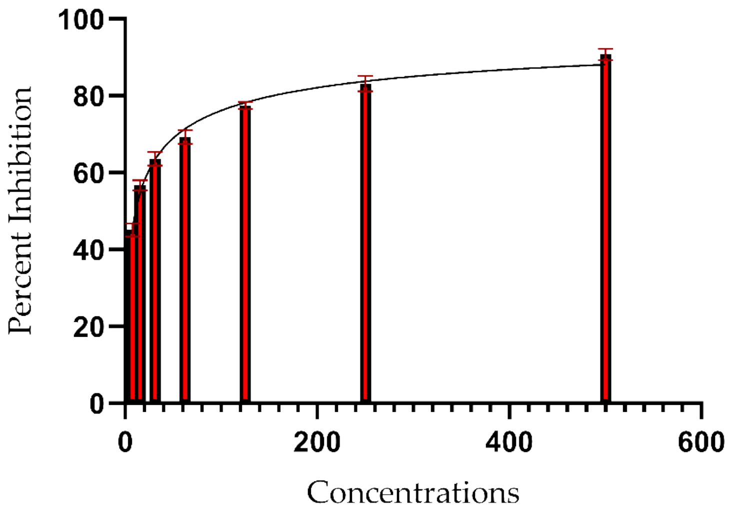

Compound **5k**

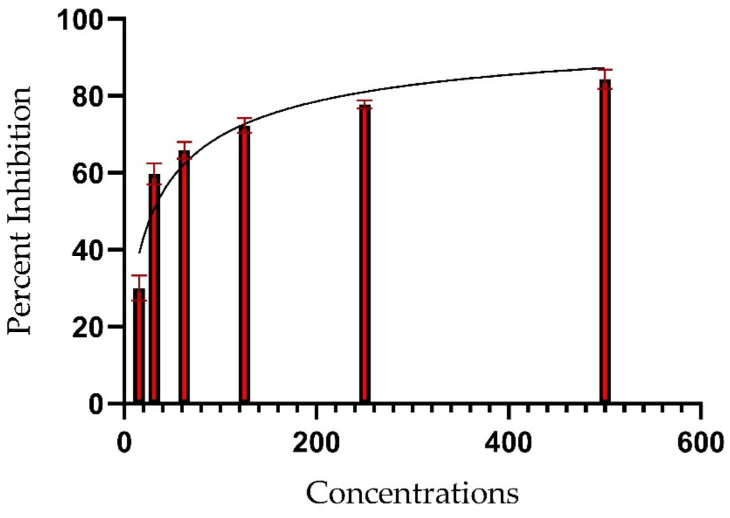

Compound **5l**

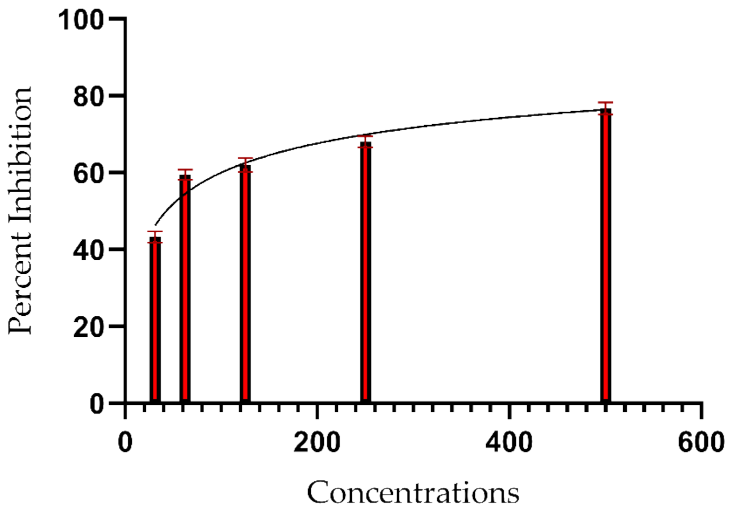

Compound **5m**

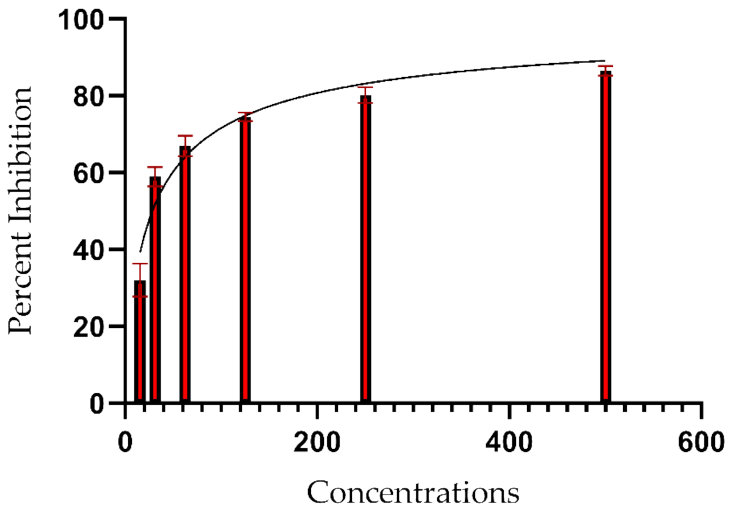

Compound **5n**

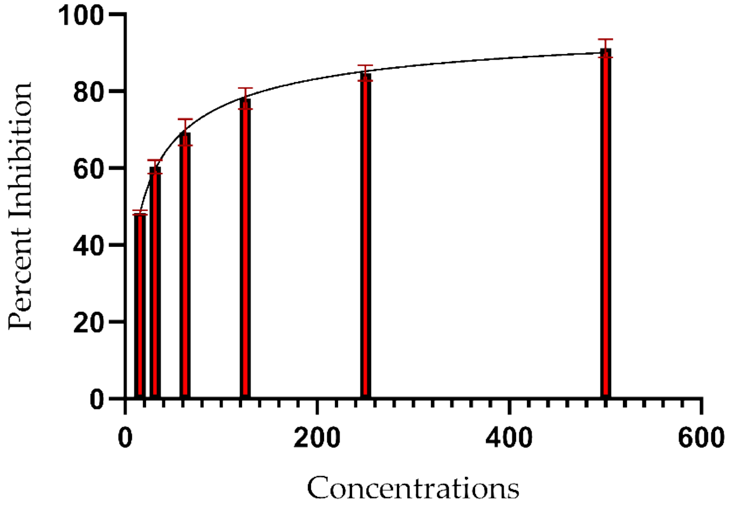

Compound **5o**

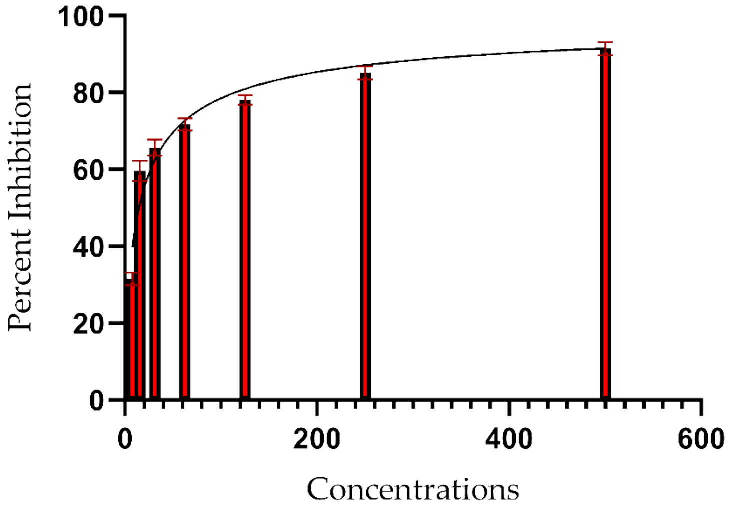

Compound **5q**

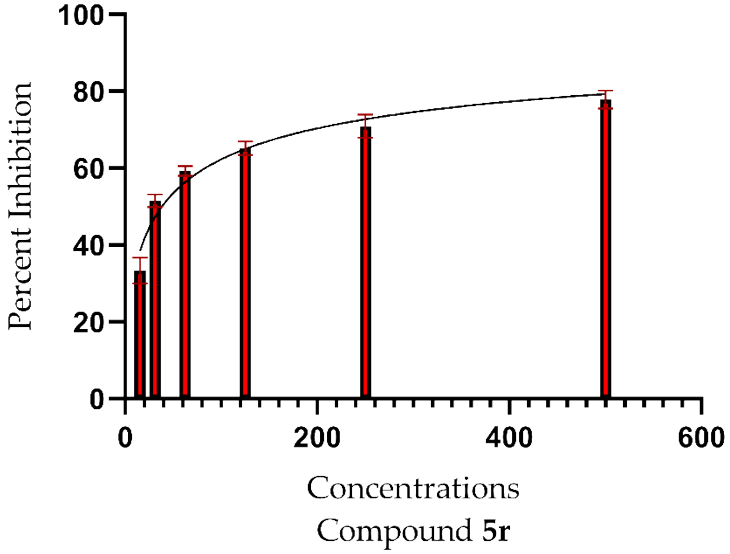

Figure S1. Dose curve response for compounds 5a- 5r against tyrosinase.

Glide molecular docking studies

The compounds 5c and 5b demonstrated strong binding interactions with tyrosinase target protein with gScores values even much better than the co-crystallized ligand and the kojic acid. Table S1 details the gScore, emodel, H-bonding, and hydrophobic interacting residues for the ligands 5c and 5b. Gln307, Thr308, and Thr360 are the common amino acid residue involved in polar contacts. Ile17, Tyr311, and Trp358 are showing common hydrophobic interacting residues as shown in **Figure S2.**

Table S1. Glide Score, H-Bonding Interactions with Distances in Å, Polar, and Hydrophobic Interacting Residues for Investigated Ligands with target protein possessing tyrosinase activity (PDB ID: 2Y9W).

Lignd	gScore (kcal/mol)	Emodel (kcal/mol)	HBI residue (distance Å)	Polar interacting amino acid residues	Hydrophobic interacting residues
5c	-5.033	-60.546	Asp357 (1.97)	Gln307, Thr308, Thr360, Ser364	Ile17, Tyr311, Trp358, Phe368, Val371
5b	-5.005	-54.341	Glu359 (1.72, 2.02)	Gln307, Thr308, Thr360, Ser380	Ile17, Tyr311, Trp358

Abbreviation: HBI, hydrogen bonding interactions.

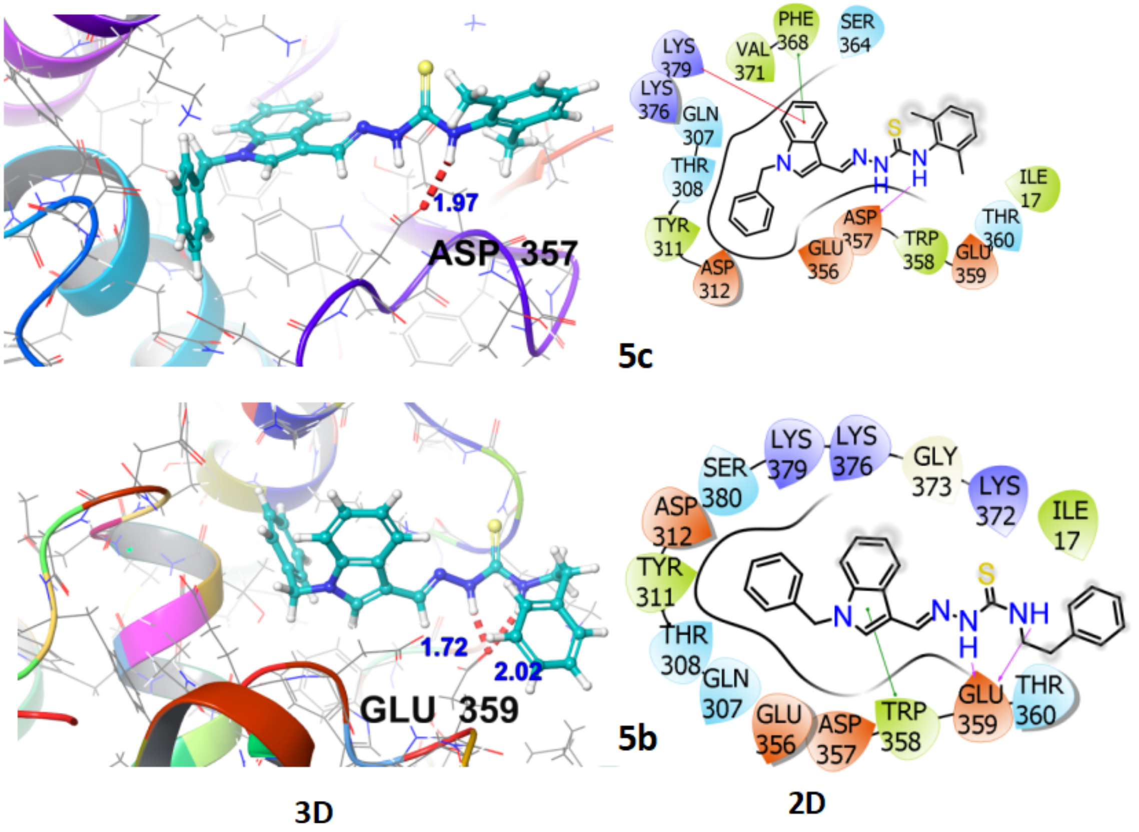

Figure S2. 3D and 2D representation of interaction with tyrosinase target protein (PDB ID: 2Y9W).

Table S2. Docking Score, glide Score, and glide emodel values of all investigated ligands with target protein possessing tyrosinase activity (PDB ID: 2Y9W).

**Ligand**	**docking score (kcal/mol)**	**glide gscore (kcal/mol)**	**glide emodel (kcal/mol)**
5k	-5.157	-5.157	-63.013
5c	-5.033	-5.033	-60.546
5b	-5.005	-5.005	-54.341
5a	-4.666	-4.666	-55.237
kojic acid	-4.416	-4.549	-34.051
5p	-4.313	-4.313	-45.738
5m	-4.236	-4.236	-47.798
5f	-4.129	-4.129	-54.012
5q	-4.033	-4.033	-55.055
5d	-3.876	-3.876	-42.321
5e	-3.815	-3.815	-46.426
5i	-3.778	-3.778	-49.164

5h	-3.554	-3.554	-49.496
5o	-3.454	-3.454	-48.434
5l	-3.311	-3.311	-48.872
5n	-3.183	-3.183	-46.608
5g	-3.085	-3.085	-42.012
5r	-2.983	-2.983	-42.112
5j	-2.185	-2.185	-34.915
co-crystallized-ligand-2Y9W	-0.129	-0.129	-19.187

Table S3. The ADMET Properties of all investigated ligands.

Ligand	Canonical SMILES	Formula	MW	#Heavy atoms	#Aromatic heavy atoms	Fraction Csp3	#Rotatable bonds	#H-bond acceptors	#H-bond donors	MR	TPSA	iLOGP	XLOGP3	WLOGP	MLOGP	Silicos-IT Log P
**5a**	COc1ccc(cc1)NC(=S)N/N=C/c1cn(c2c1cccc2)Cc1ccccc1	C24H22N4OS	414.52	30	21	0.08	8	2	2	127.06	82.67	3.9	4.8	4.83	3.28	5.19
**5b**	S=C(Nc1ccccc1)N/N=C/c1cn(c2c1cccc2)Cc1ccccc1	C23H20N4S	384.5	28	21	0.04	7	1	2	120.56	73.44	3.59	4.83	4.82	3.63	5.12
**5c**	S=C(Nc1c(C)cccc1C)N/N=C/c1cn(c2c1cccc2)Cc1ccccc1	C25H24N4S	412.55	30	21	0.12	7	1	2	130.5	73.44	3.95	5.56	5.44	4.04	6.18
**5d**	S=C(Nc1ccc(cc1)F)N/N=C/c1cn(c2c1cccc2)Cc1ccccc1	C23H19FN4S	402.49	29	21	0.04	7	2	2	120.52	73.44	3.73	4.93	5.38	4	5.54
**5e**	S=C(NCc1ccc(cc1)	C24H21	432.97	30	21	0.08	8	1	2	128.84	73.44	4.2	5.4	5.19	4.04	6.16

	Cl)N/N=C/c1cn(c2c1cccc2)Cc1ccccc1	ClN4S														
**5f**	S=C(Nc1ccc(c1Cl)Cl)N/N=C/c1cn(c2c1cccc2)Cc1ccccc1	C23H18Cl2N4S	453.39	30	21	0.04	7	1	2	130.58	73.44	4.05	6.09	6.13	4.58	6.4
**5g**	S=C(Nc1ccccc1)N/N=C/c1cn(c2c1cccc2)Cc1ccccc1	C23H20N4S	384.5	28	21	0.04	7	1	2	120.56	73.44	3.59	4.83	4.82	3.63	5.12
**5h**	S=C(Nc1ccc(cc1C)C)N/N=C/c1cn(c2c1cccc2)Cc1ccccc1	C25H24N4S	412.55	30	21	0.12	7	1	2	130.5	73.44	3.82	5.56	5.44	4.04	6.18
**5i**	S=C(NCc1ccccc1)N/N=C/c1cn(c2c1cccc2)Cc1ccccc1	C24H22N4S	398.52	29	21	0.08	8	1	2	123.83	73.44	3.97	4.77	4.54	3.57	5.52
**5j**	S=C(Nc1ccc(cc1)Br)N/N=C/c1cn(c2c1cccc2)Cc1ccccc1	C23H19BrN4S	463.39	29	21	0.04	7	1	2	128.26	73.44	4.09	5.52	5.58	4.21	5.8
**5k**	S=C(NCc1ccc(cc1)C)N/N=C/c1cn(c2c1cccc2)Cc1ccccc1	C25H24N4S	412.55	30	21	0.12	8	1	2	128.8	73.44	4.12	5.13	4.84	3.78	6.05
**5l**	COc1cccc(c1)NC(=S)N/N=C/c1cn(c2c1cccc2)Cc1ccccc1	C24H22N4OS	414.52	30	21	0.08	8	2	2	127.06	82.67	3.94	4.8	4.83	3.28	5.19
**5**	COc1cccc	C25	444.55	32	21	0.12	9	3	2	133.55	91.9	4.02	4.78	4.84	2.94	5.26

m	(c1NC(=S)N/N=C/c1cn(c2c1cccc2)Cc1ccccc1)OC	H24N4O2S														
5n	S=C(Nc1ccc(cc1)C)N/N=C/c1cn(c2c1cccc2)Cc1ccccc1	C24H22N4S	398.52	29	21	0.08	7	1	2	125.53	73.44	3.94	5.2	5.13	3.84	5.65
5o	S=C(Nc1cccc(c1)[N+](=O)[O-])N/N=C/c1cn(c2c1cccc2)Cc1ccccc1	C23H19N5O2S	429.49	31	21	0.04	8	3	2	129.39	119.26	3.3	4.66	4.73	2.67	2.96
5p	CNC(=S)N/N=C/c1cn(c2c1cccc2)Cc1ccccc1	C18H18N4S	322.43	23	15	0.11	6	1	2	99.35	73.44	2.91	3.27	3.12	2.46	4.04
5q	S=C(Nc1ccc(cc1)[N+](=O)[O-])N/N=C/c1cn(c2c1cccc2)Cc1ccccc1	C23H19N5O2S	429.49	31	21	0.04	8	3	2	129.39	119.26	3.27	4.66	4.73	2.67	2.96
5r	S=C(Nc1ccc2c(c1)cccc2)N/N=C/c1cn(c2c1cccc2)Cc1ccccc1	C27H22N4S	434.56	32	25	0.04	7	1	2	138.07	73.44	4.08	6.08	5.97	4.28	6.15
Kojic acid	OCc1occ(c(=O)c1)O	C6H6O4	142.11	10	6	0.17	1	4	2	33.13	70.67	1.12	-0.64	-0.31	-1.69	0.74

Ligand	Consensus Log P	ESOL Log S	ESOL Solubility	ESOL Solubility (mol/l)	ESOL Class	Ali Log S	Ali Solubility (mg/ml)	Ali Solubility	Ali Class	Silicos-IT LogSw	Silicos-IT Solubility (mg/ml)	Silicos-IT Solubility (mol/l)	Silicos-IT class	GI absorption	BBB permeant	Pgp substrate
5a	4.4	-5.42	1.56E-03	3.77E-06	Moderately soluble	-6.27	2.24E-04	5.39E-07	Poorly soluble	-8.28	2.17E-06	5.22E-09	Poorly soluble	High	No	No

5b	4.4	-5.36	1.68E-03	4.37E-06	Moderately soluble	-6.11	3.02E-04	7.84E-07	Poorly soluble	-8.18	2.54E-06	6.61E-09	Poorly soluble	High	No	No
**5c**	5.03	-5.96	4.56E-04	1.11E-06	Moderately soluble	-6.86	5.65E-05	1.37E-07	Poorly soluble	-8.93	4.84E-07	1.17E-09	Poorly soluble	High	No	Yes
**5d**	4.72	-5.52	1.23E-03	3.05E-06	Moderately soluble	-6.21	2.49E-04	6.18E-07	Poorly soluble	-8.44	1.45E-06	3.60E-09	Poorly soluble	High	No	No
**5e**	5	-5.92	5.25E-04	1.21E-06	Moderately soluble	-6.7	8.70E-05	2.01E-07	Poorly soluble	-9.16	3.01E-07	6.95E-10	Poorly soluble	High	No	Yes
**5f**	5.45	-6.54	1.30E-04	2.86E-07	Poorly soluble	-7.41	1.75E-05	3.86E-08	Poorly soluble	-9.35	2.03E-07	4.48E-10	Poorly soluble	High	No	No
**5g**	4.4	-5.36	1.68E-03	4.37E-06	Moderately soluble	-6.11	3.02E-04	7.84E-07	Poorly soluble	-8.18	2.54E-06	6.61E-09	Poorly soluble	High	No	No
**5h**	5.01	-5.96	4.56E-04	1.11E-06	Moderately soluble	-6.86	5.65E-05	1.37E-07	Poorly soluble	-8.93	4.84E-07	1.17E-09	Poorly soluble	High	No	Yes
**5i**	4.47	-5.32	1.89E-03	4.74E-06	Moderately soluble	-6.04	3.61E-04	9.05E-07	Poorly soluble	-8.57	1.07E-06	2.67E-09	Poorly soluble	High	No	Yes
**5j**	5.04	-6.26	2.52E-04	5.44E-07	Poorly soluble	-6.82	6.99E-05	1.51E-07	Poorly soluble	-8.96	5.09E-07	1.10E-09	Poorly soluble	High	No	No
**5k**	4.78	-5.62	9.90E-04	2.40E-06	Moderately soluble	-6.42	1.58E-04	3.83E-07	Poorly soluble	-8.95	4.65E-07	1.13E-09	Poorly soluble	High	No	Yes
**5l**	4.41	-5.42	1.56E-03	3.77E-06	Moderately soluble	-6.27	2.24E-04	5.39E-07	Poorly soluble	-8.28	2.17E-06	5.22E-09	Poorly soluble	High	No	No
**5m**	4.37	-5.5	1.41E-03	3.17E-06	Moderately soluble	-6.44	1.61E-04	3.62E-07	Poorly soluble	-8.38	1.85E-06	4.16E-09	Poorly soluble	High	No	No
**5n**	4.75	-	8.70	2.18E-06	Mode	-	1.29E-04	3.24	Poor	-8.56	1.11E-06	2.78E-	Poor	High	No	No

		5.66	E-04		rately soluble	6.49		E-07	ly soluble			09	ly soluble			
**5o**	3.66	-5.41	1.66E-03	3.87E-06	Moderately soluble	-6.89	5.52E-05	1.28E-07	Poorly soluble	-7.52	1.30E-05	3.02E-08	Poorly soluble	Low	No	No
**5p**	3.16	-3.99	3.33E-02	1.03E-04	Soluble	-4.49	1.05E-02	3.26E-05	Moderately soluble	-6.09	2.60E-04	8.07E-07	Poorly soluble	High	Yes	No
**5q**	3.66	-5.41	1.66E-03	3.87E-06	Moderately soluble	-6.89	5.52E-05	1.28E-07	Poorly soluble	-7.52	1.30E-05	3.02E-08	Poorly soluble	Low	No	No
**5r**	5.31	-6.48	1.44E-04	3.31E-07	Poorly soluble	-7.4	1.72E-05	3.96E-08	Poorly soluble	-9.81	6.71E-08	1.55E-10	Poorly soluble	High	No	No
**Kojic acid**	-0.16	-0.7	2.86E+01	2.01E-01	Very soluble	-0.37	6.05E+01	4.25E-01	Very soluble	-1.17	9.72E+00	6.84E-02	Soluble	High	No	No

**Ligand**	**CYP1A2 inhibitor**	**CYP2C19 inhibitor**	**CYP2C9 inhibitor**	**CYP2D6 inhibitor**	**CYP3A4 inhibitor**	**log Kp (cm/s)**	**Lipinski #violations**	**Ghose #violations**	**Veber #violations**	**Egan #violations**	**Muegge #violations**	**Bioavailability Score**	**PAINS #alerts**	**Brenk #alerts**	**Leadlikeness #violations**	**Synthetic Accessibility**
**5a**	Yes	Yes	Yes	Yes	Yes	-5.42	0	0	0	0	0	0.55	1	2	3	3.2
**5b**	Yes	Yes	Yes	Yes	Yes	-5.22	0	0	0	0	0	0.55	1	2	2	3.14
**5c**	Yes	Yes	Yes	Yes	Yes	-4.87	0	1	0	0	1	0.55	1	2	2	3.38
**5d**	Yes	Yes	Yes	Yes	Yes	-5.25	0	0	0	0	0	0.55	1	2	2	3.12
**5e**	Yes	Yes	Yes	Yes	Yes	-5.11	0	0	0	0	1	0.55	0	2	3	3.2
**5f**	No	Yes	Yes	No	Yes	-4.74	1	2	0	1	1	0.55	1	2	2	3.26
**5g**	Yes	Yes	Yes	Yes	Yes	-5.22	0	0	0	0	0	0.55	1	2	2	3.14
**5h**	Yes	Yes	Yes	Yes	Yes	-4.87	0	1	0	0	1	0.55	1	2	2	3.39
**5i**	Yes	Yes	Yes	Yes	Yes	-5.34	0	0	0	0	0	0.55	0	2	3	3.19
**5j**	Yes	Yes	Yes	Yes	Yes	-5.21	1	0	0	0	1	0.55	1	2	2	3.2
**5k**	Yes	Yes	Yes	Yes	Yes	-5.17	0	0	0	0	1	0.55	0	2	3	3.3
**5l**	Yes	Yes	Yes	Yes	Yes	-5.42	0	0	0	0	0	0.55	1	2	3	3.29
**5m**	No	Yes	Yes	Yes	Yes	-5.62	0	1	0	0	0	0.55	1	2	3	3.51
**5n**	Yes	Yes	Yes	Yes	Yes	-5.04	0	0	0	0	1	0.55	1	2	2	3.26
**5o**	No	Yes	Yes	No	Yes	-5.61	0	0	0	0	0	0.55	1	4	3	3.49
**5p**	Yes	Yes	Yes	Yes	Yes	-5.95	0	0	0	0	0	0.55	0	2	0	2.75
**5q**	No	Yes	Yes	No	Yes	-5.61	0	0	0	0	0	0.55	1	4	3	3.3
**5r**	Yes	Yes	Yes	No	Yes	-4.63	1	2	0	1	1	0.55	1	2	2	3.37

**Kojic acid**	No	No	No	No	No	-7.62	0	3	0	0	1	0.55	0	0	1	2.53

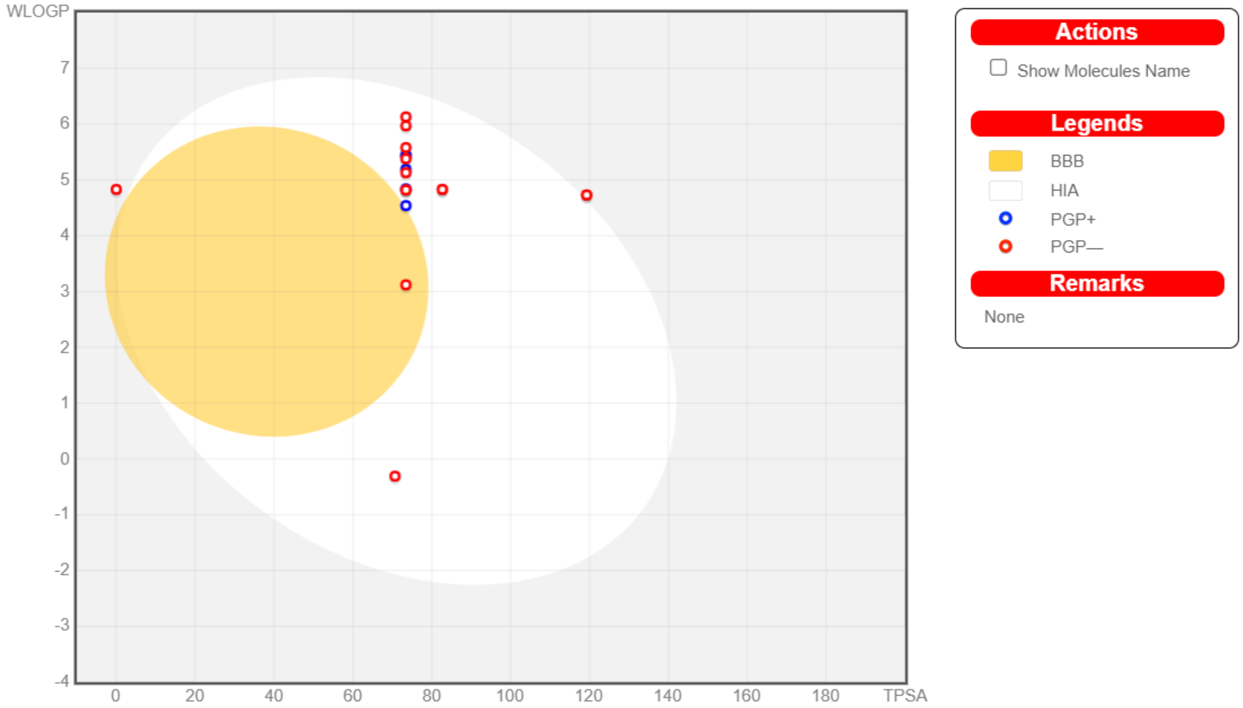

Figure S3. The boiled-egg plot (plot of WLOGP against TPSA) of all investigated compounds from SwissADME web tool.

1H NMR and 13C NMR Spectra of 5a

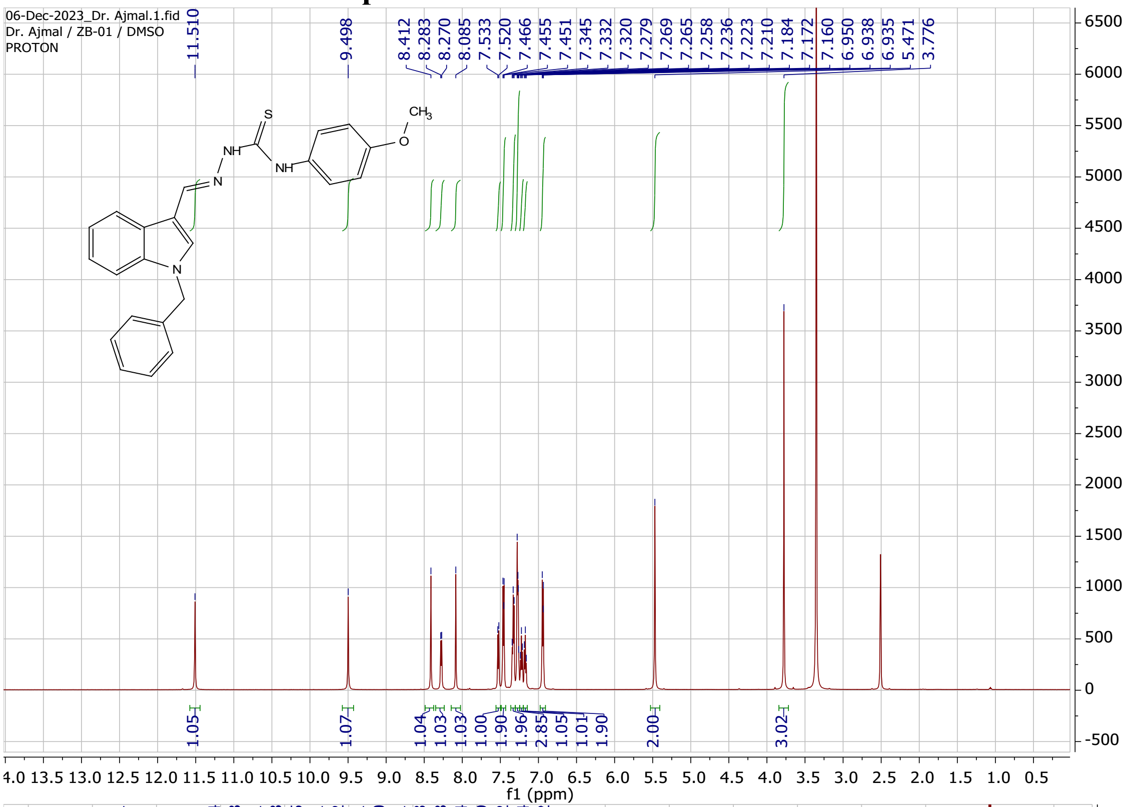

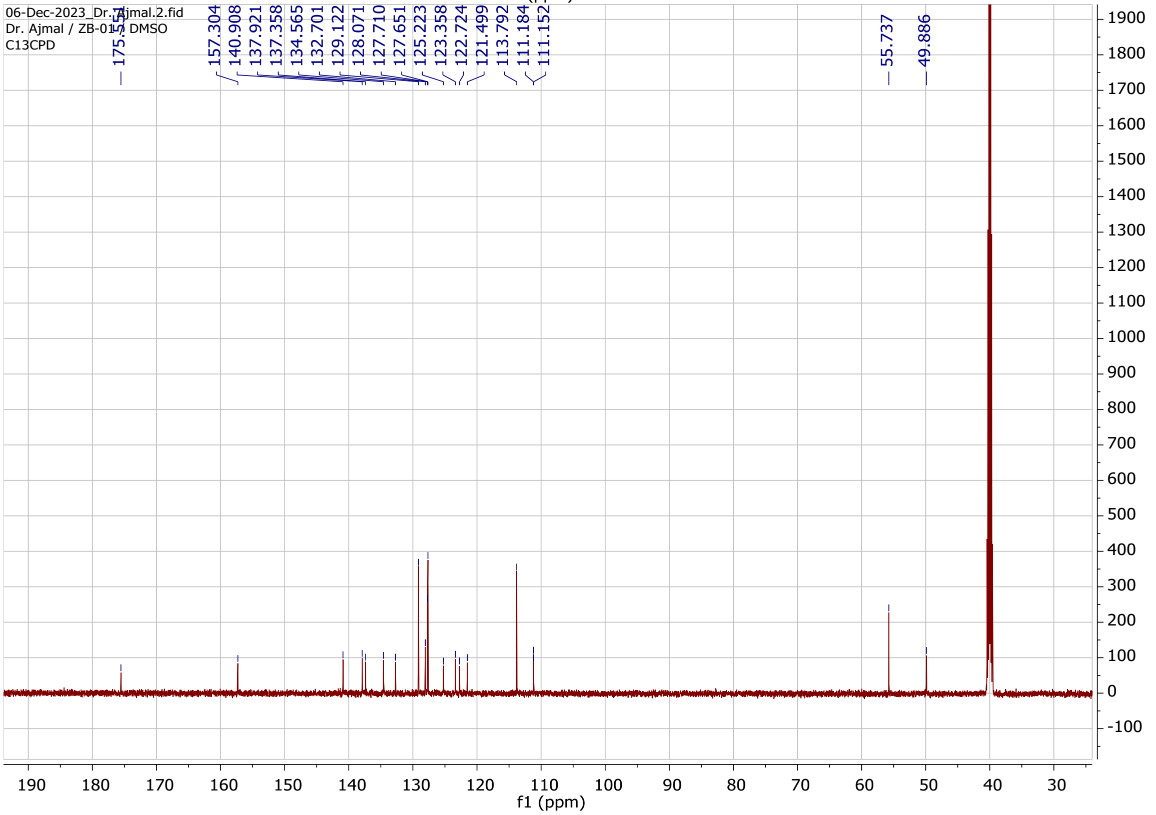

1H NMR and 13C NMR Spectra of 5b

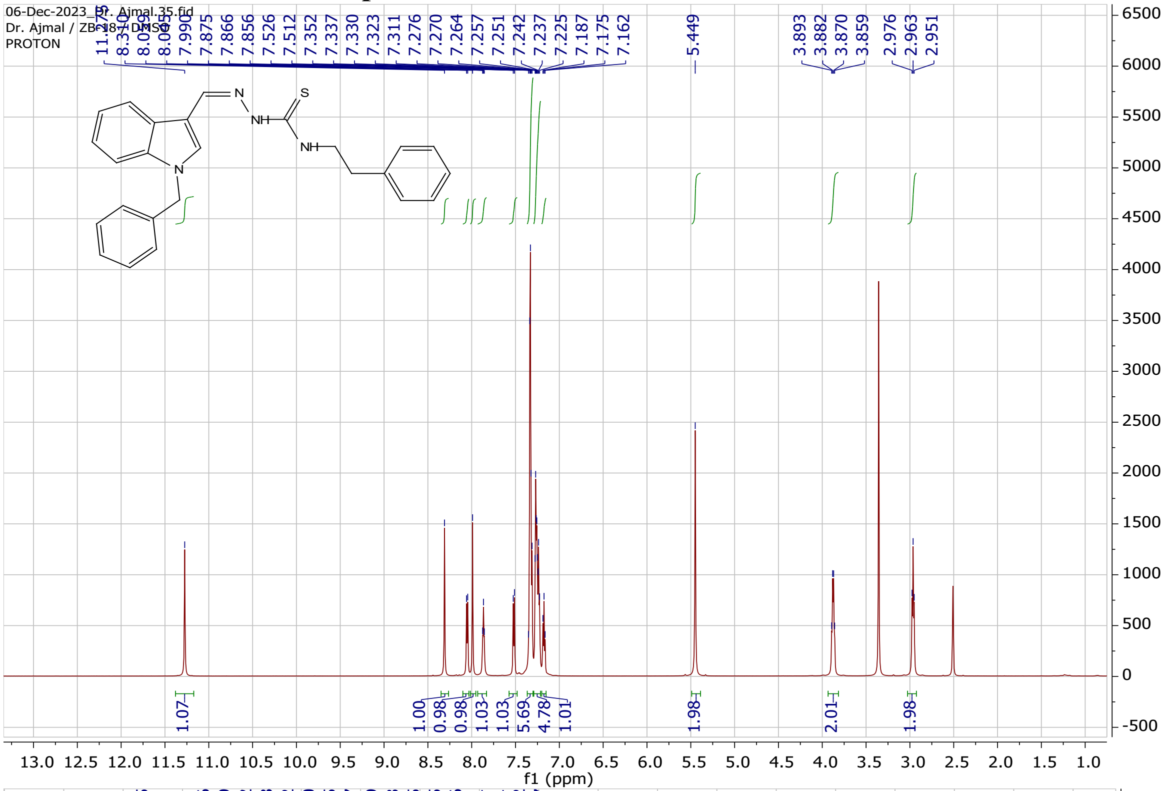

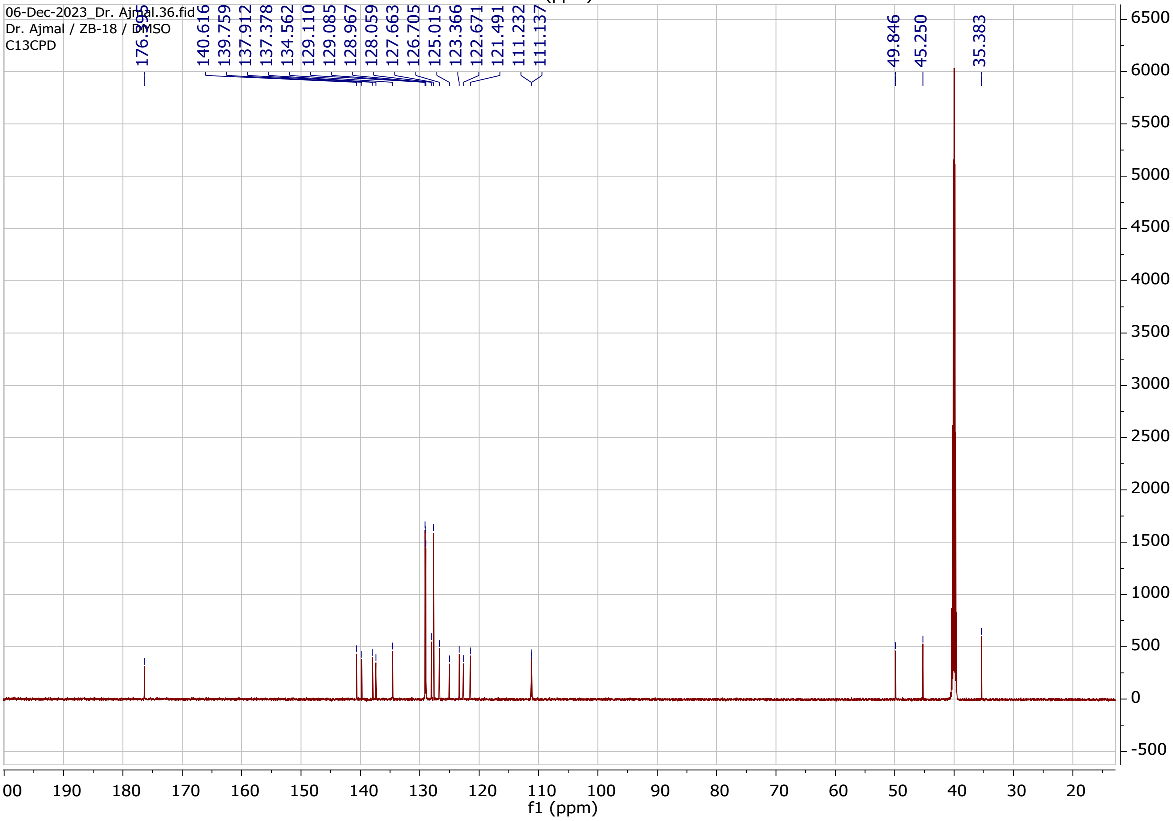

1H NMR and 13C NMR Spectra of 5c

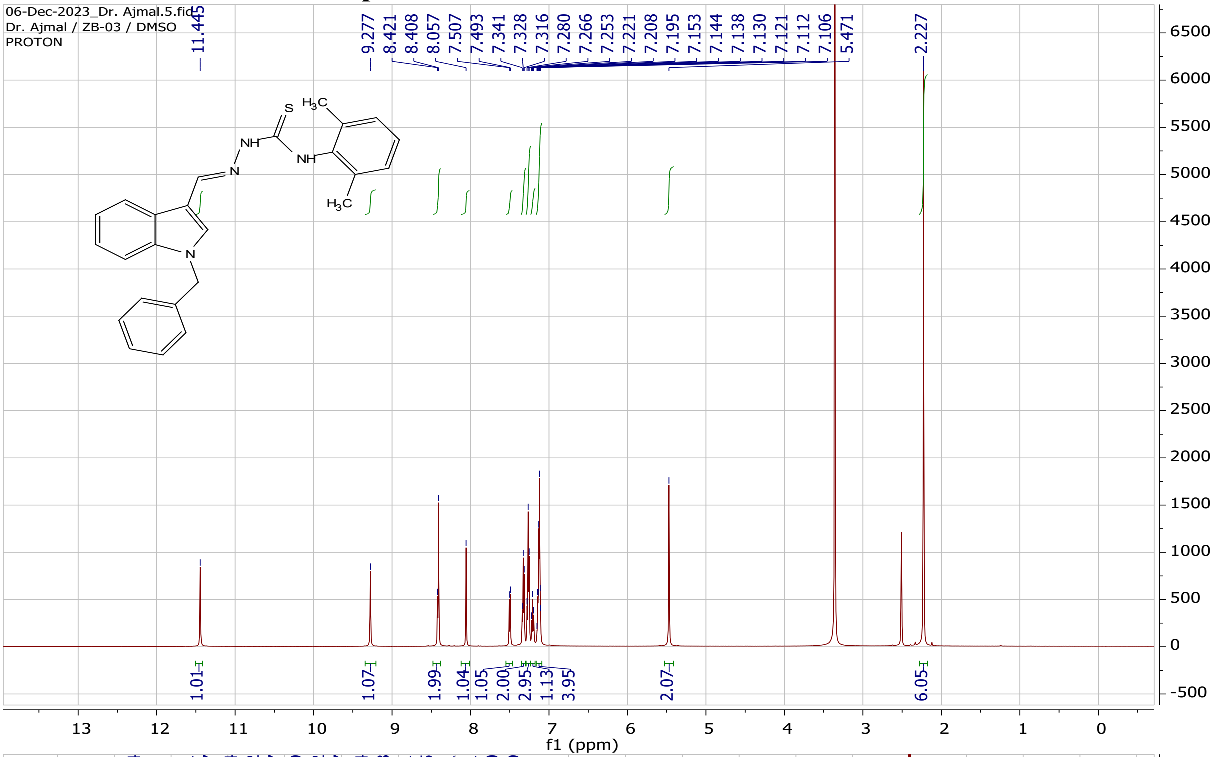

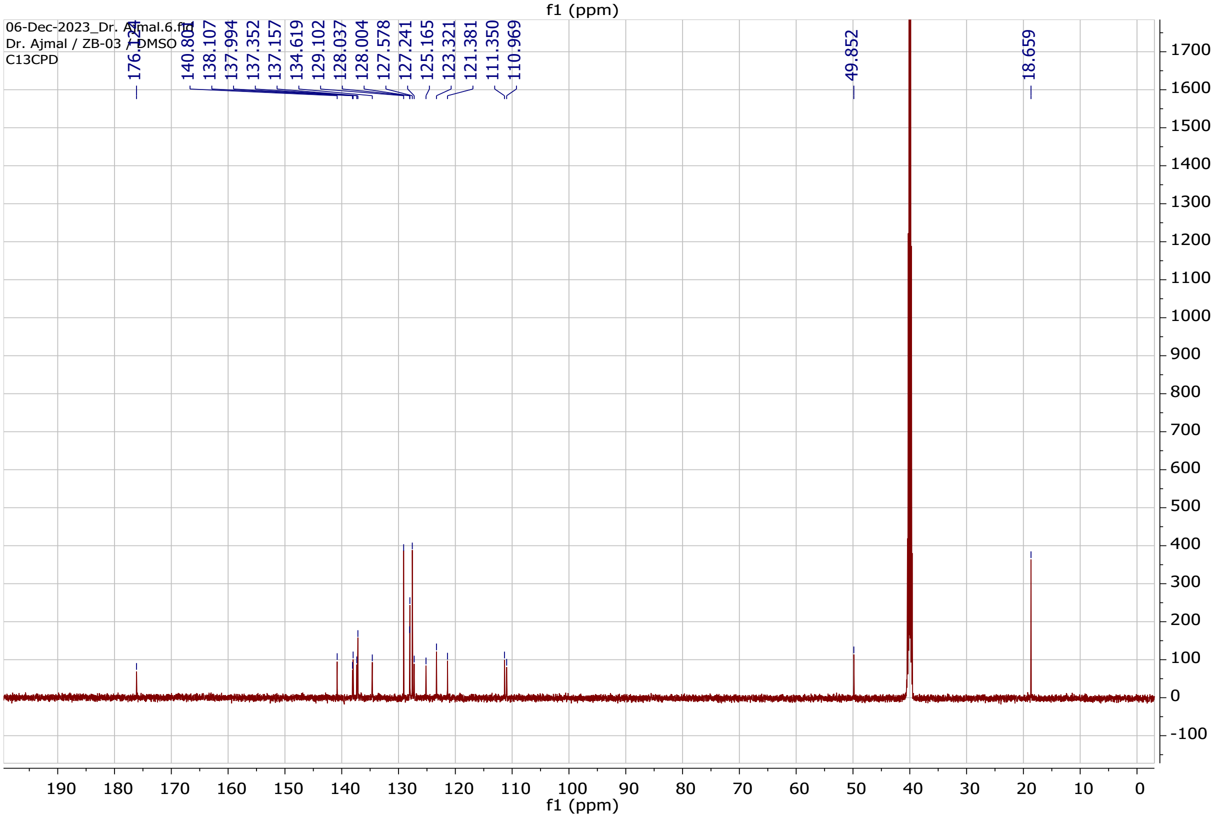

1H NMR and 13C NMR Spectra of 5d

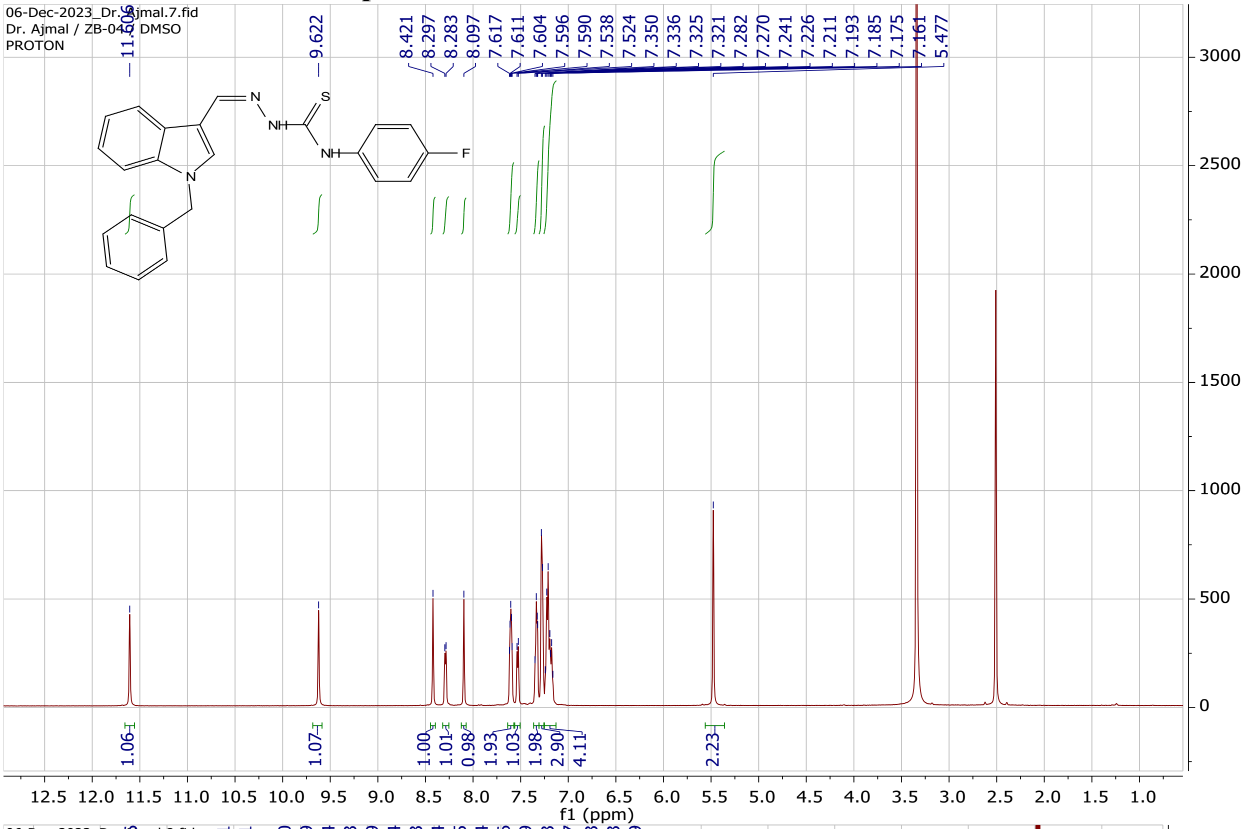

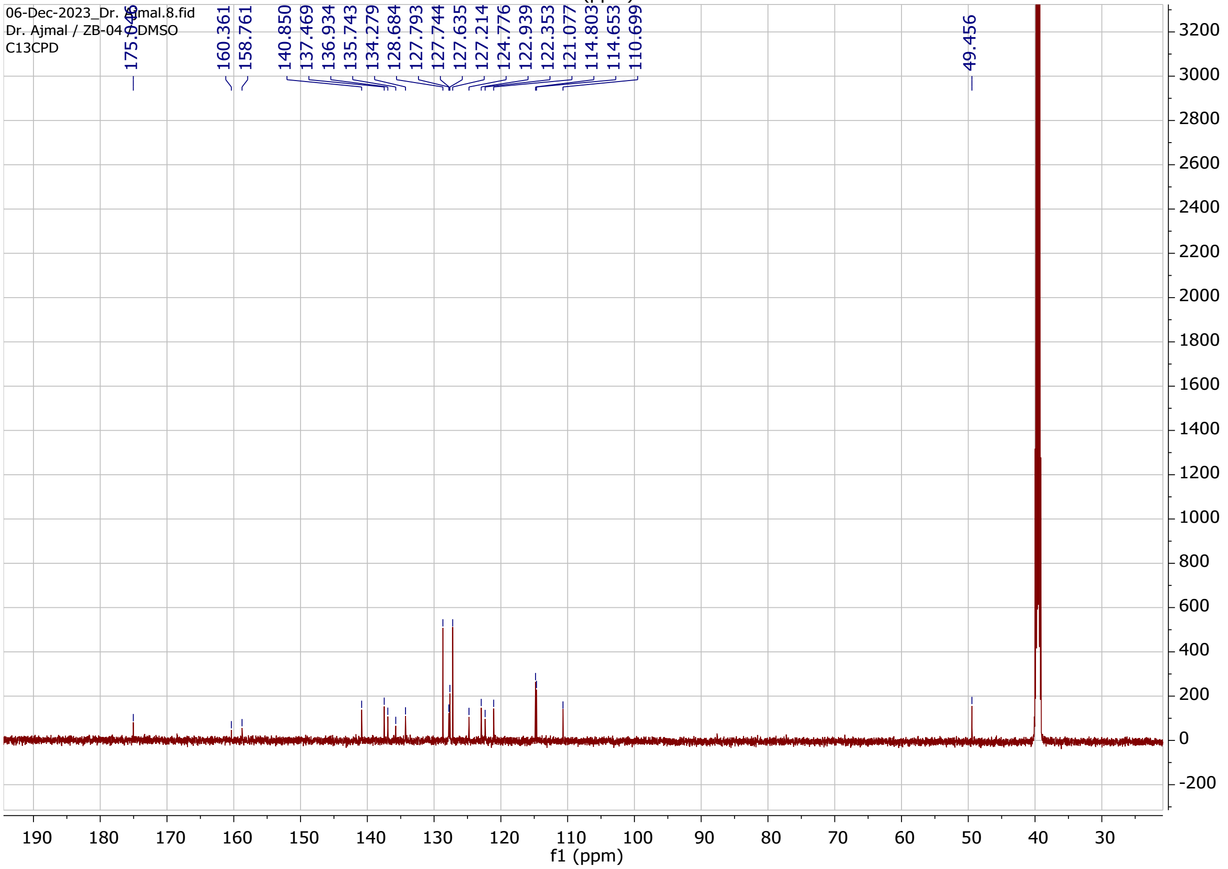

1H NMR and 13C NMR Spectra of 5e

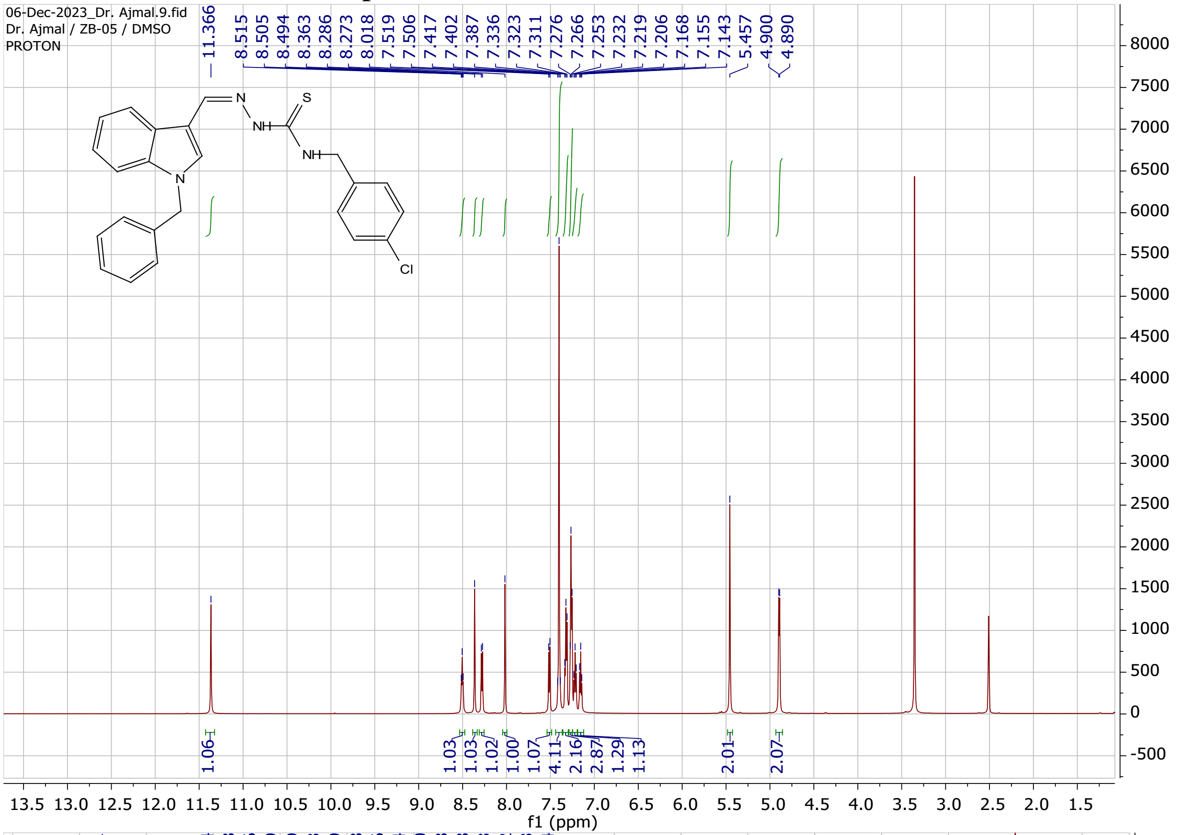

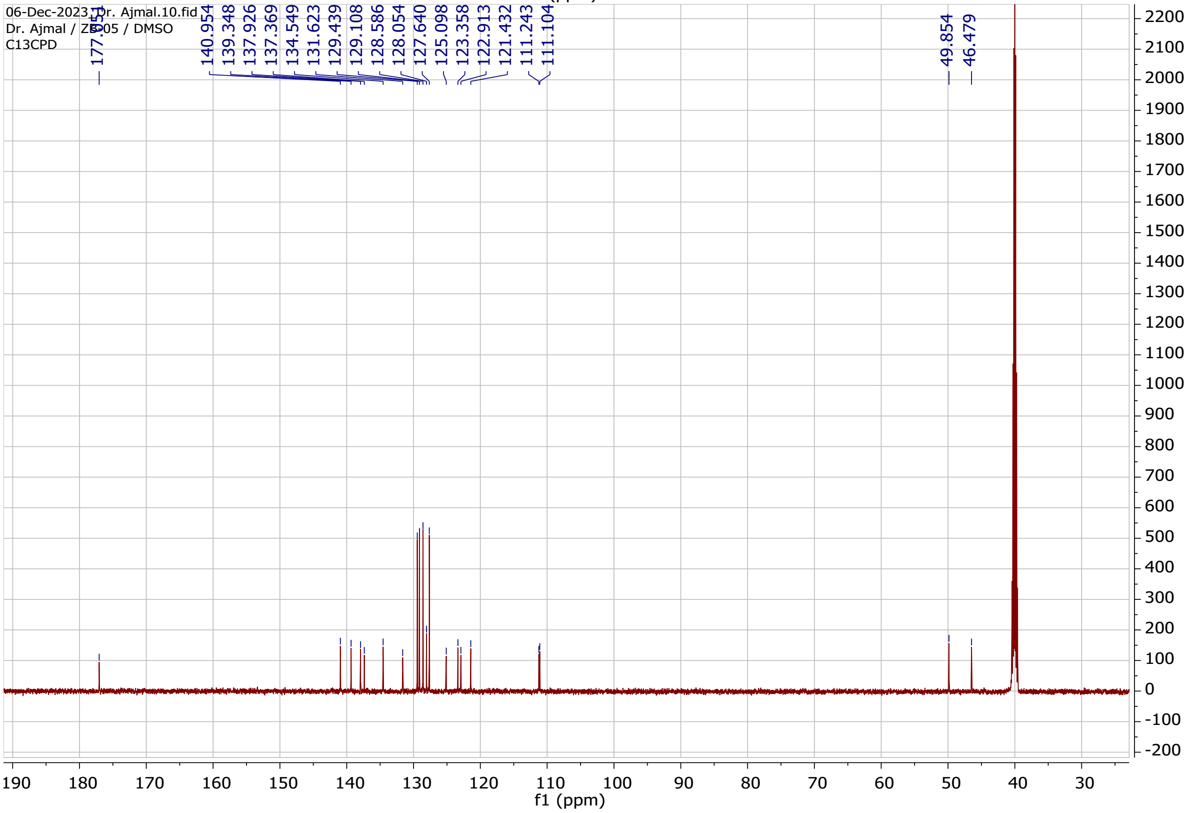

1H NMR and 13C NMR Spectra of 5f

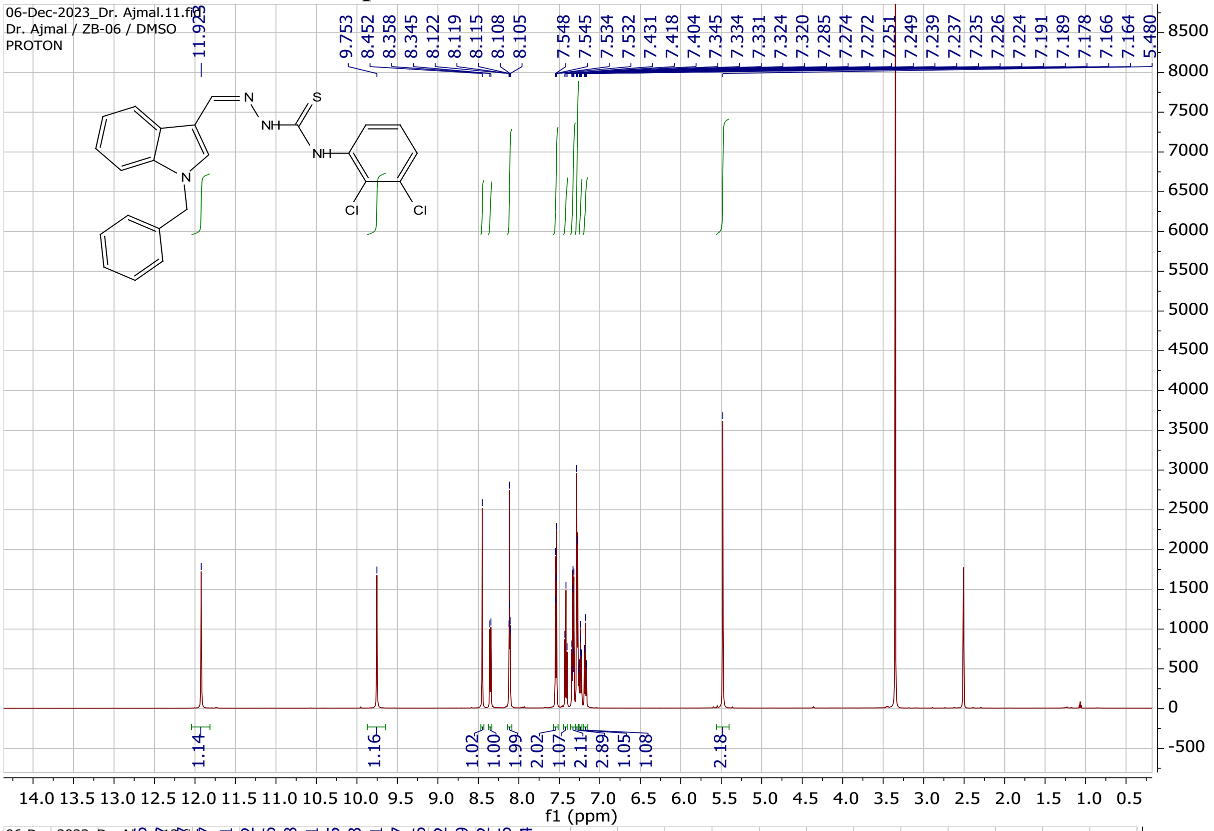

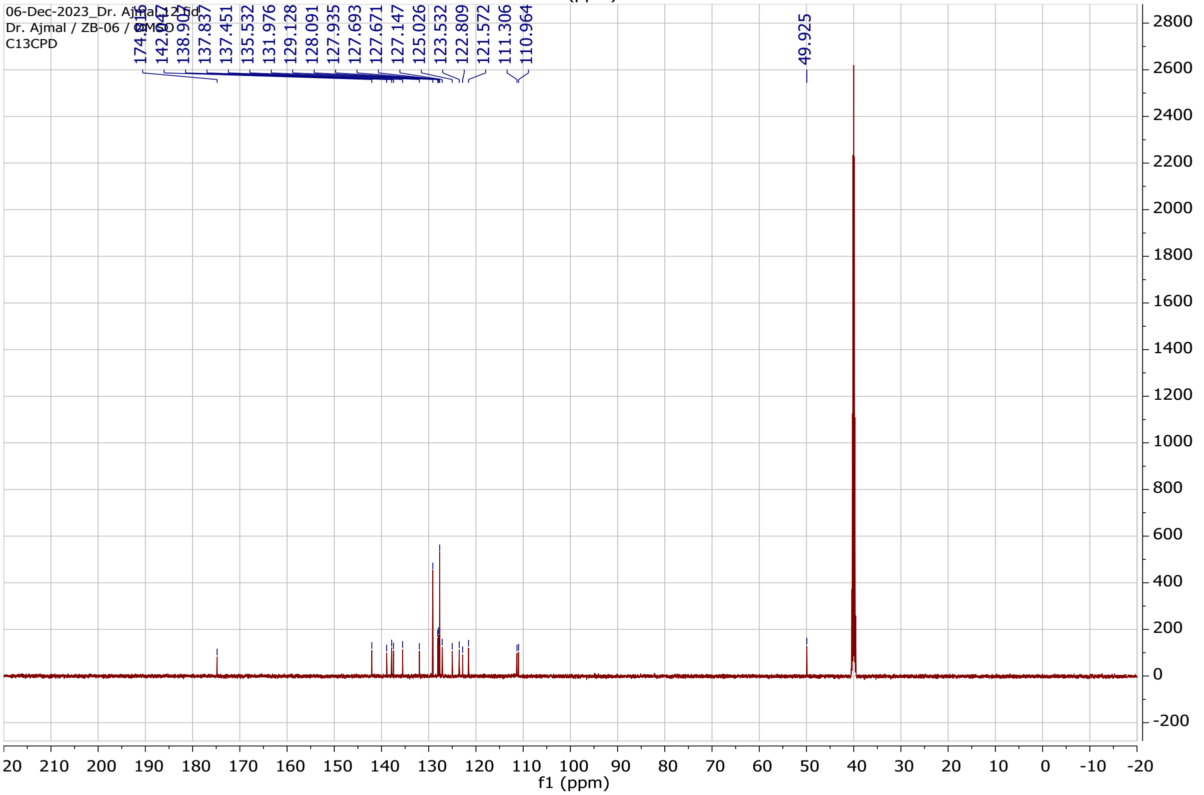

H NMR and 13C NMR Spectra of 5g

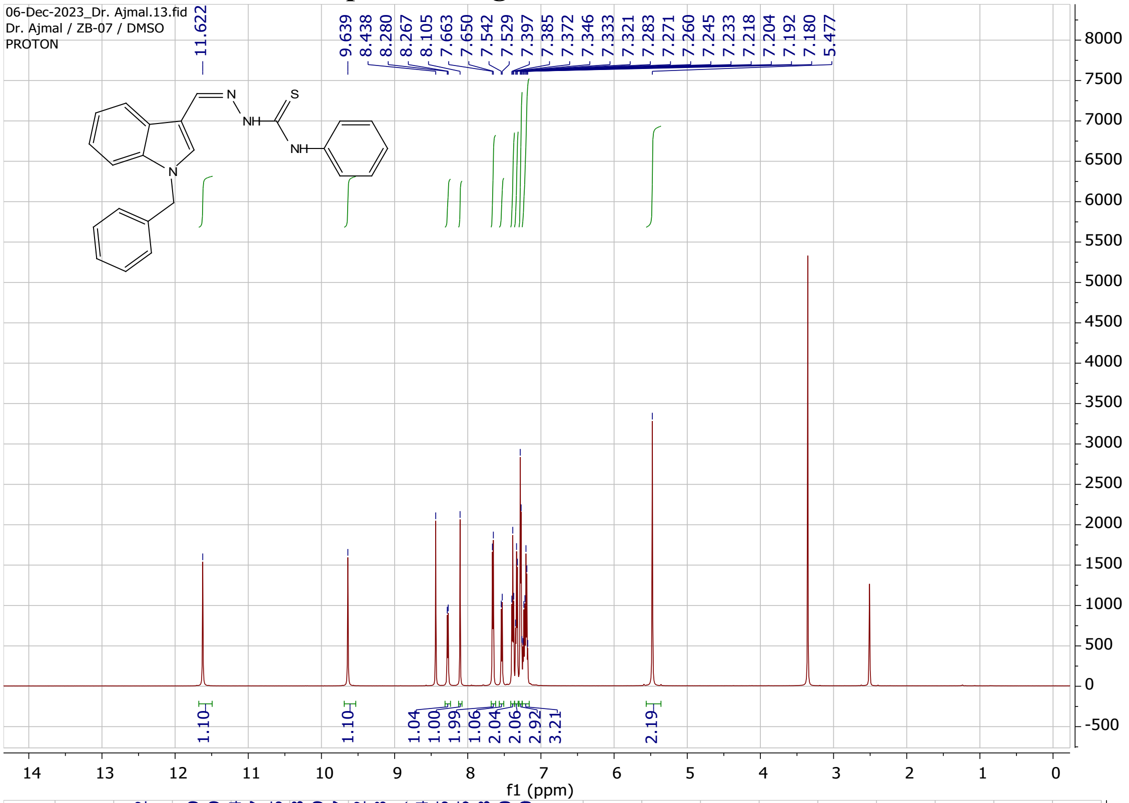

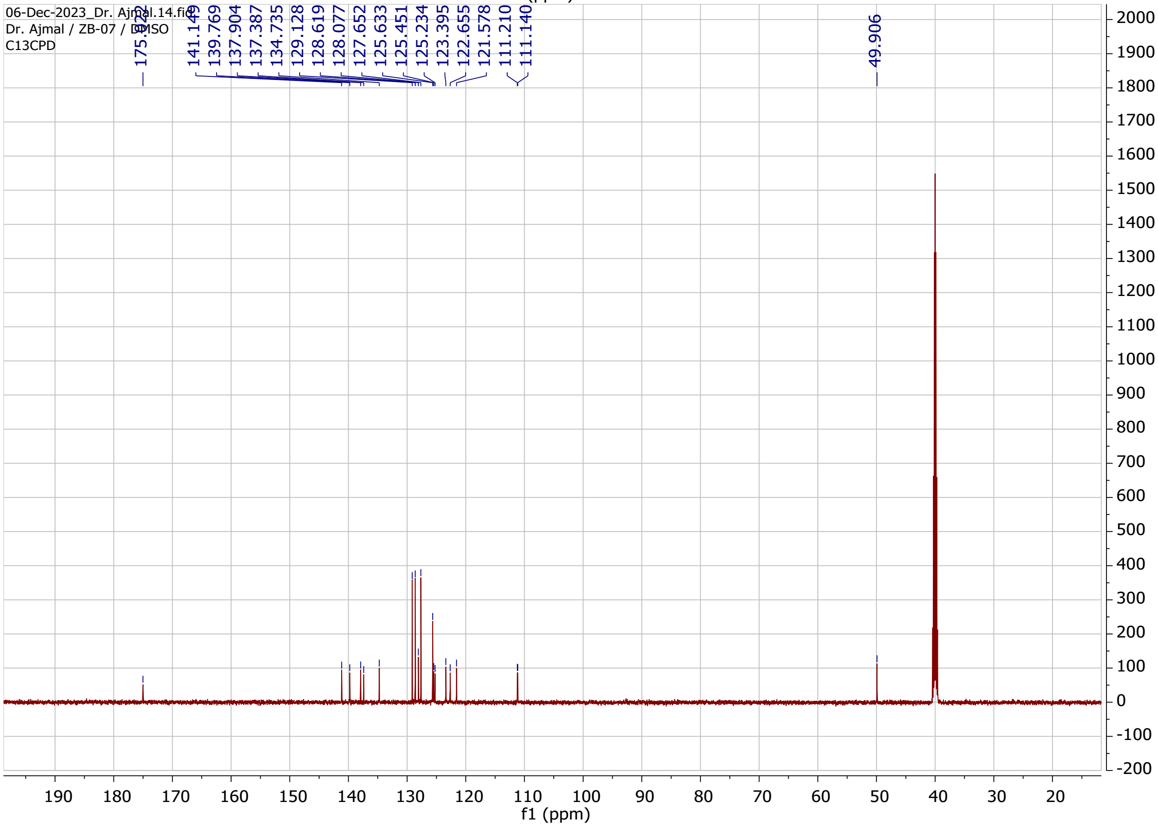

H NMR and 13C NMR Spectra of 5h

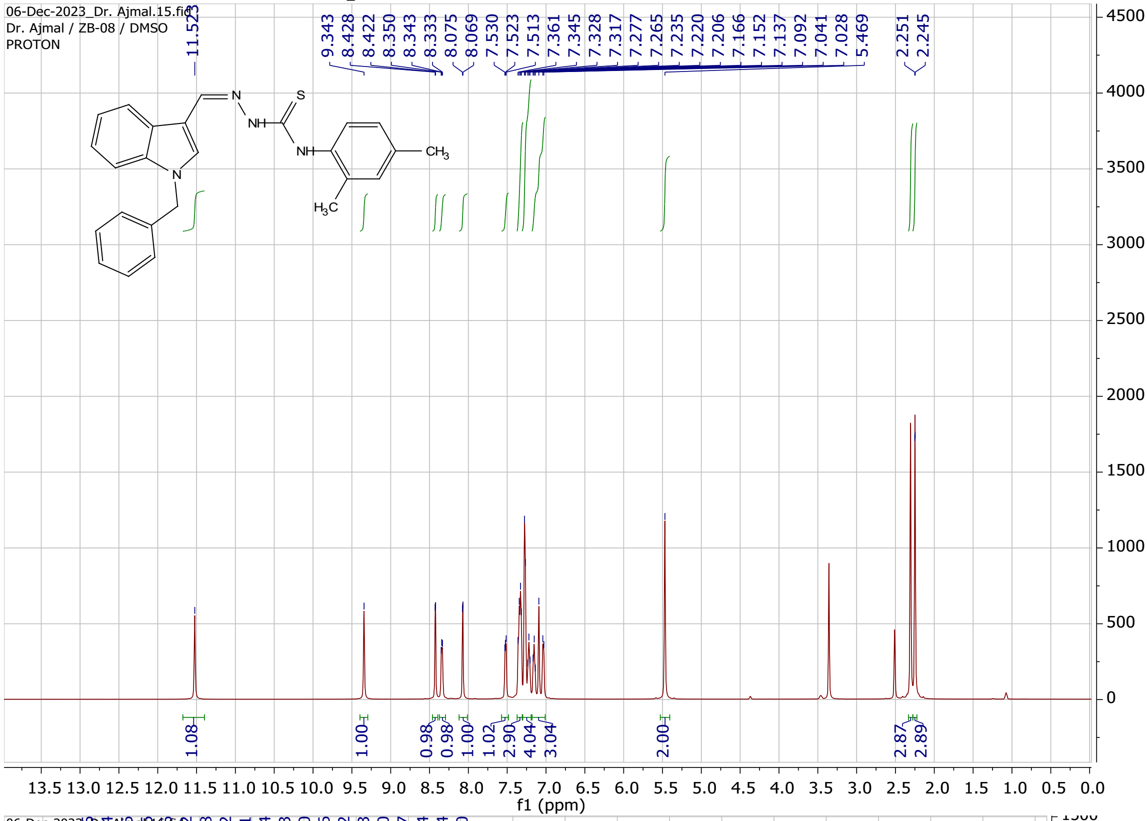

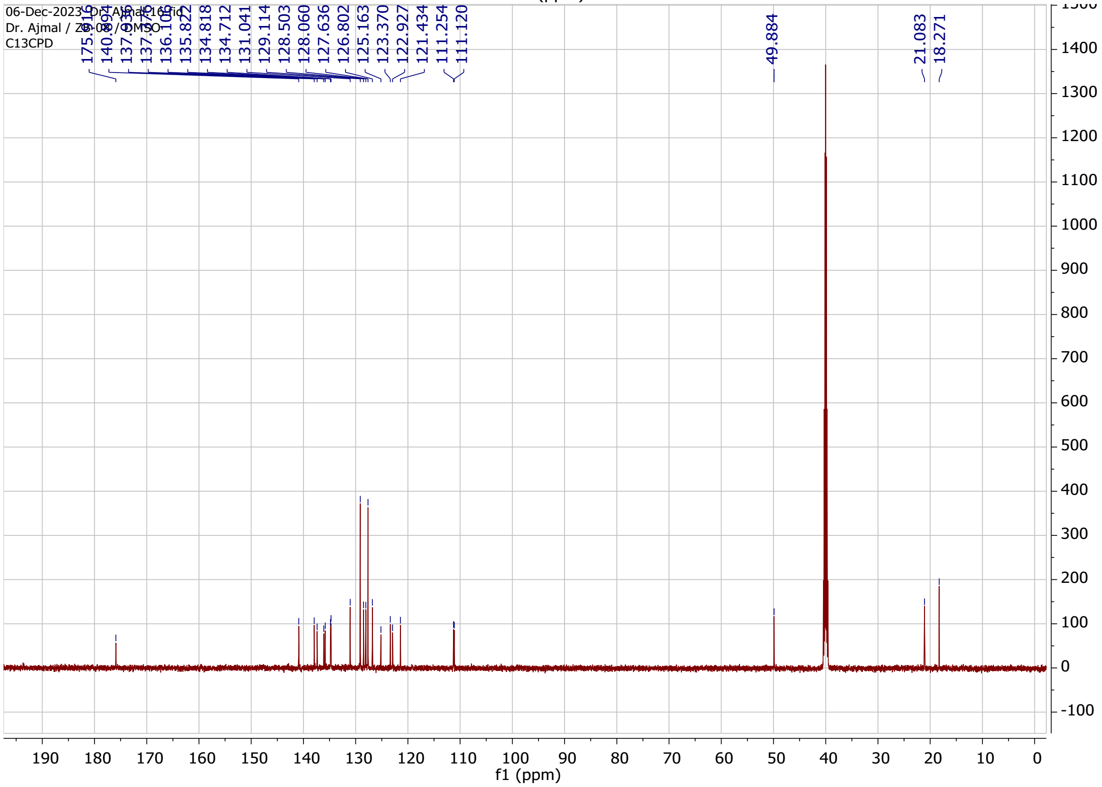

1H NMR and 13C NMR Spectra of 5i

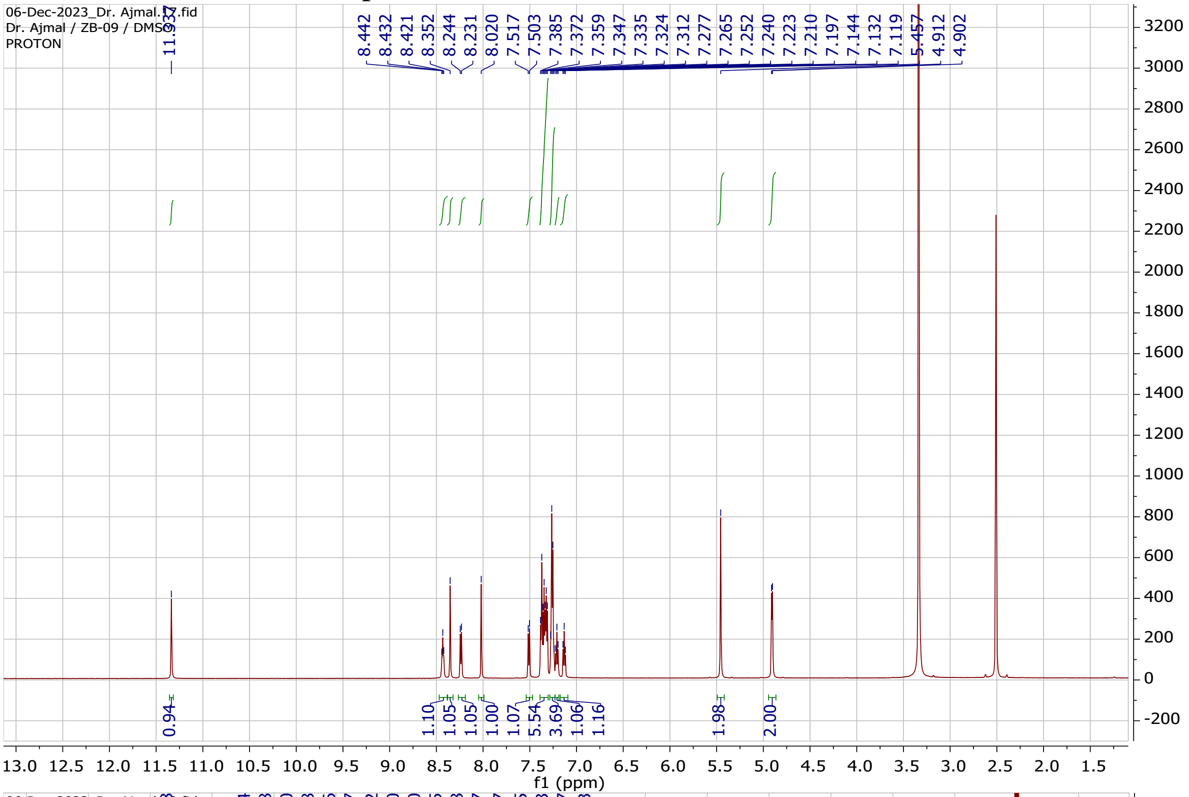

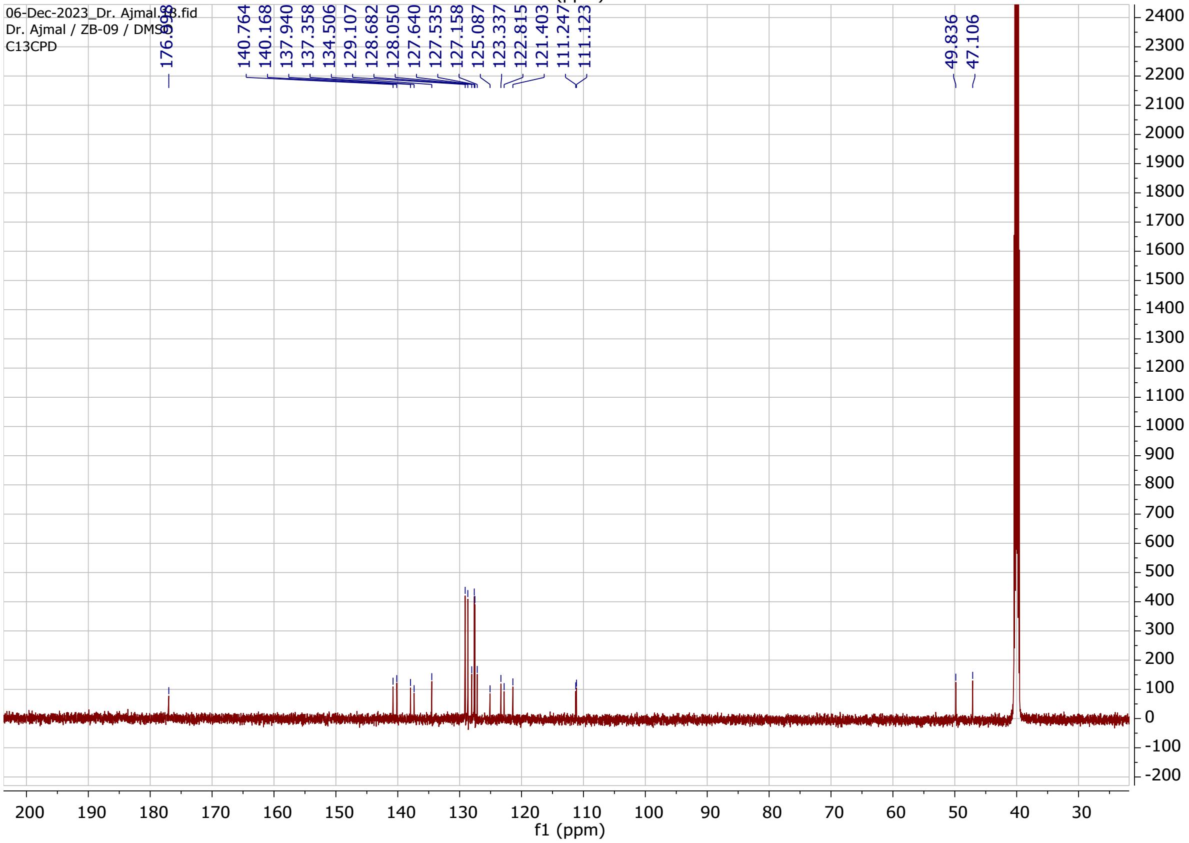

1H NMR and 13C NMR Spectra of 5j

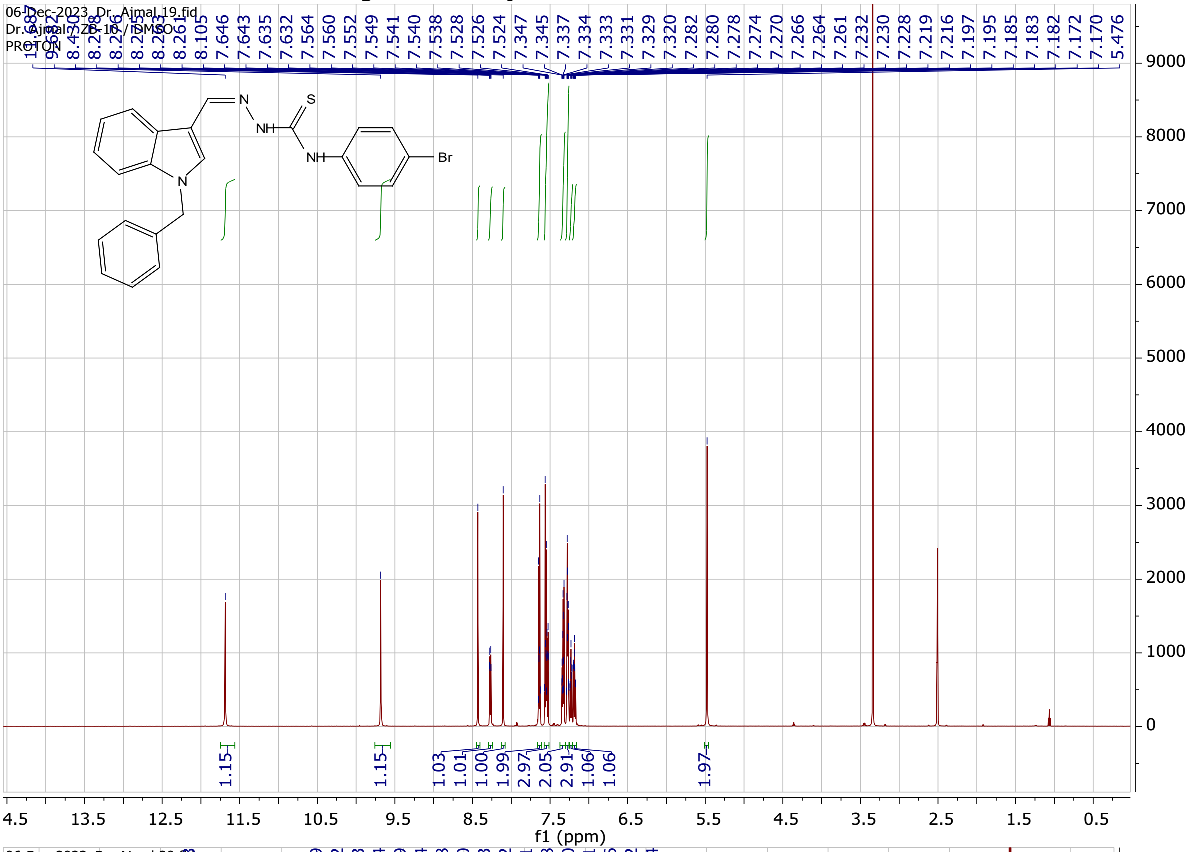

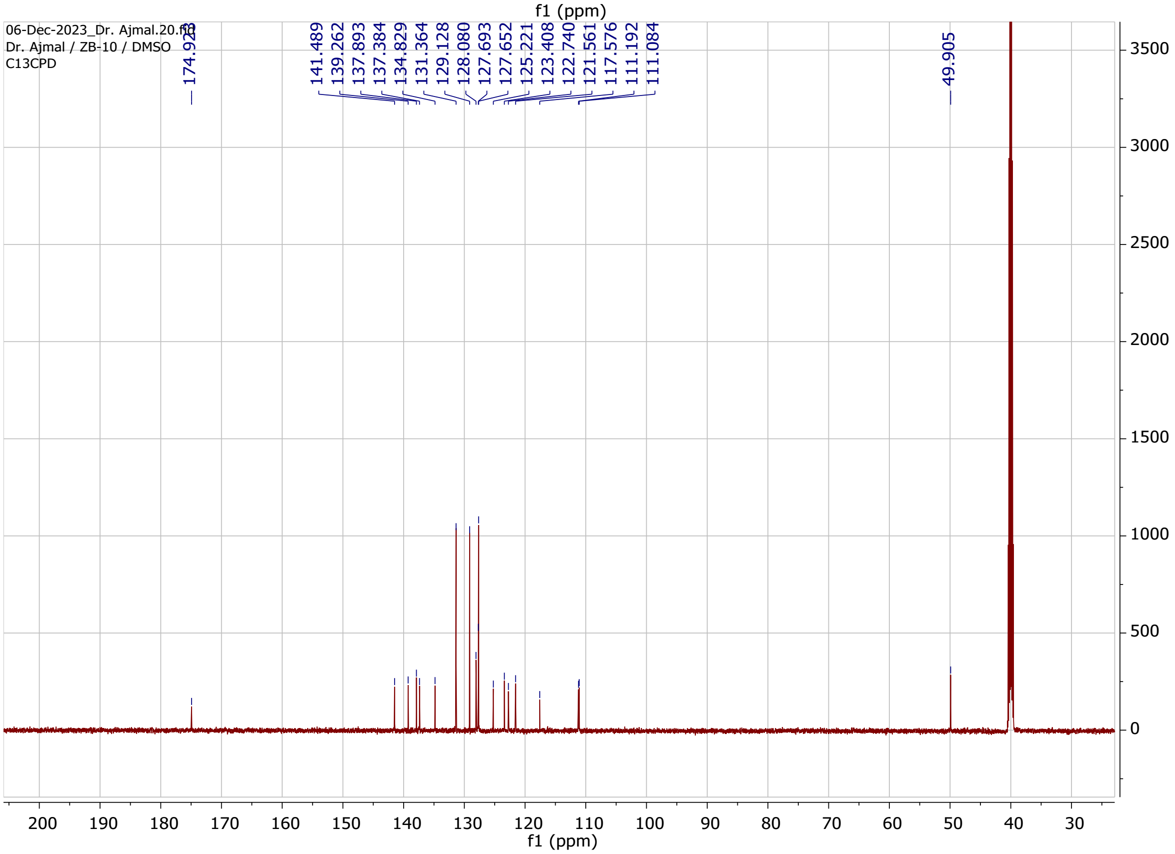

^{1}H NMR and ^{13}C NMR Spectra of 5k

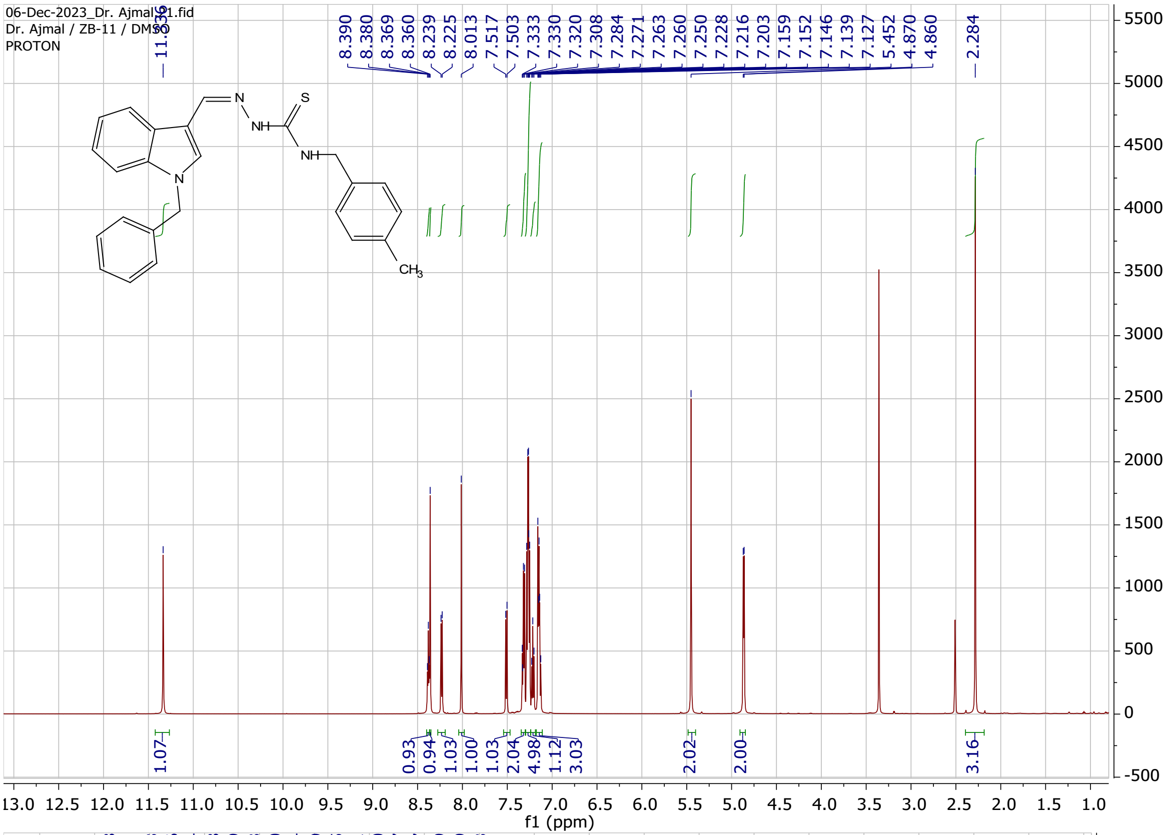

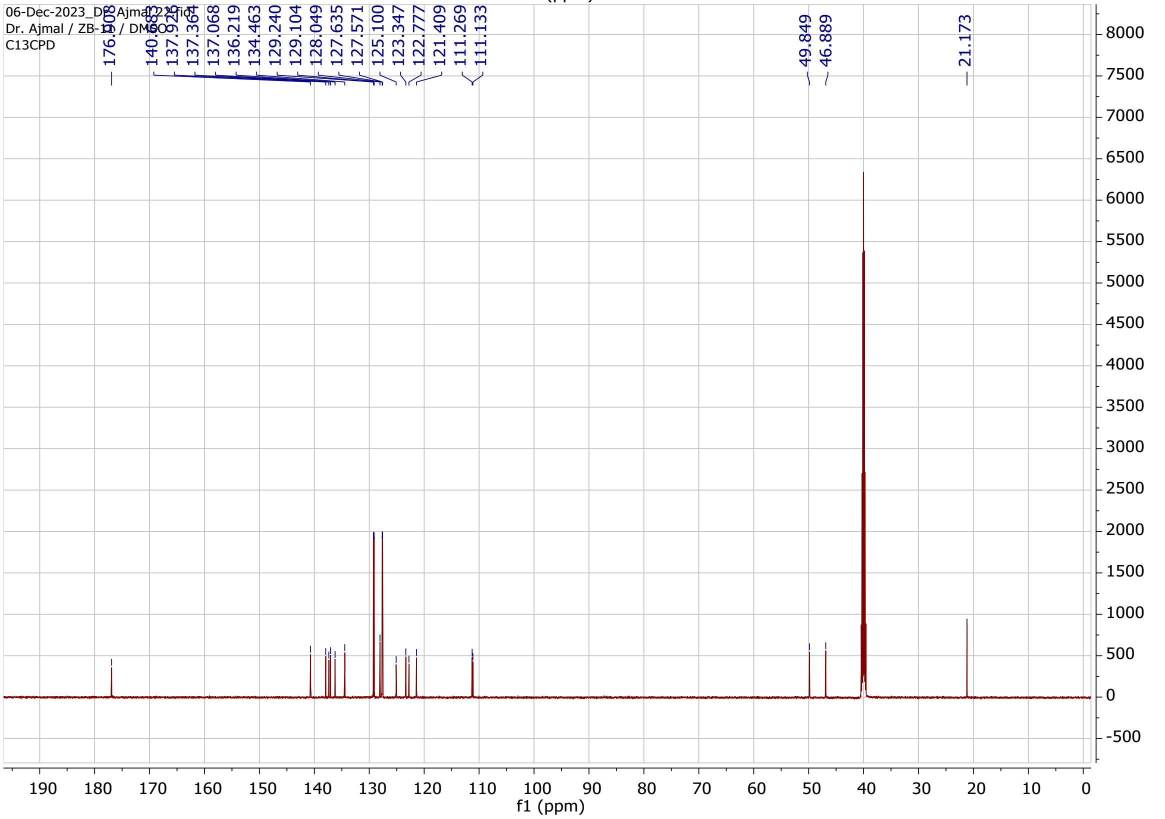

1H NMR and 13C NMR Spectra of 5l

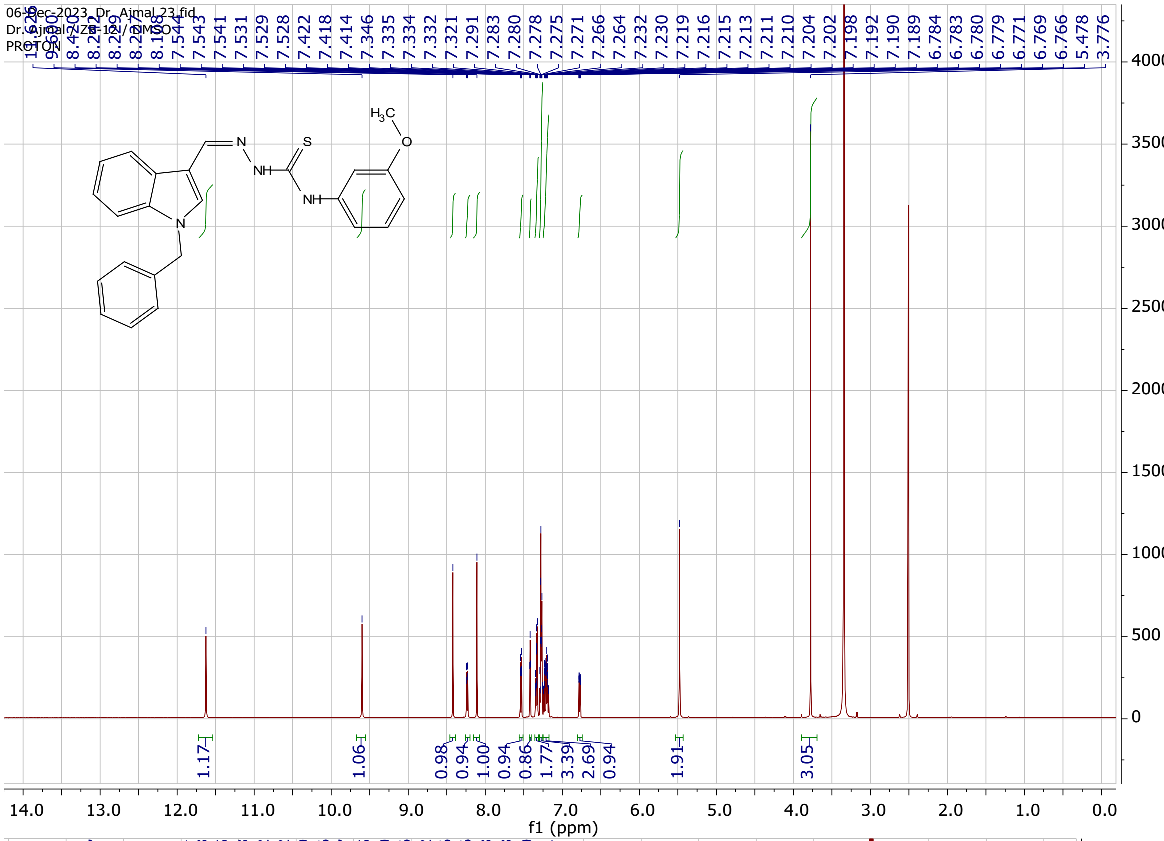

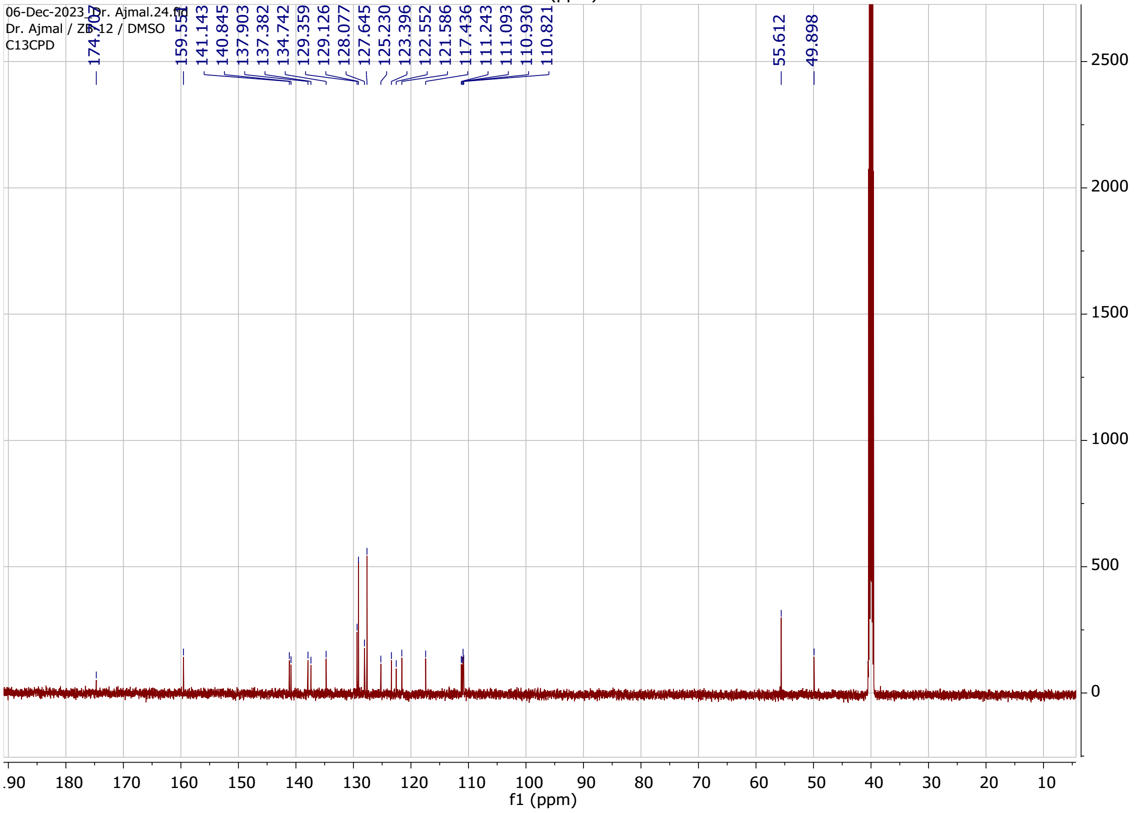

1H NMR and 13C NMR Spectra of 5m

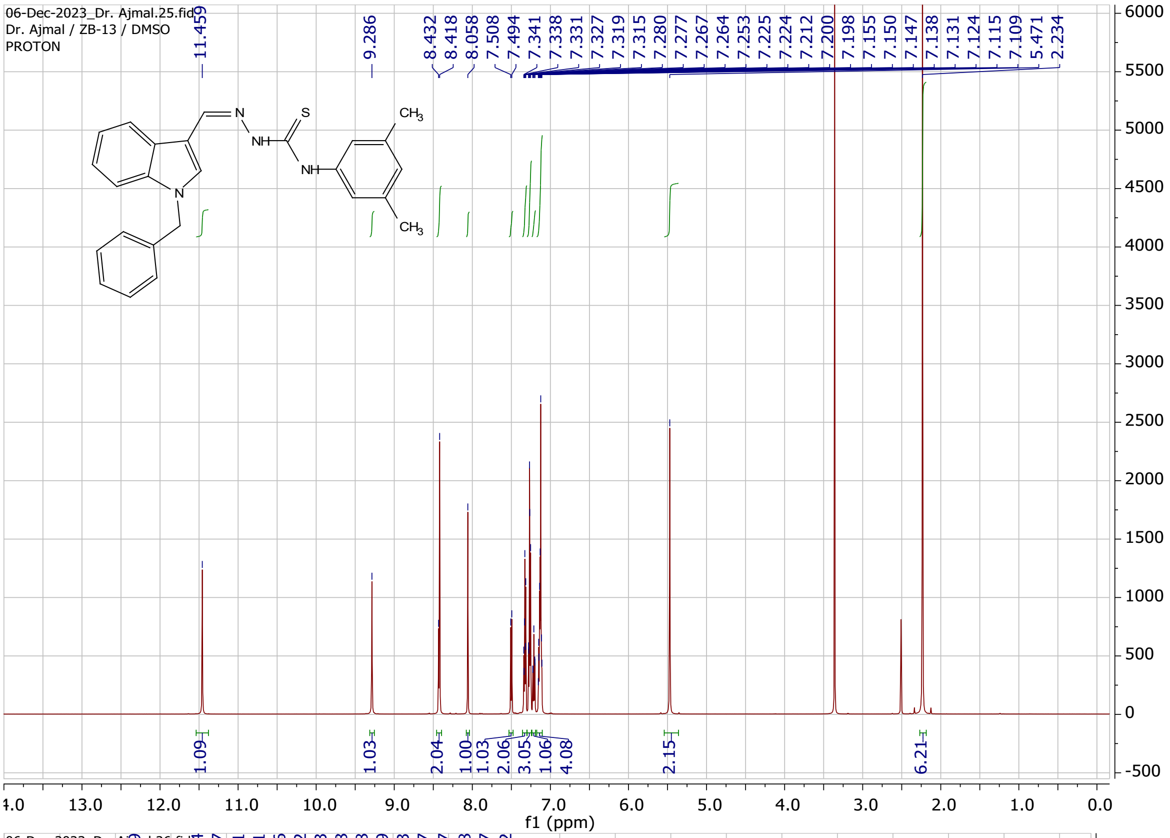

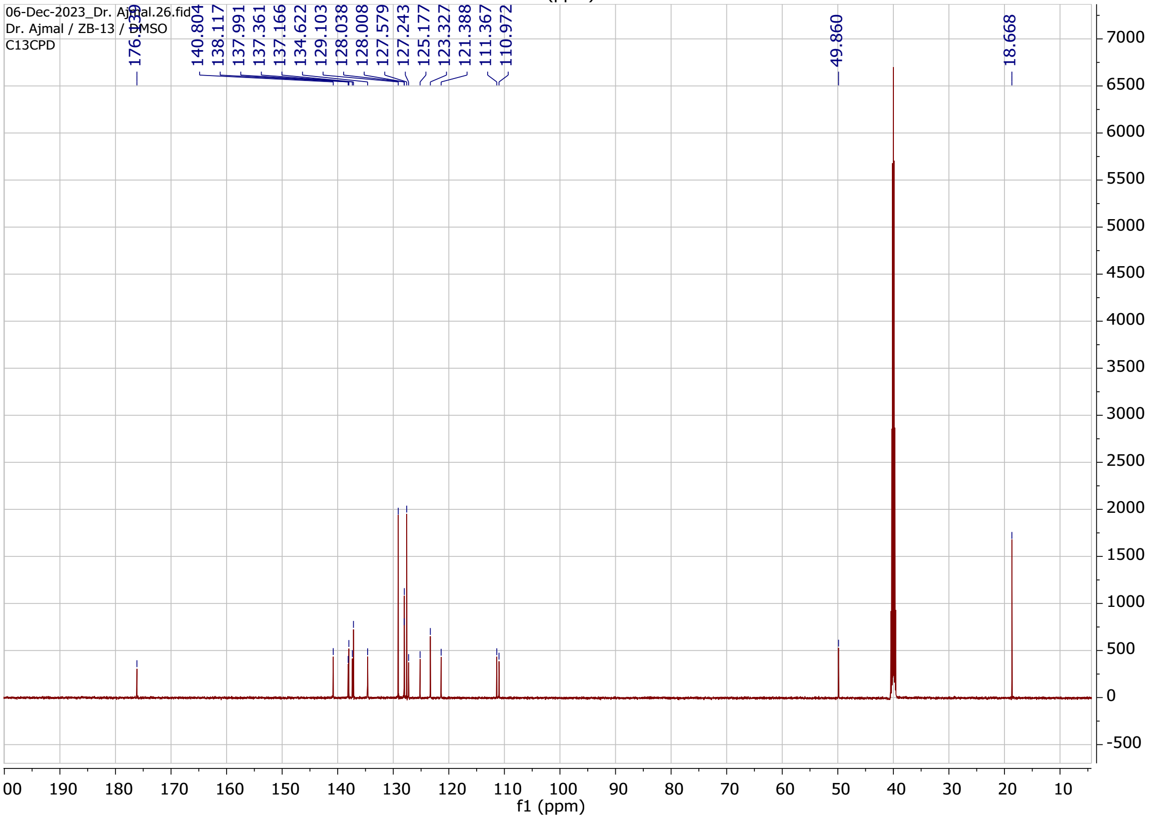

1H NMR and 13C NMR Spectra of 5n

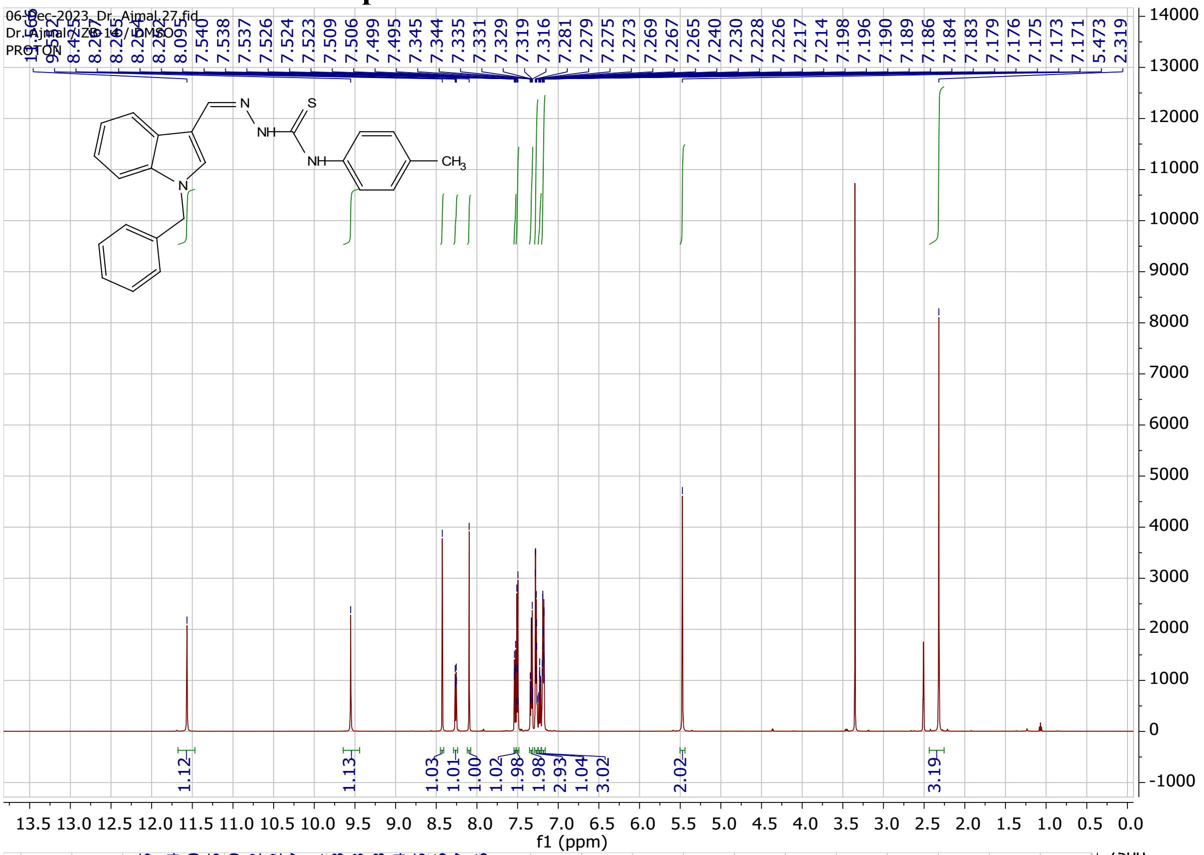

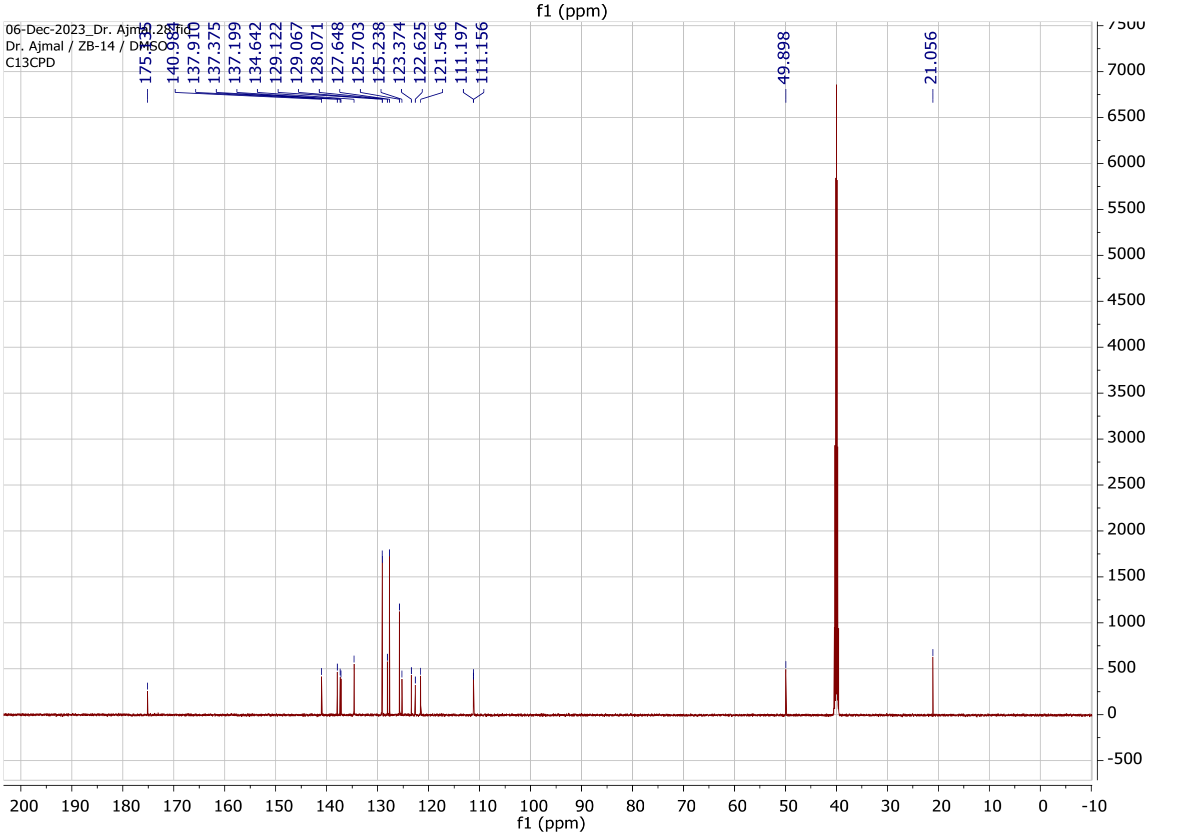

^{1}H NMR and ^{13}C NMR Spectra of 5o

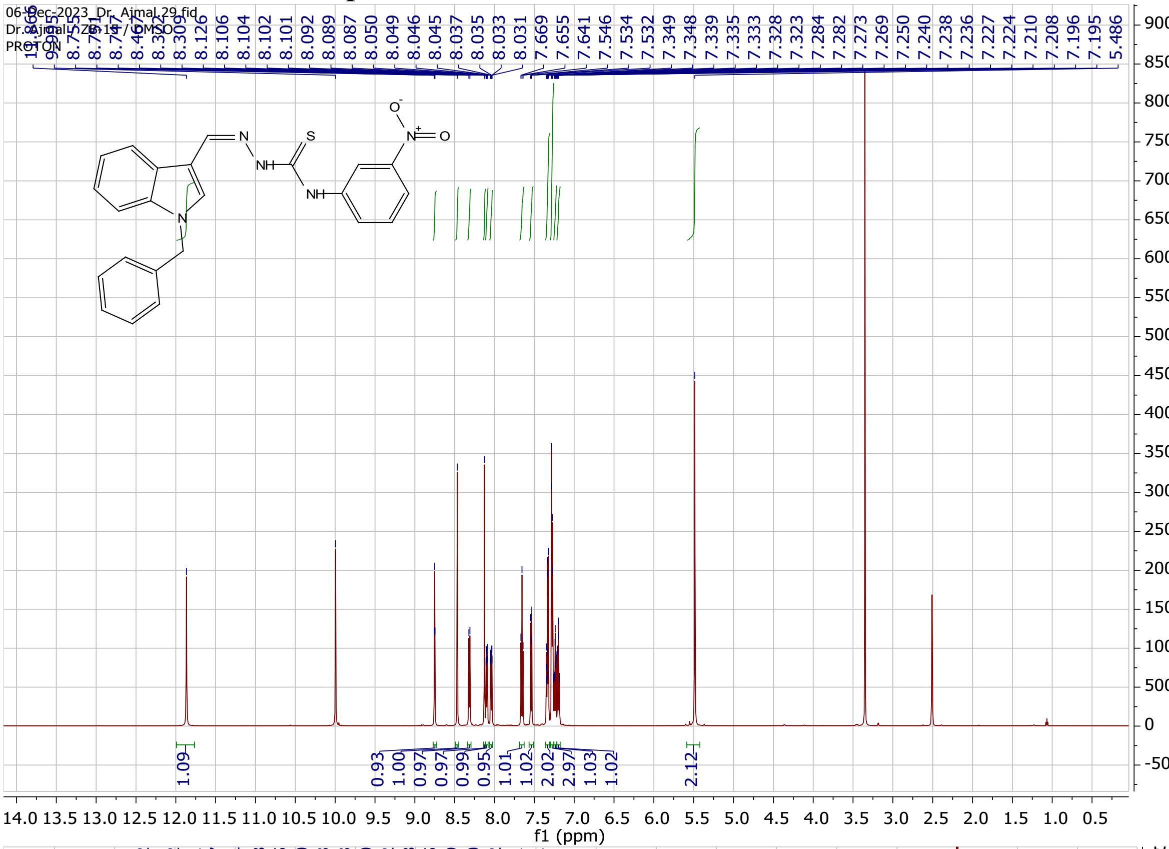

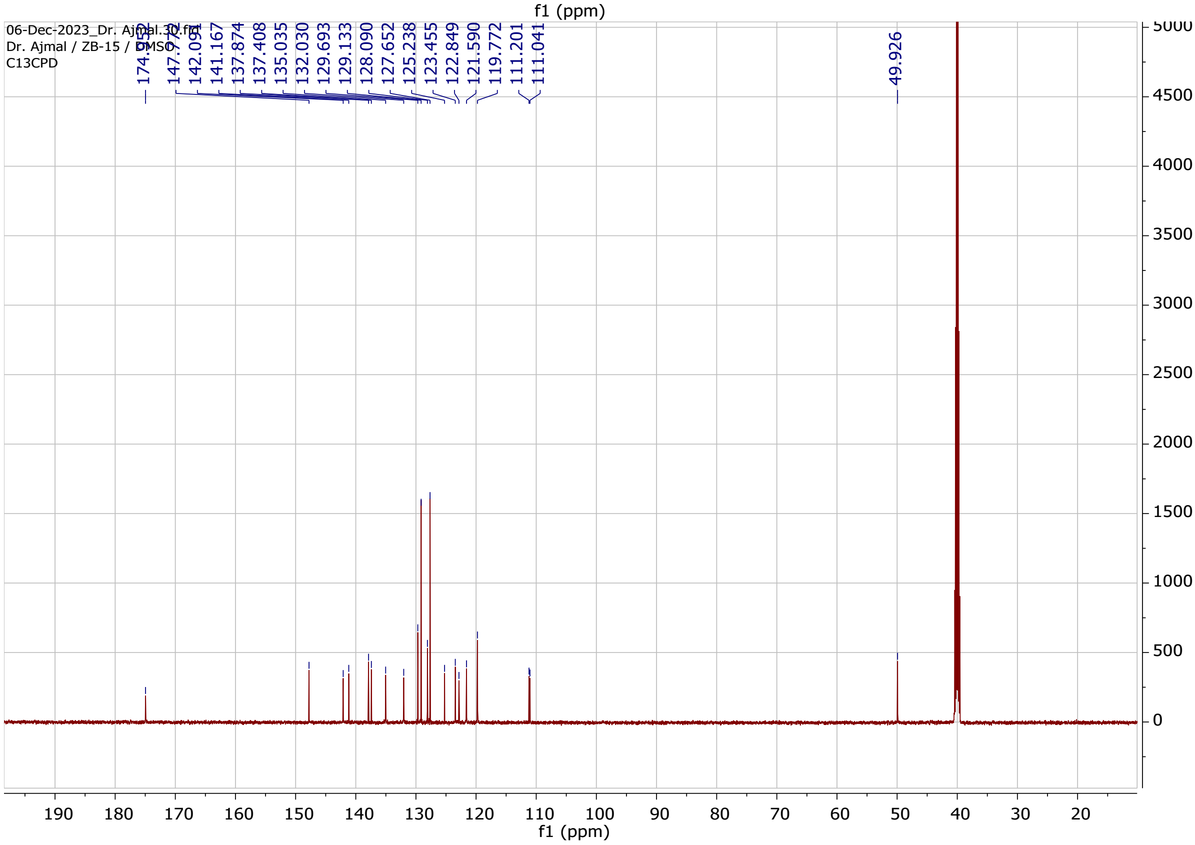

1H NMR and 13C NMR Spectra of 5p

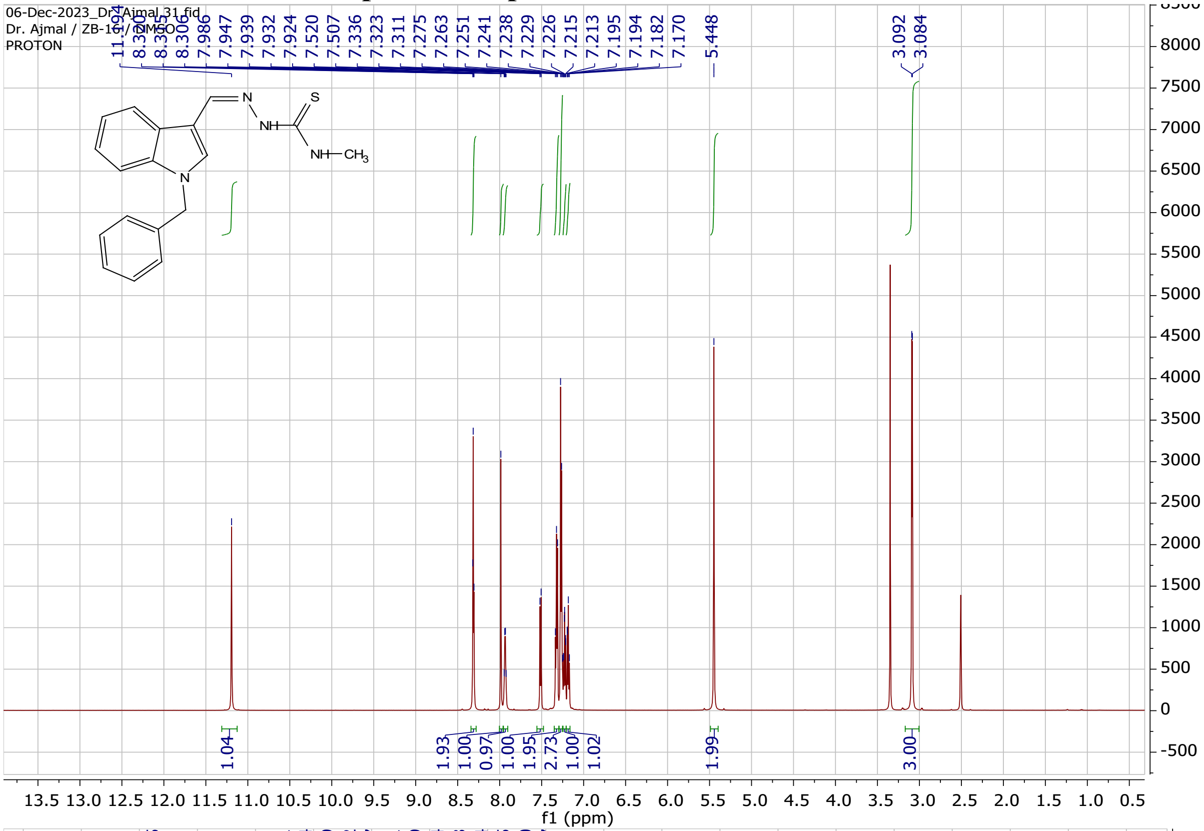

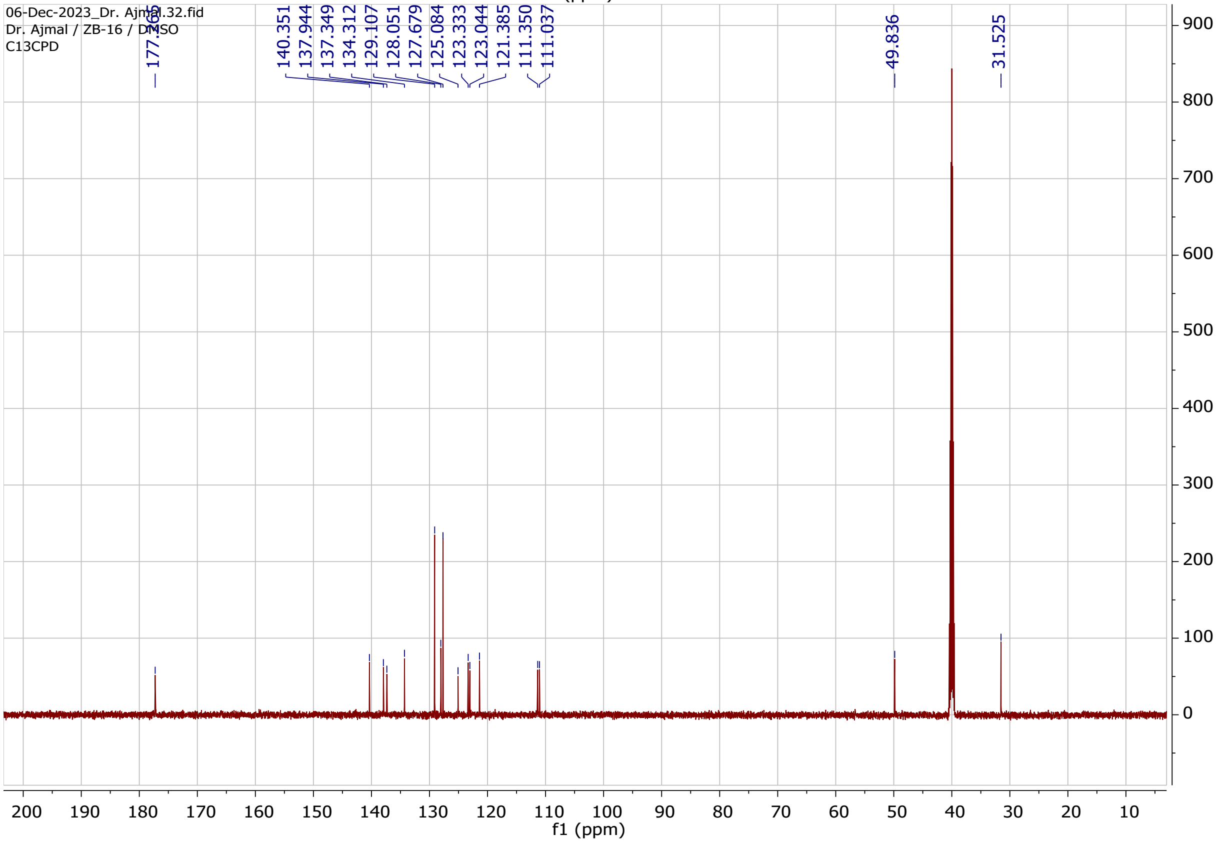

1H NMR and 13C NMR Spectra of 5q

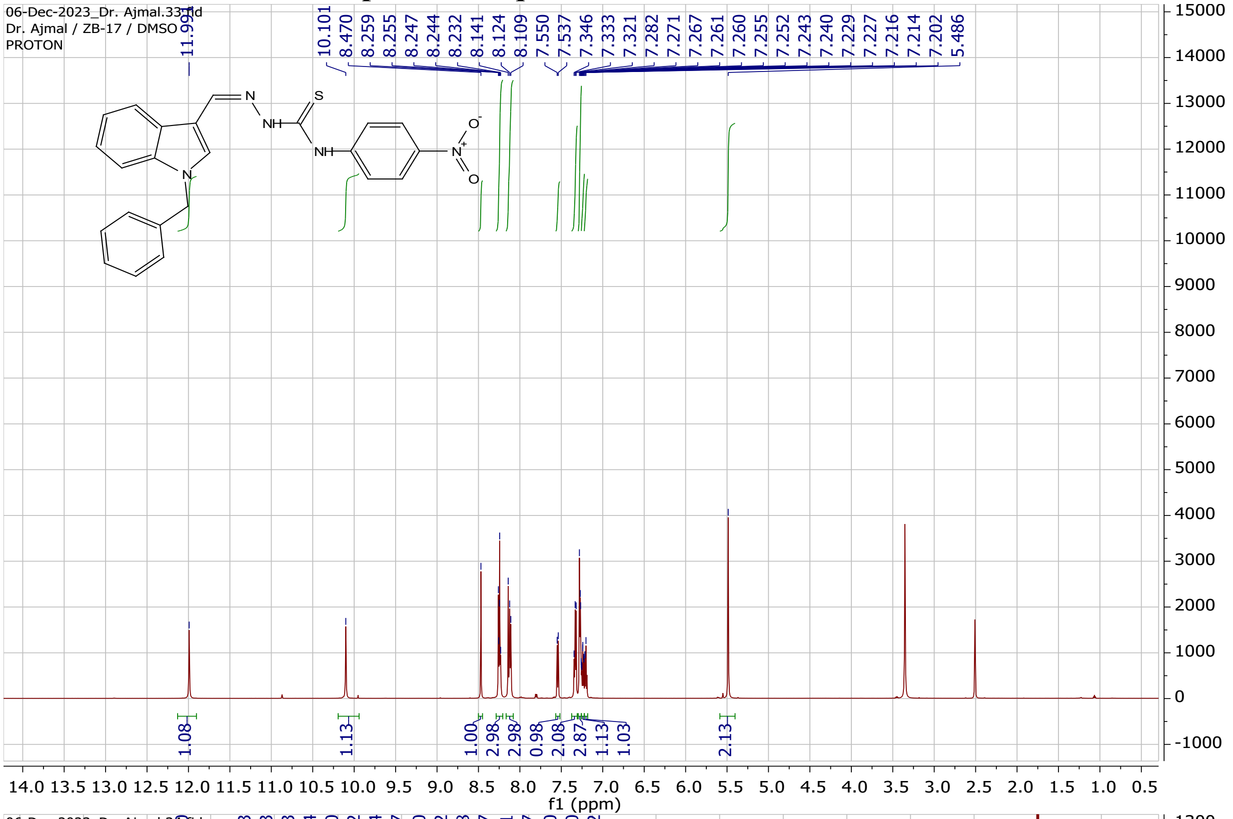

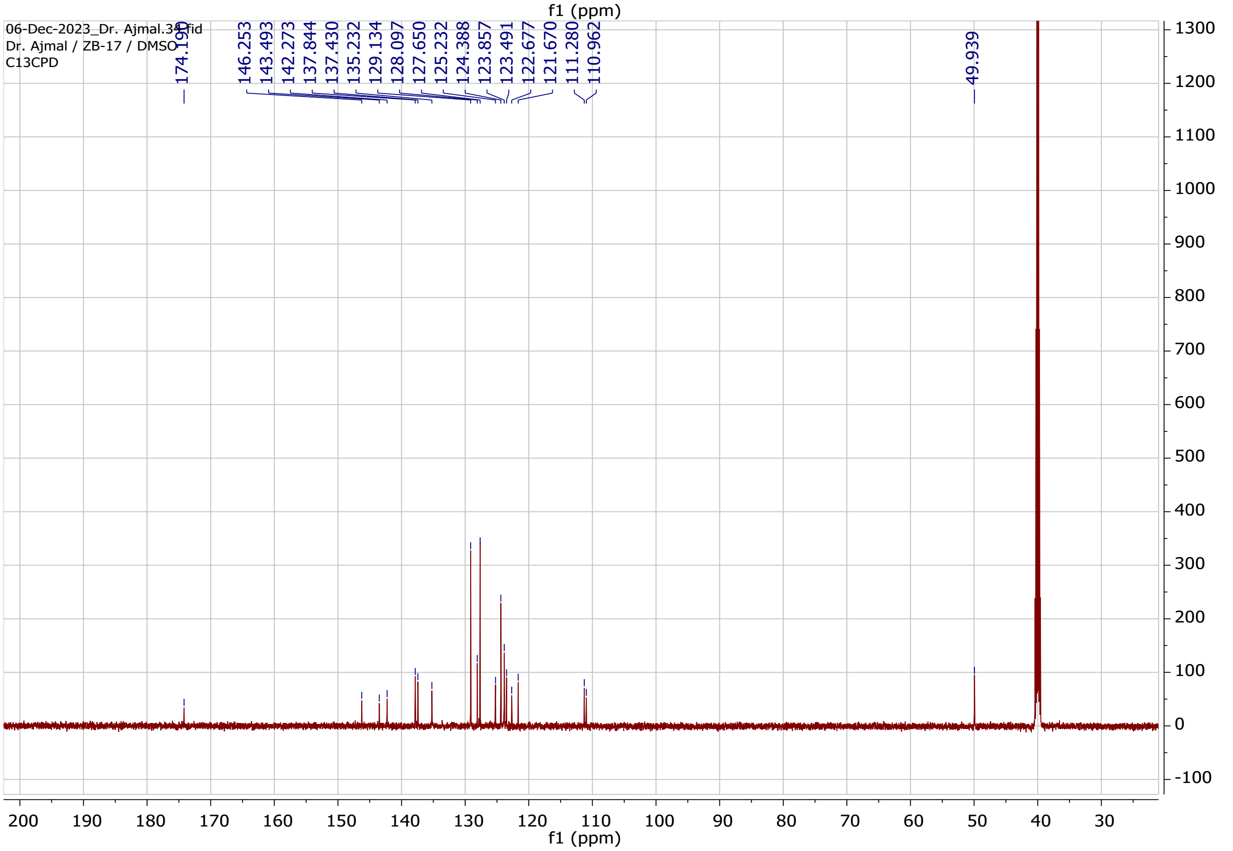

1H NMR and 13C NMR Spectra of 5r

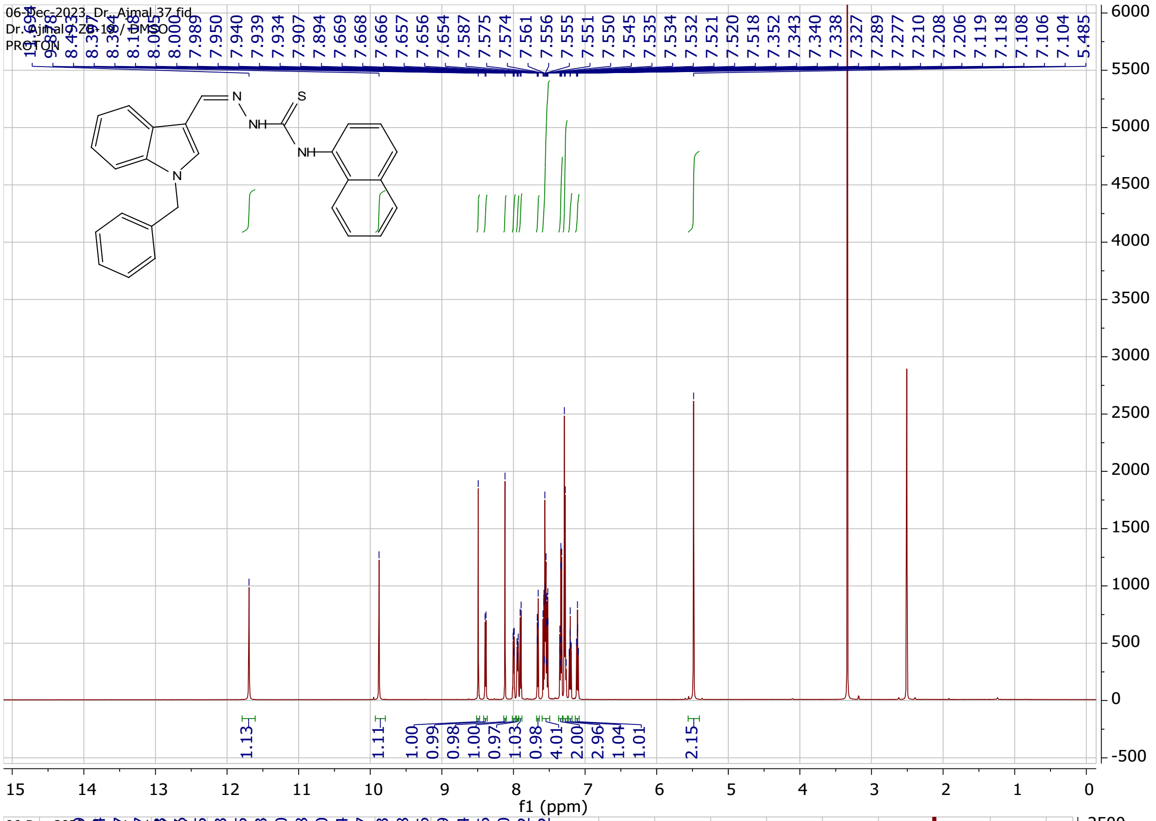

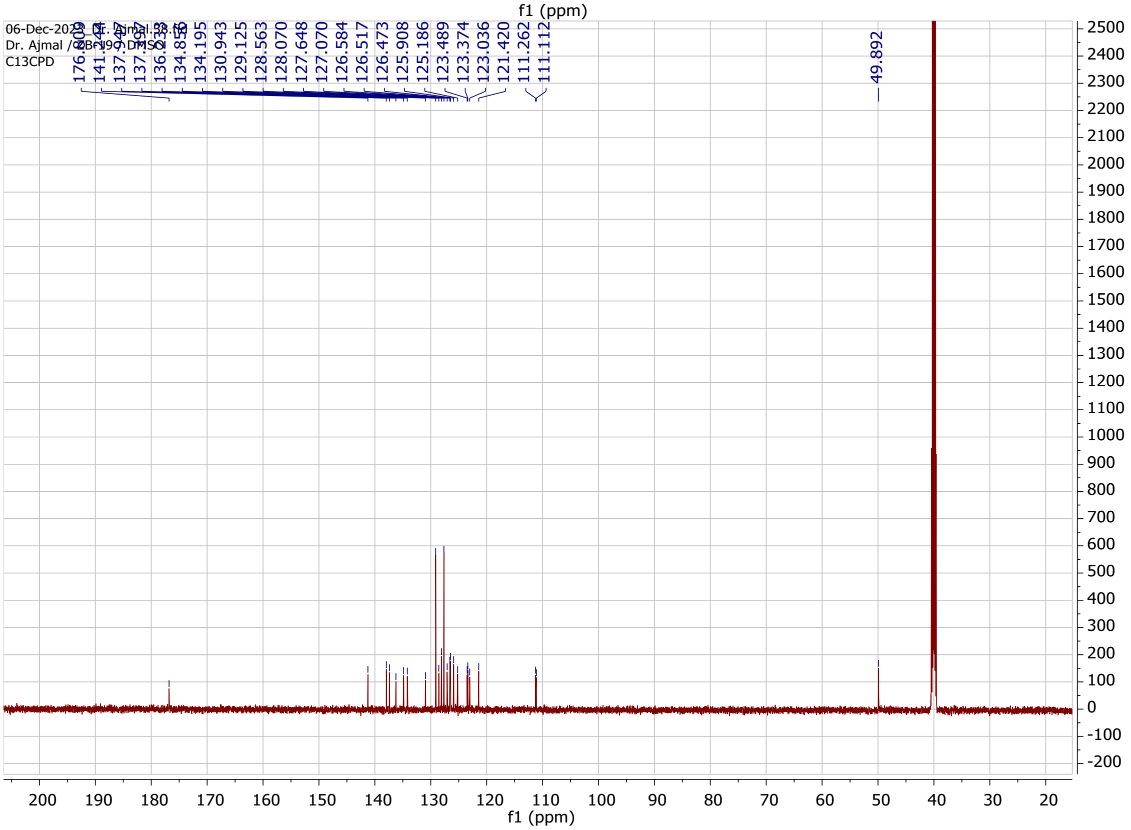

HPLC chromatogram of 5a

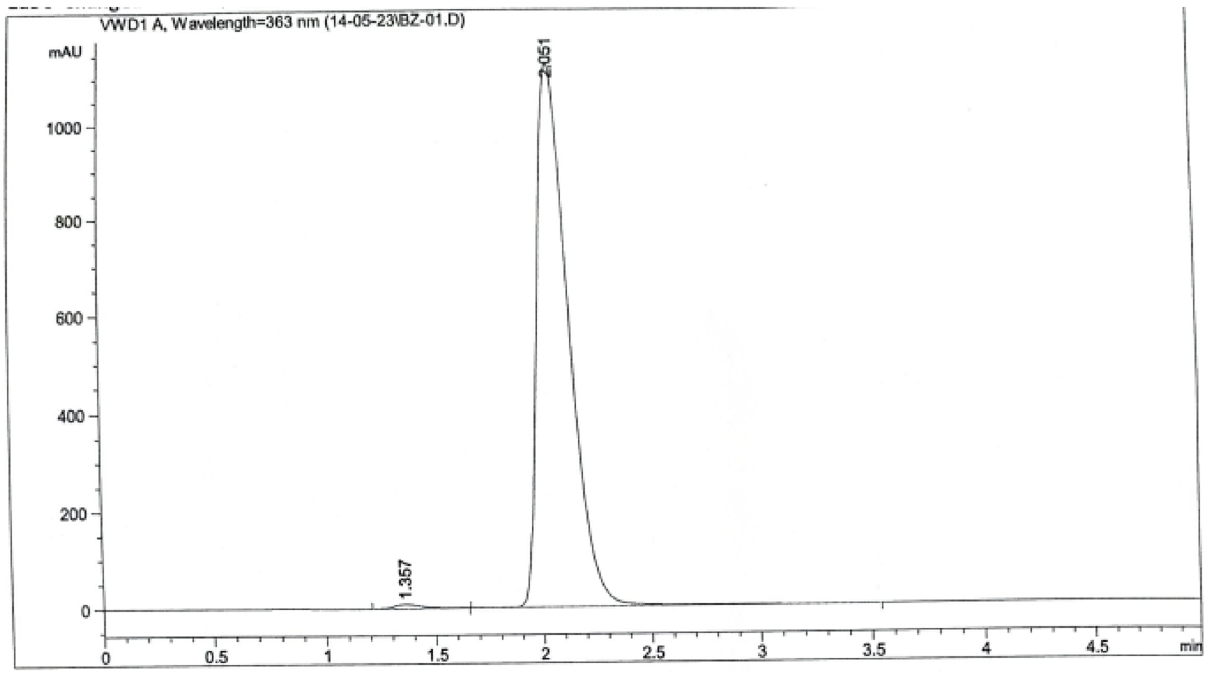

HPLC chromatogram of 5b

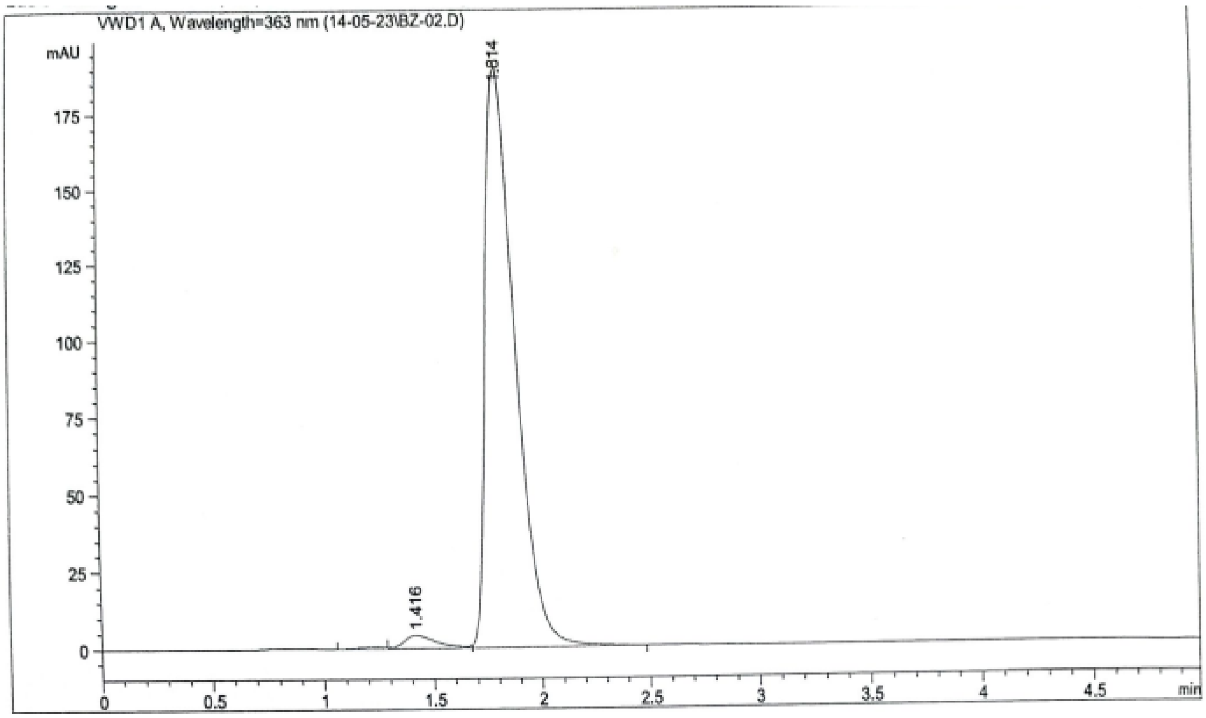

HPLC chromatogram of 5c

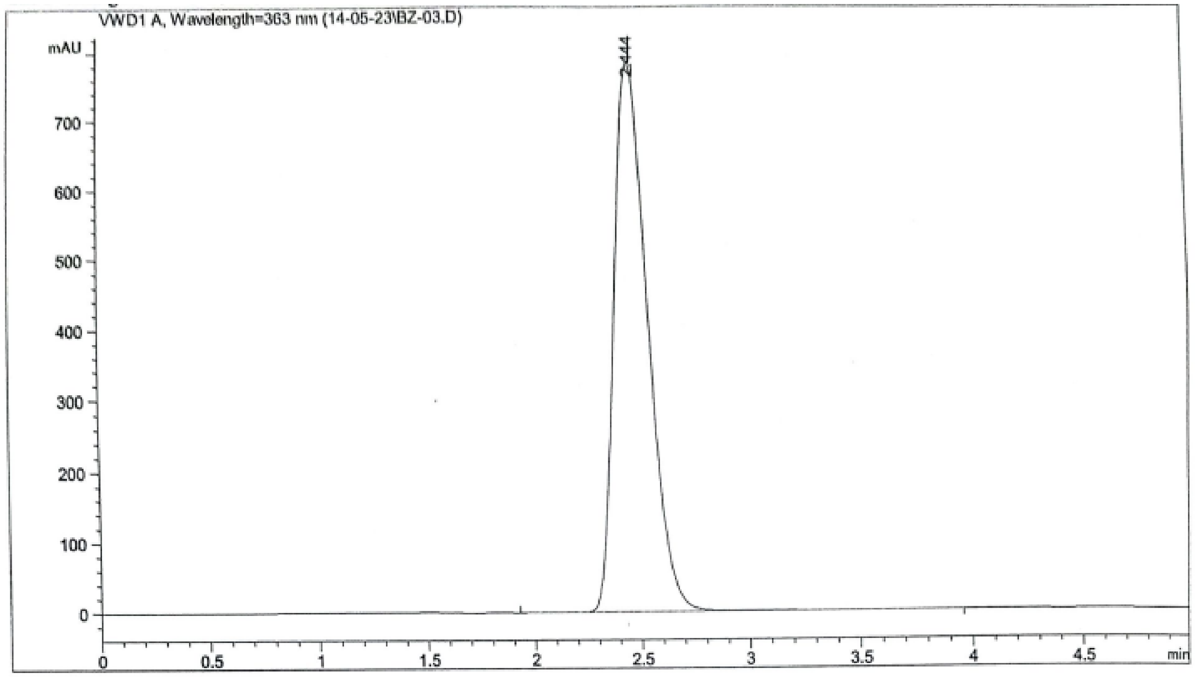

HPLC chromatogram of 5d

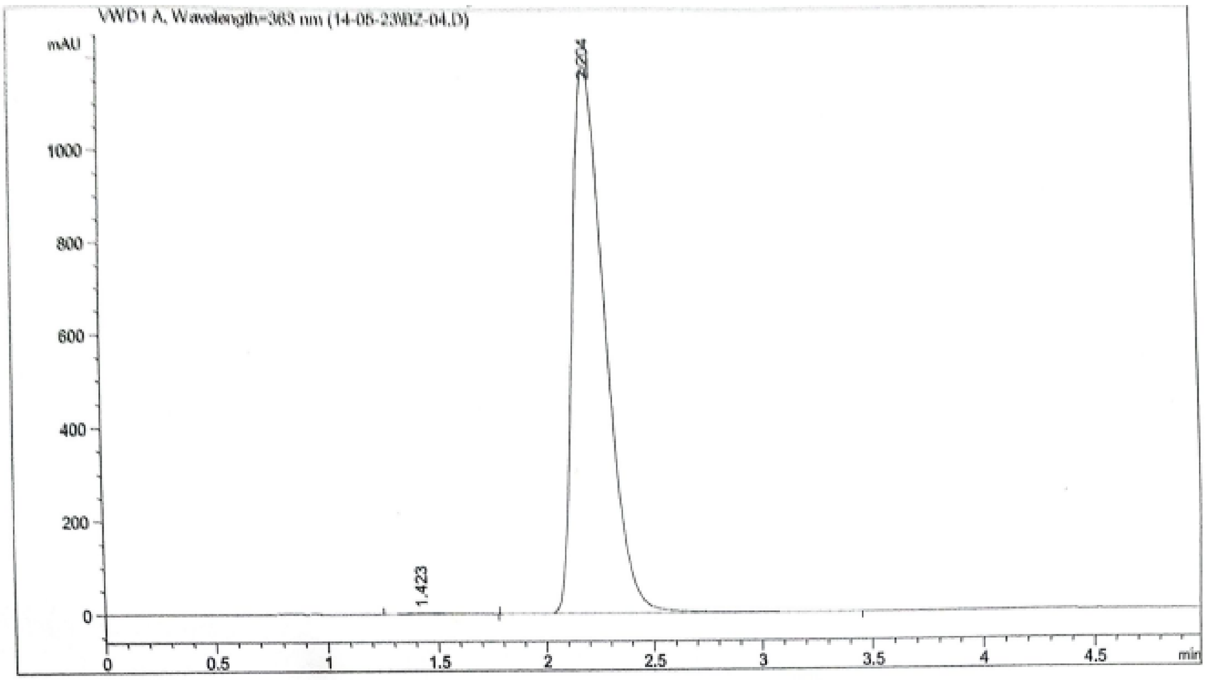

HPLC chromatogram of 5e

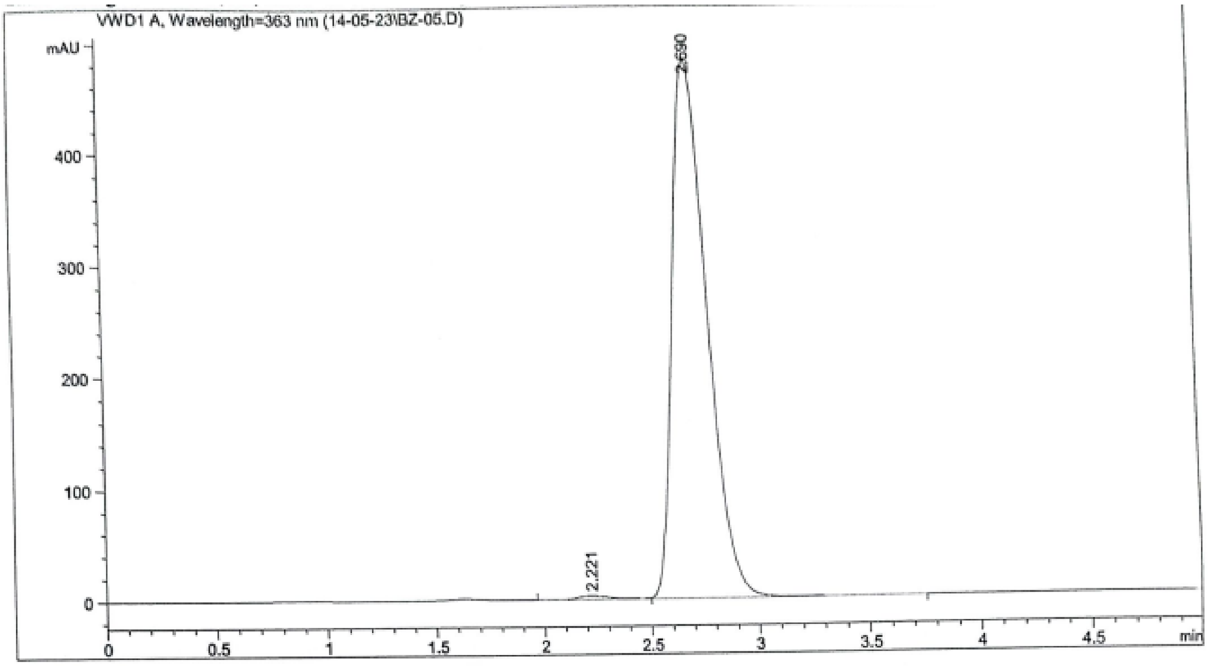

HPLC chromatogram of 5f

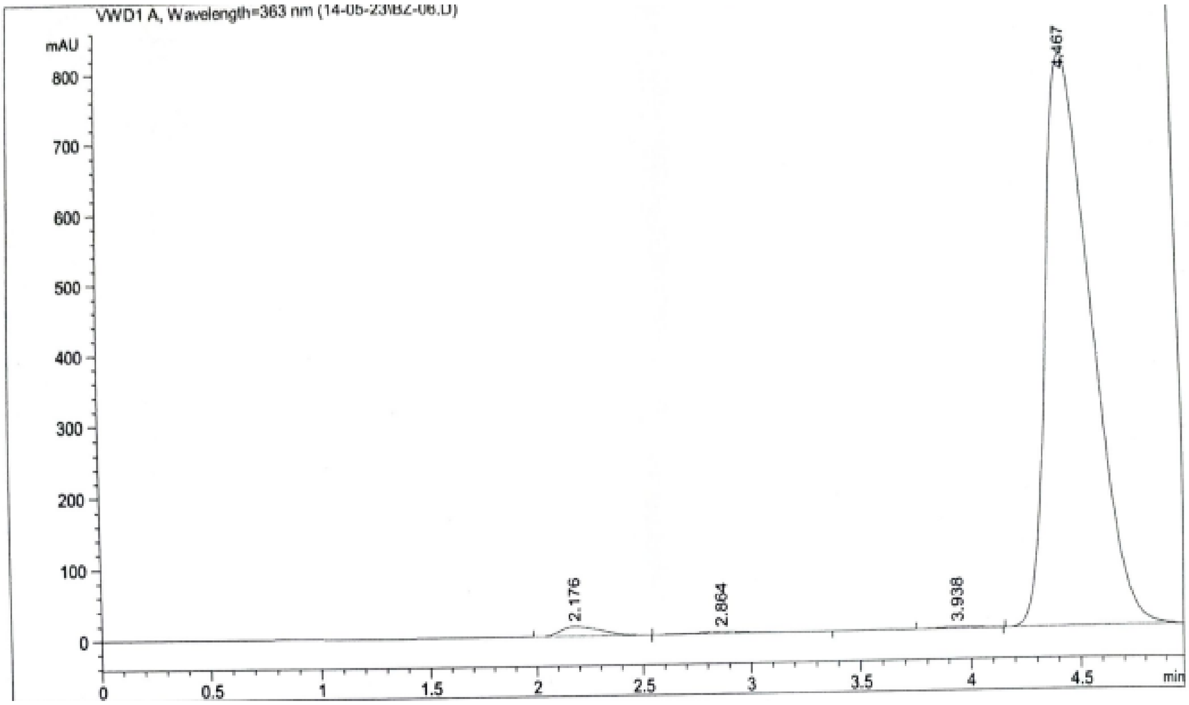

HPLC chromatogram of 5g

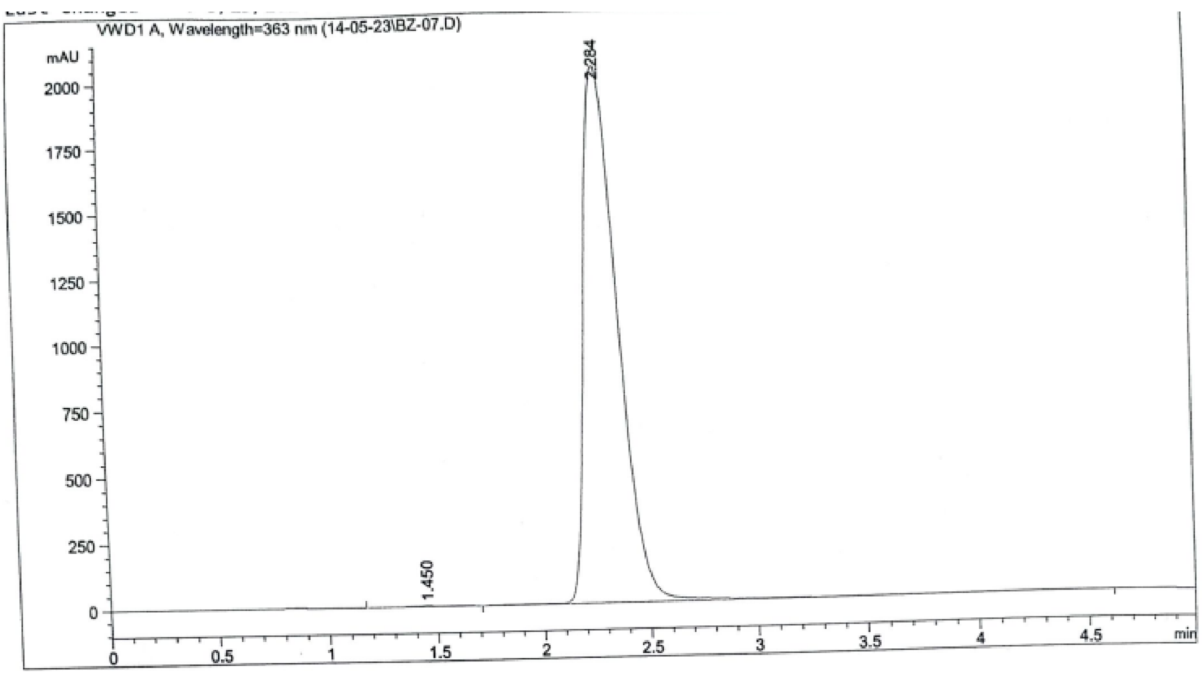

HPLC chromatogram of 5h

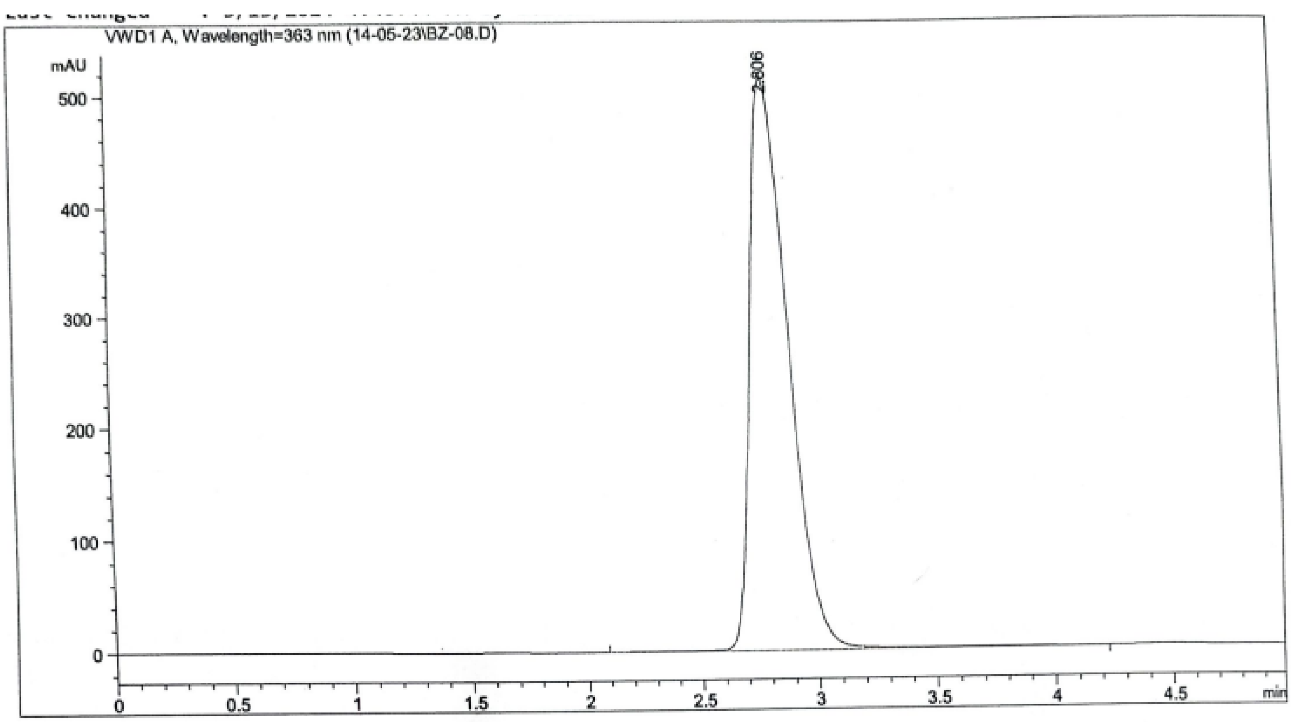

HPLC chromatogram of 5i

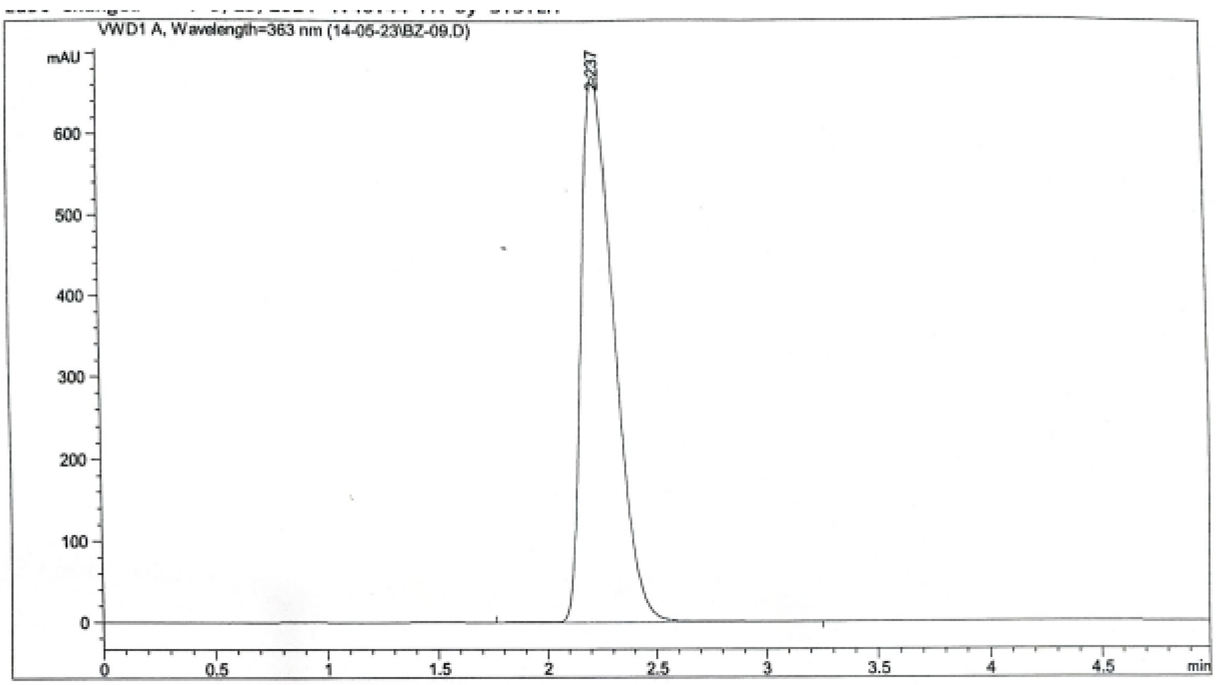

HPLC chromatogram of 5j

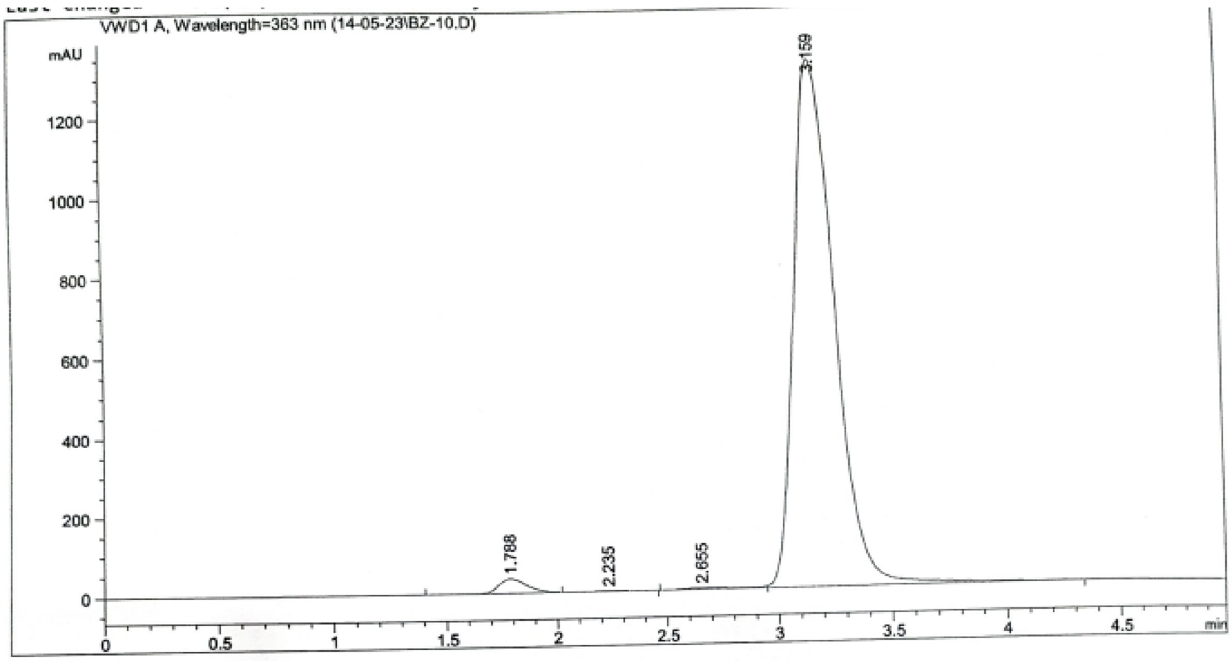

HPLC chromatogram of 5k

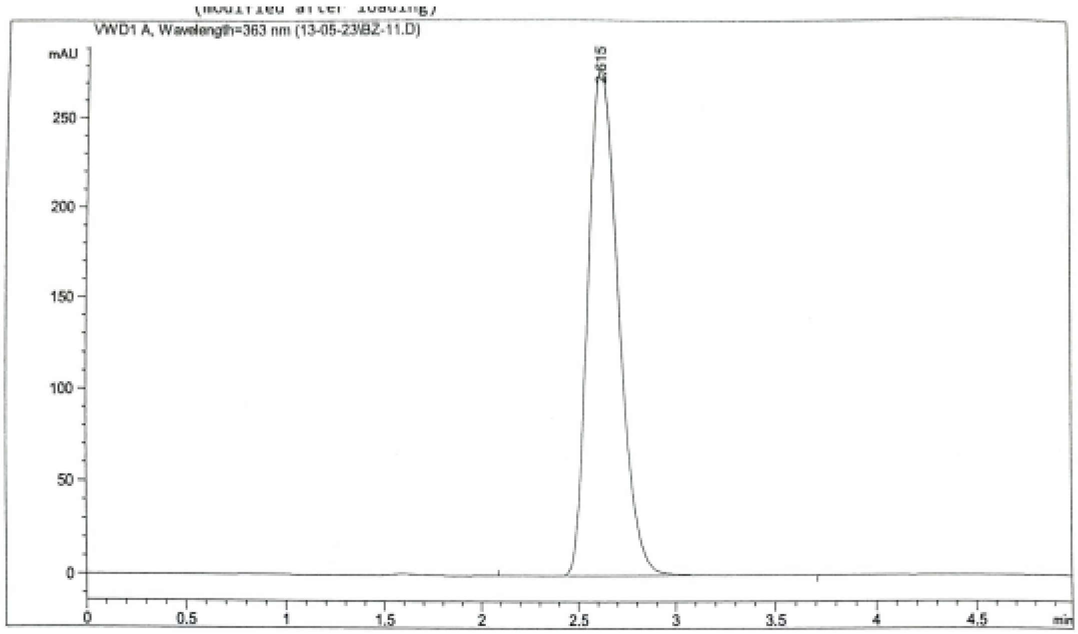

HPLC chromatogram of 5l

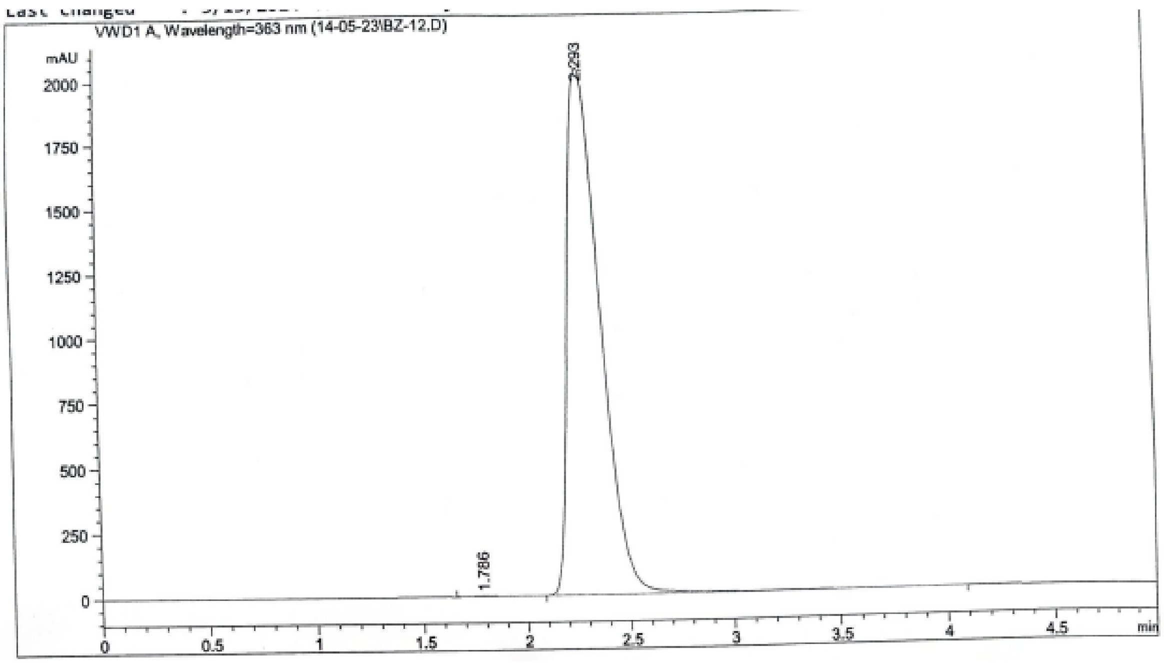

HPLC chromatogram of 5m

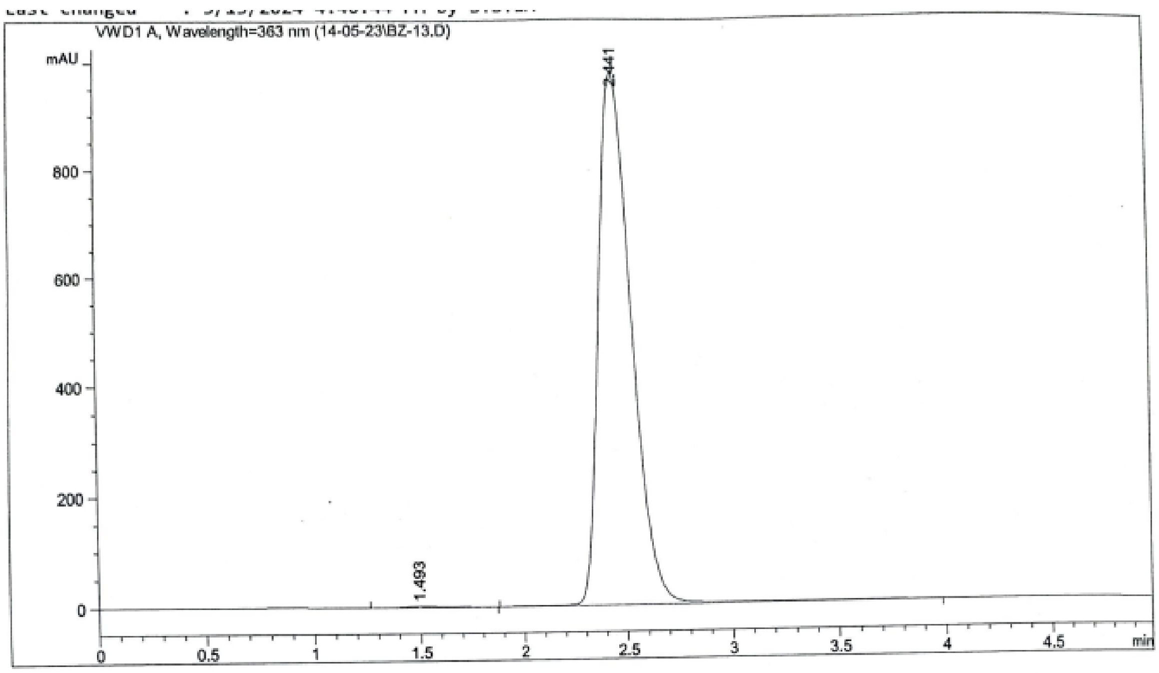

HPLC chromatogram of 5n

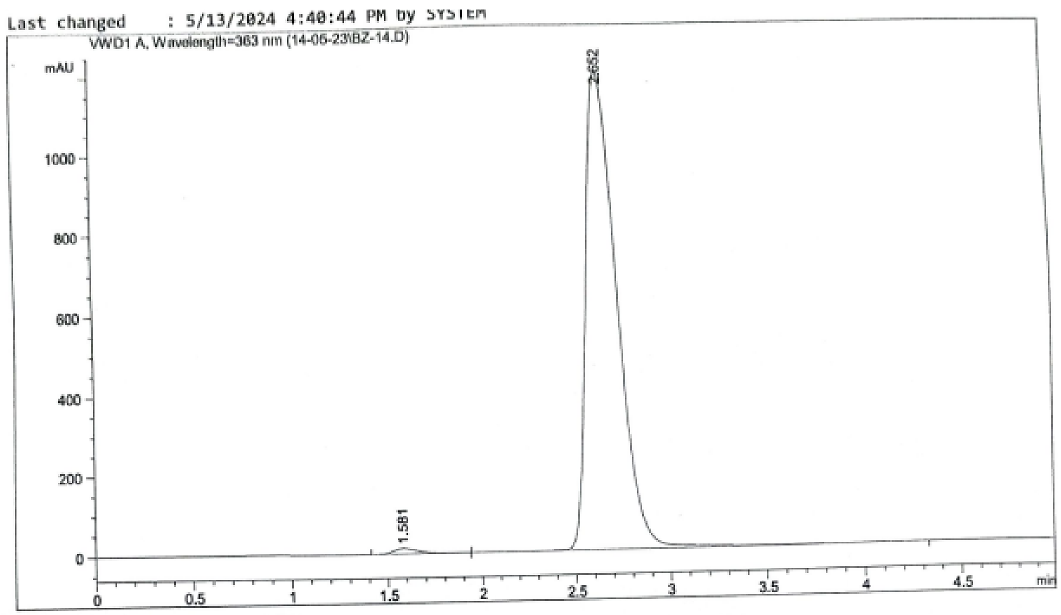

HPLC chromatogram of 5o

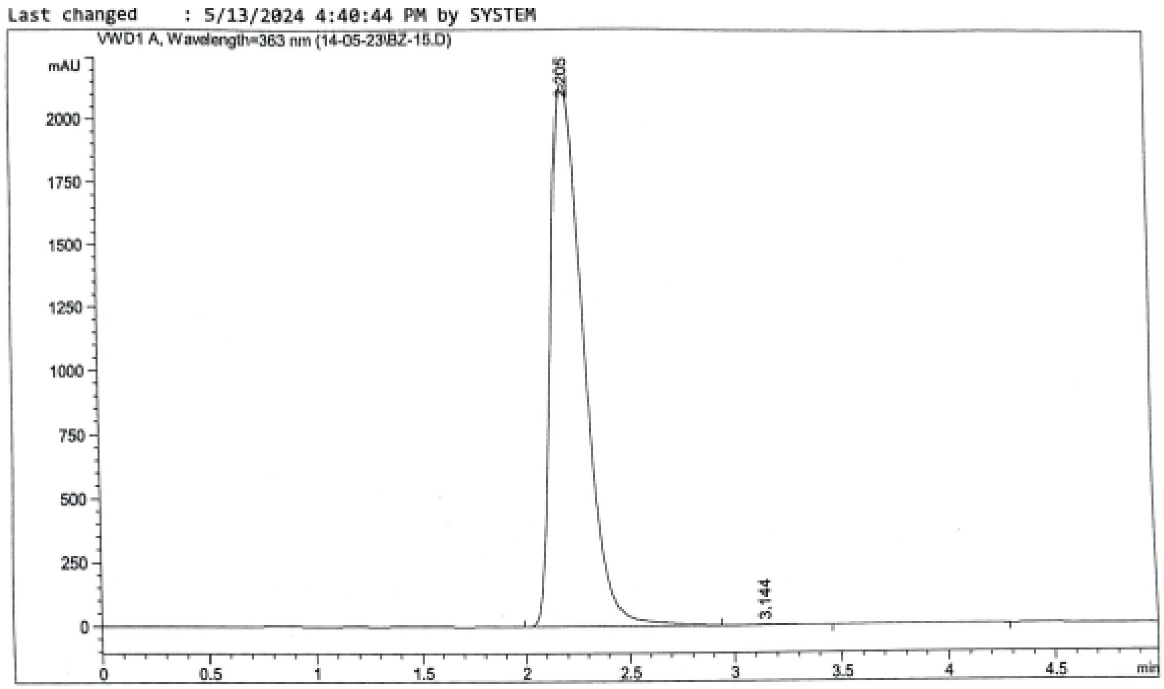

HPLC chromatogram of 5p

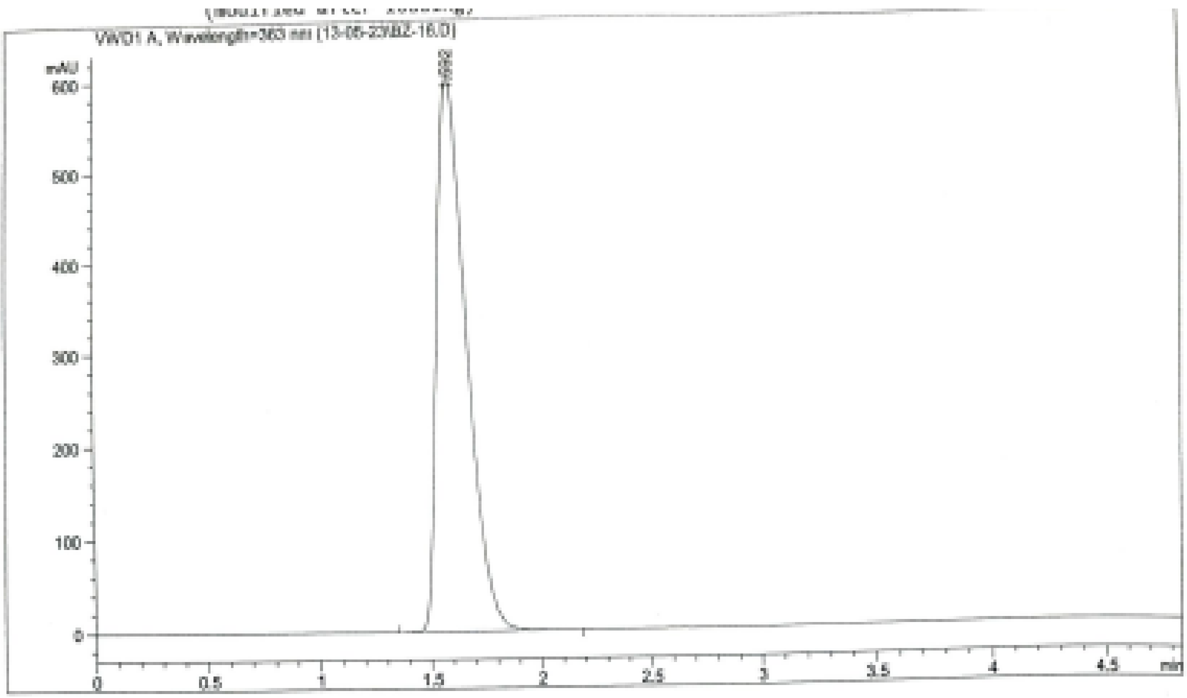

HPLC chromatogram of 5q

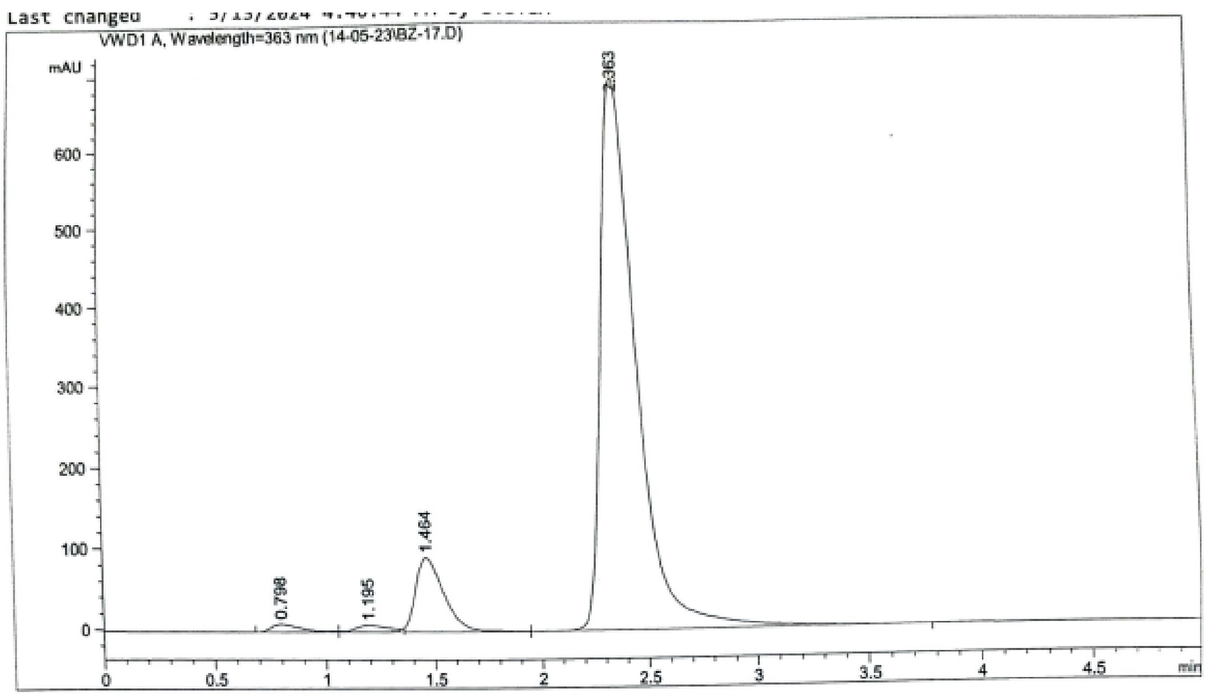

HPLC chromatogram of 5r

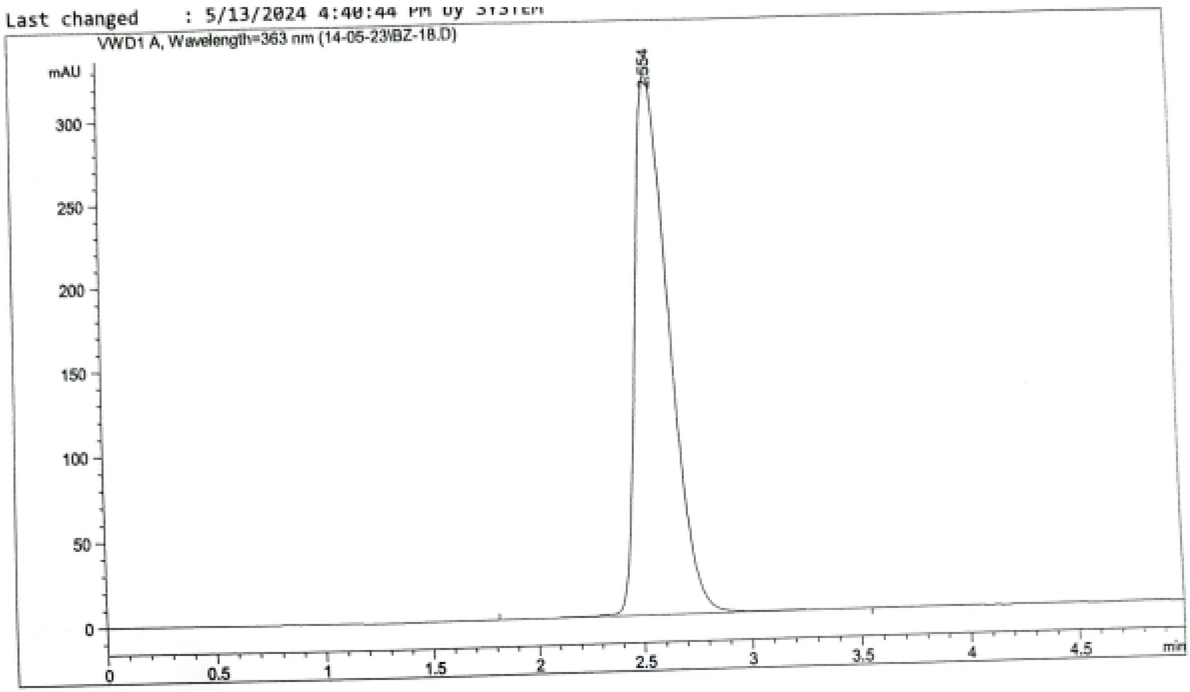

Mass spectrum of 5a

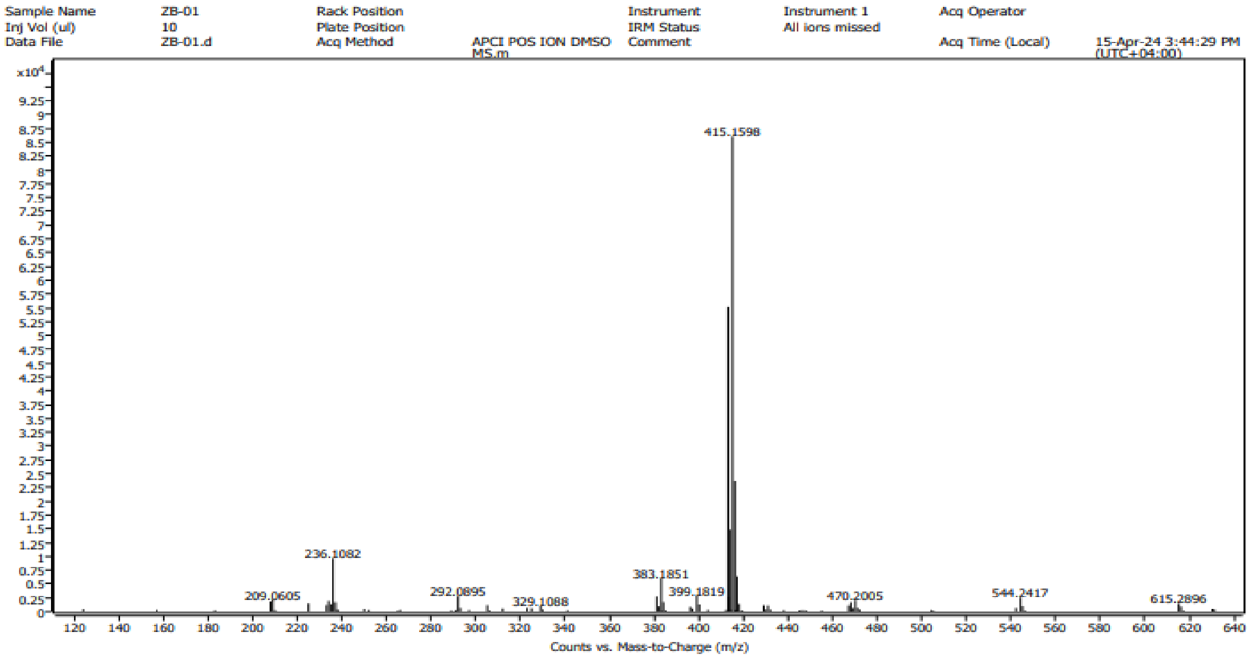

Mass spectrum of 5b

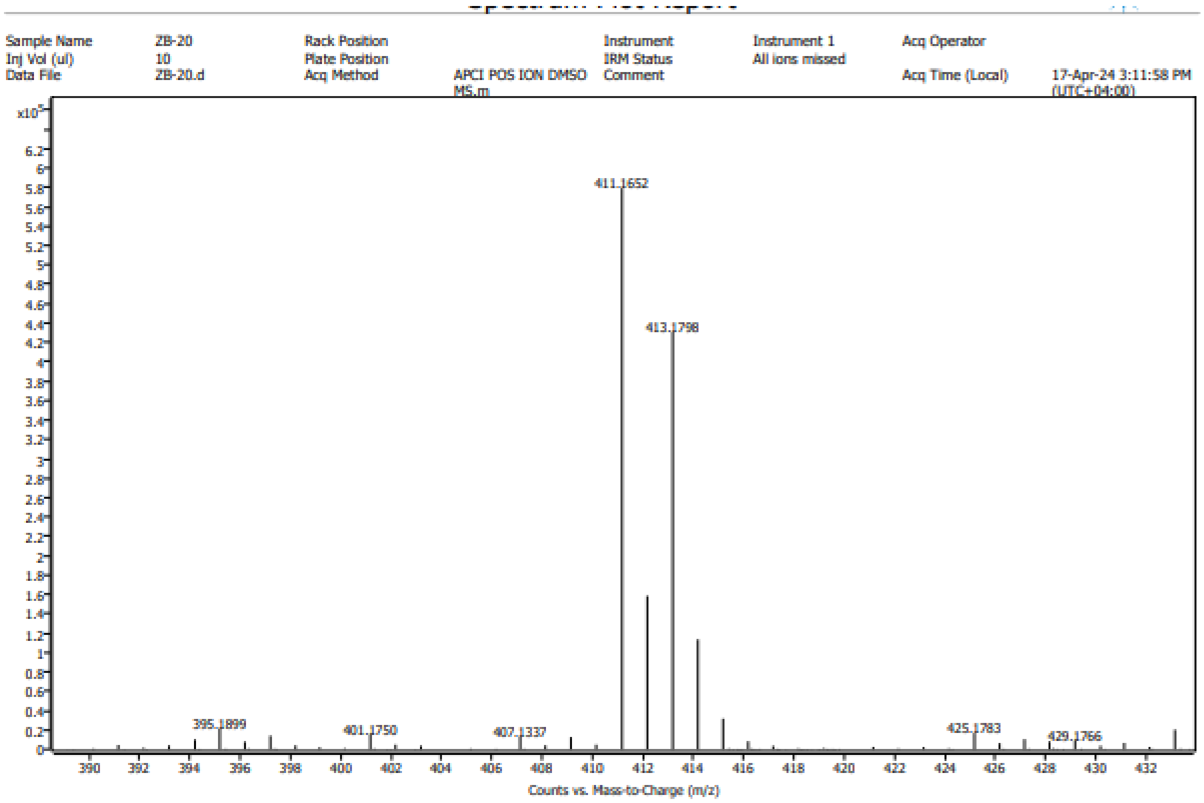

Mass spectrum of 5c

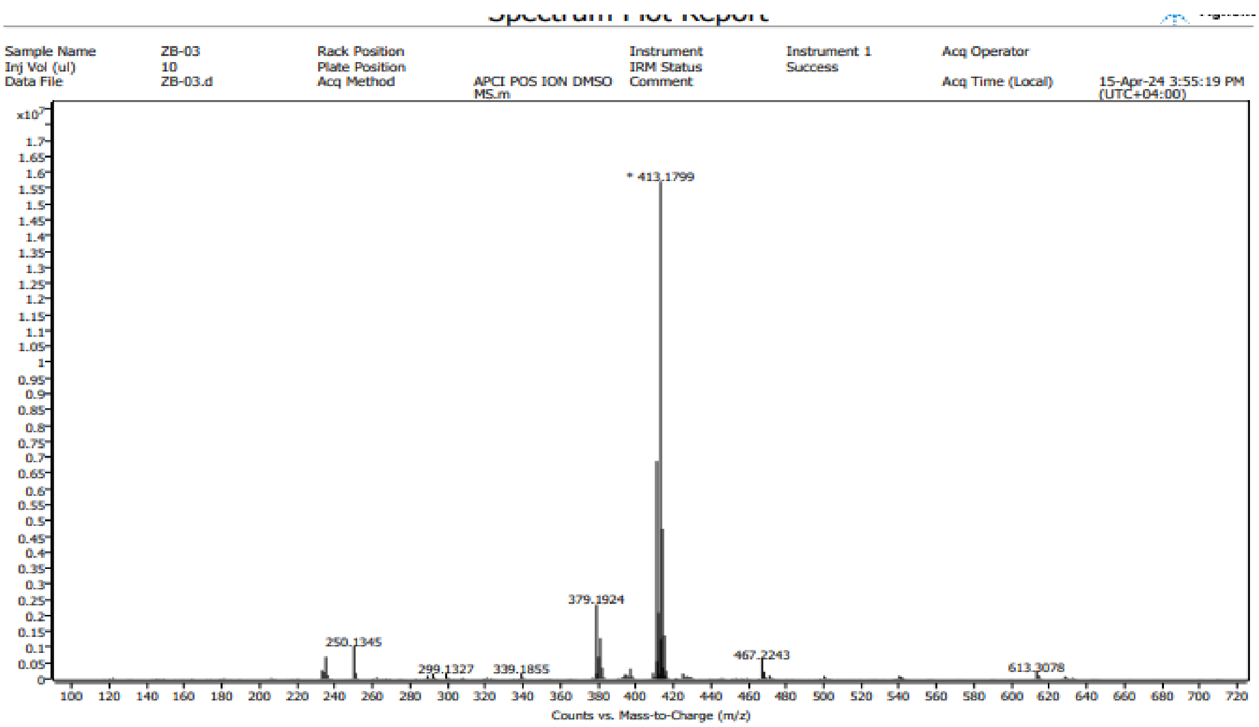

Mass spectrum of 5d

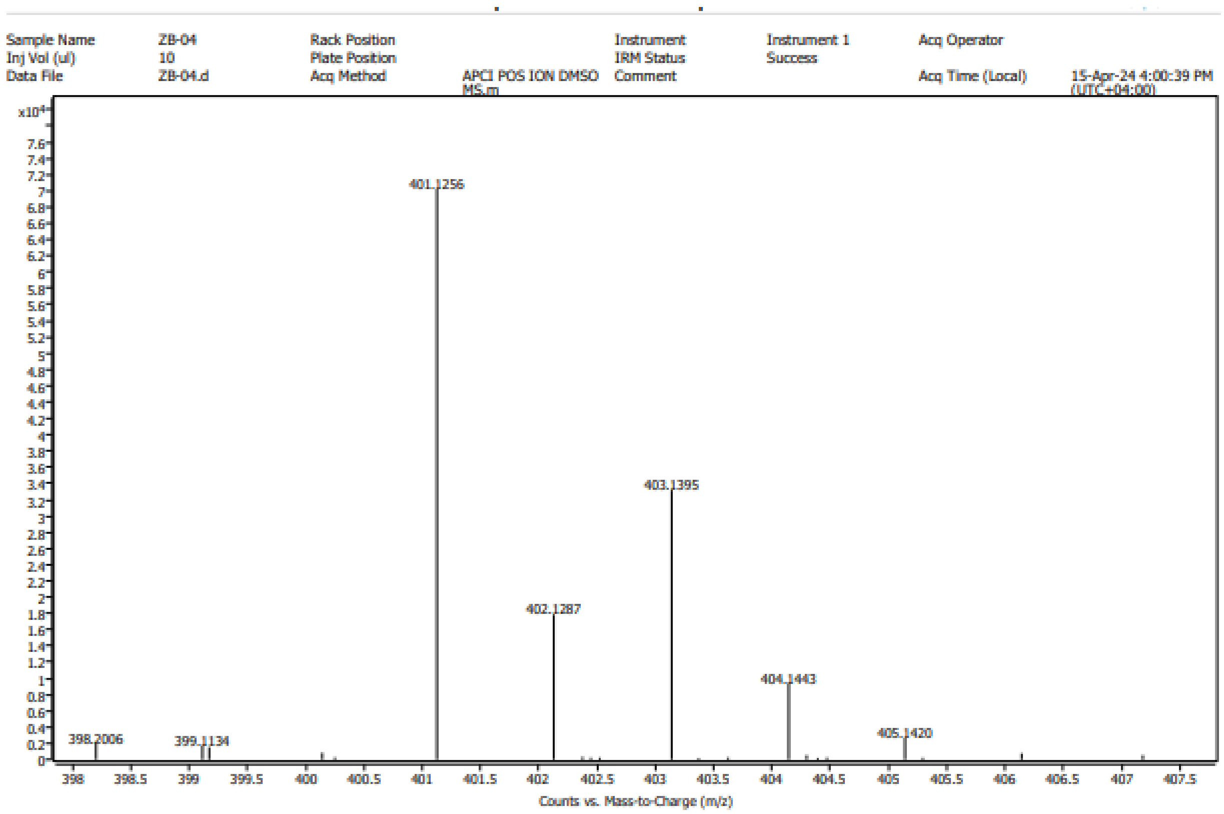

Mass spectrum of 5e

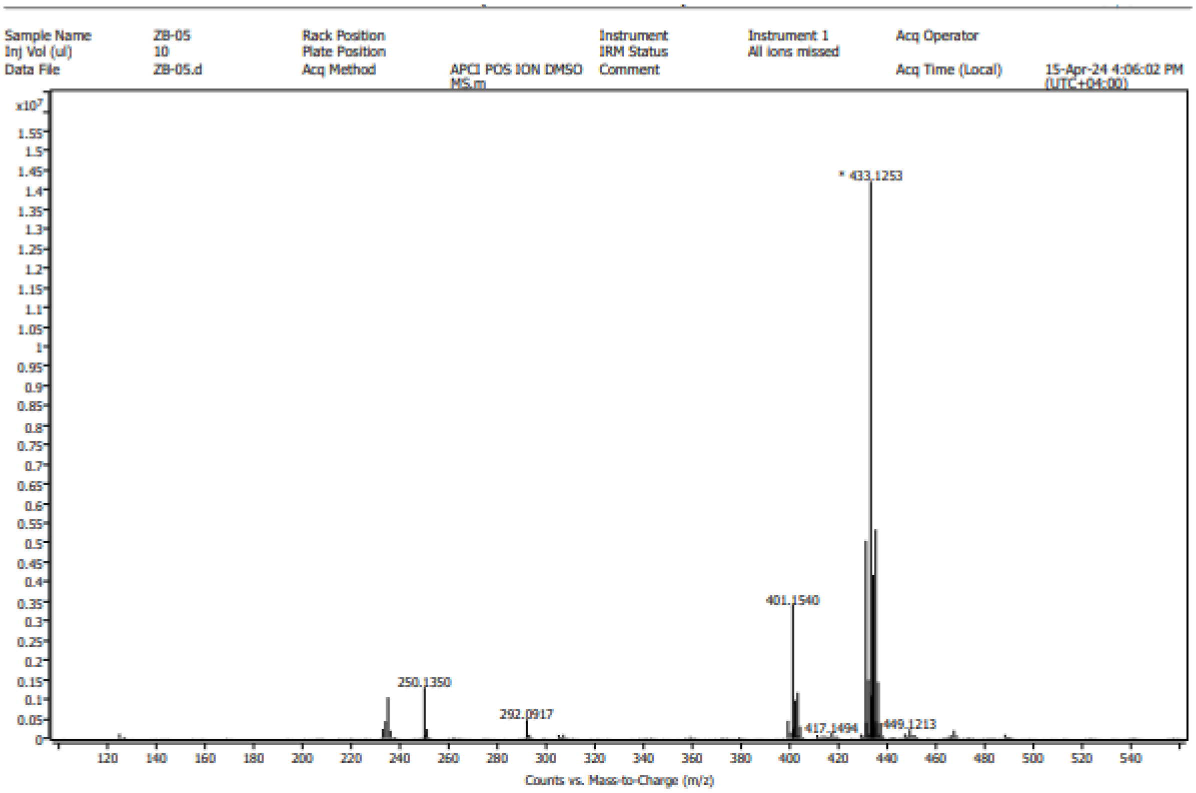

Mass spectrum of 5f

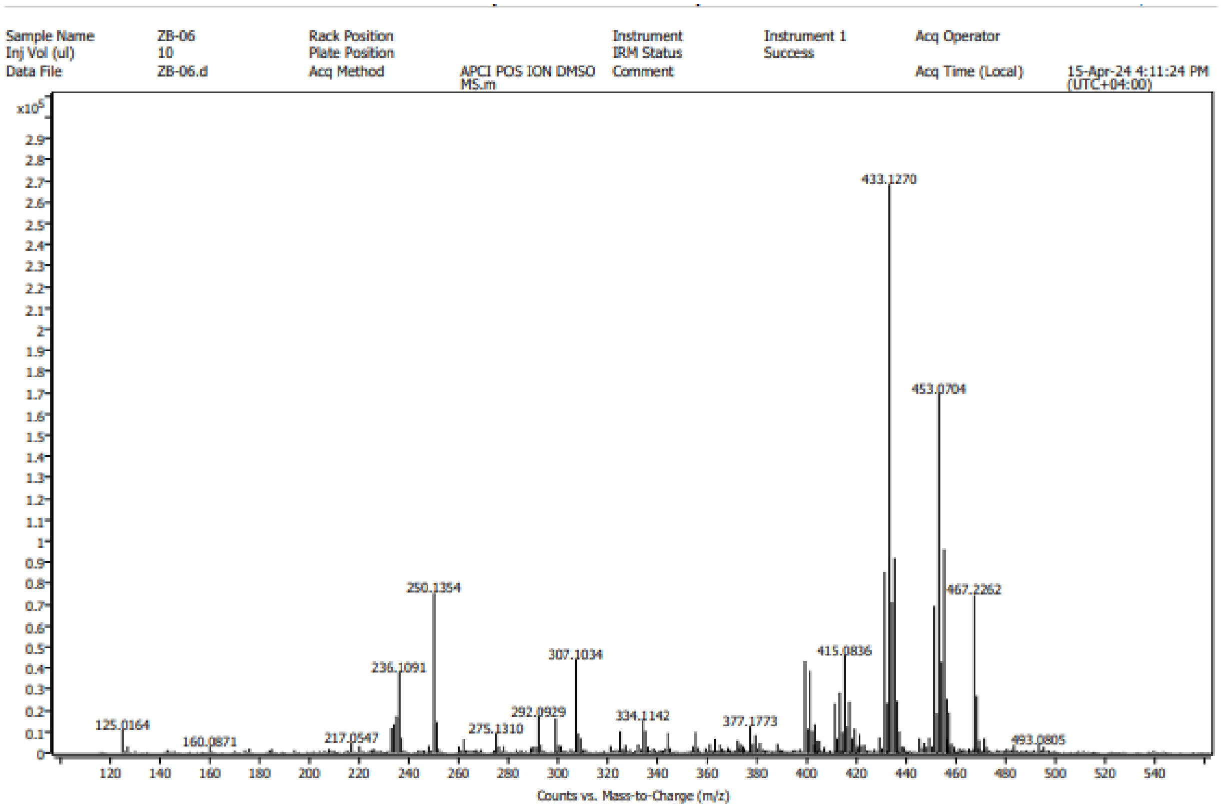

Mass spectrum of 5g

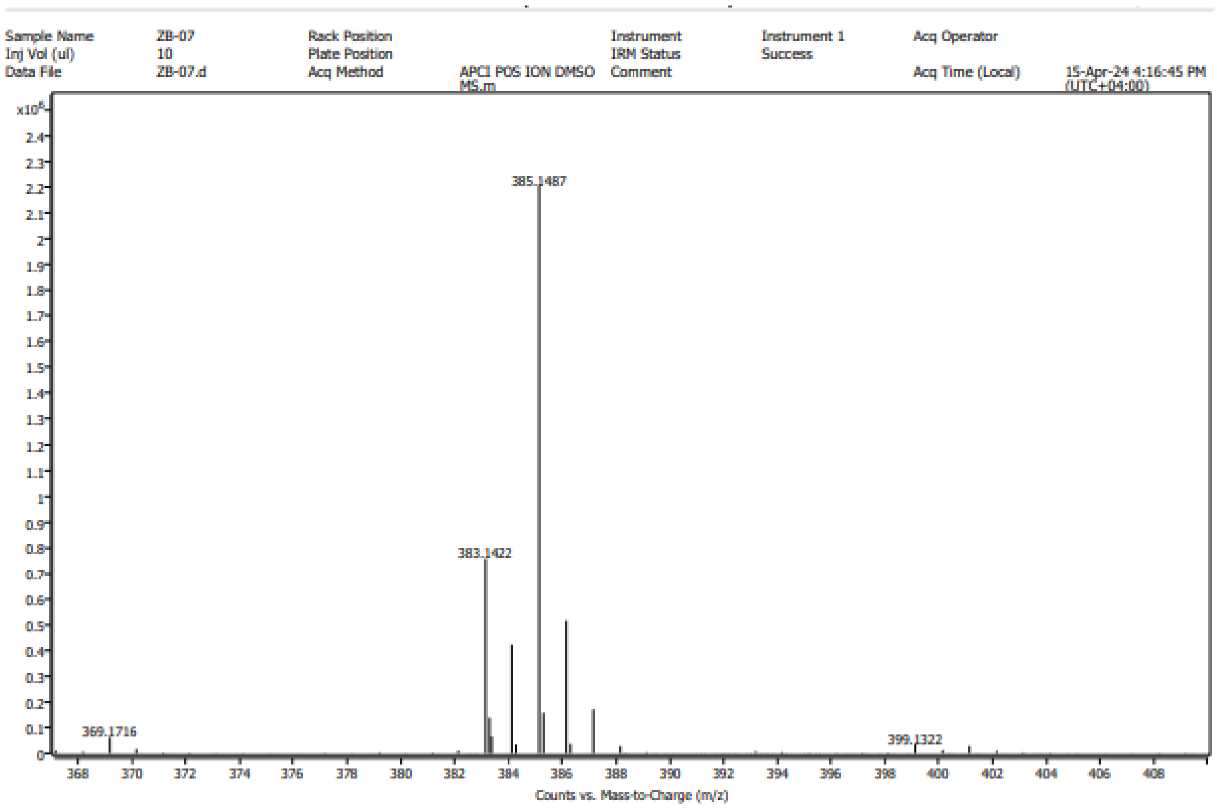

Mass spectrum of 5h

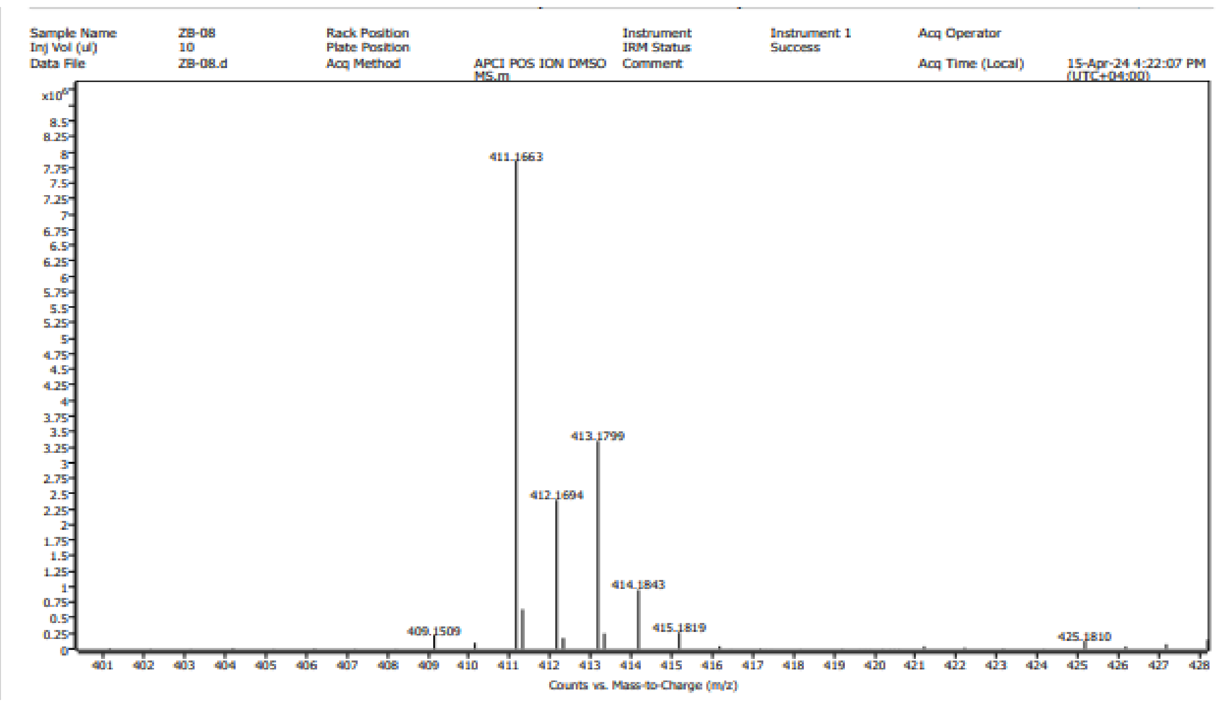

Mass spectrum of 5i

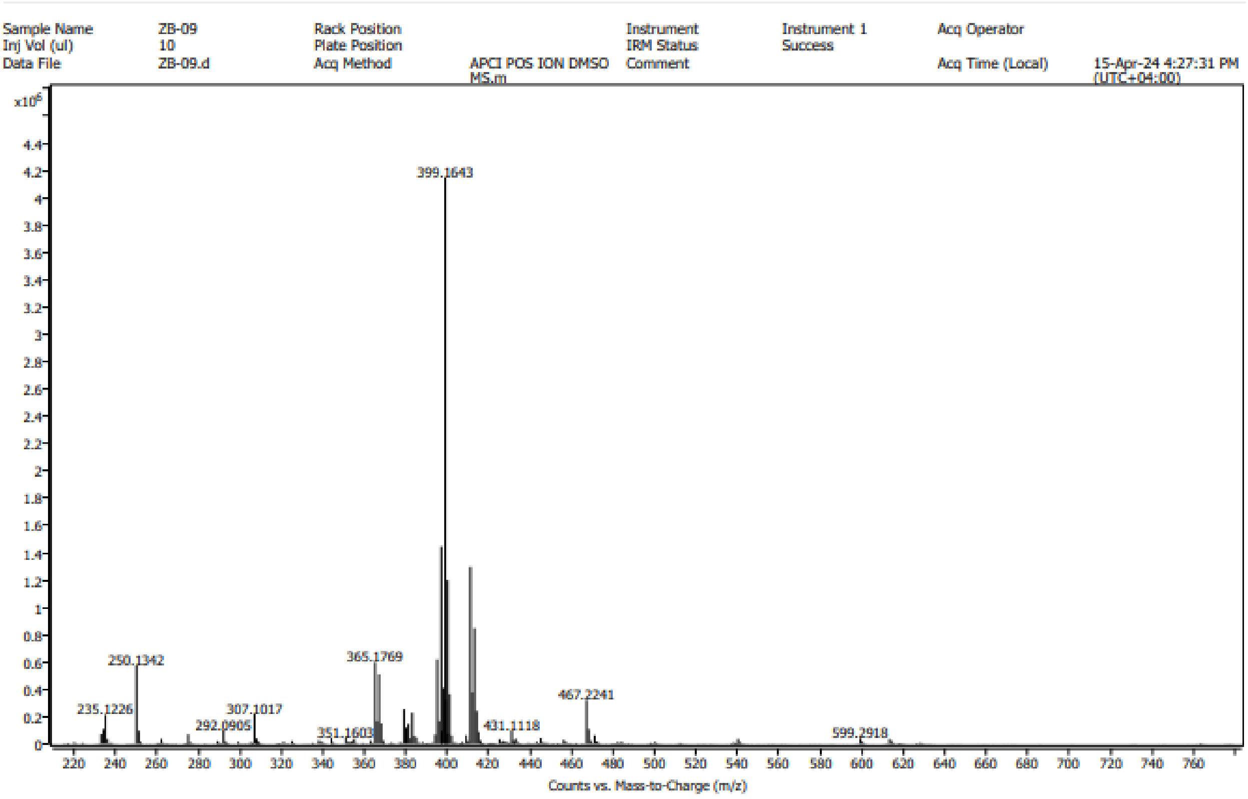

Mass spectrum of 5j

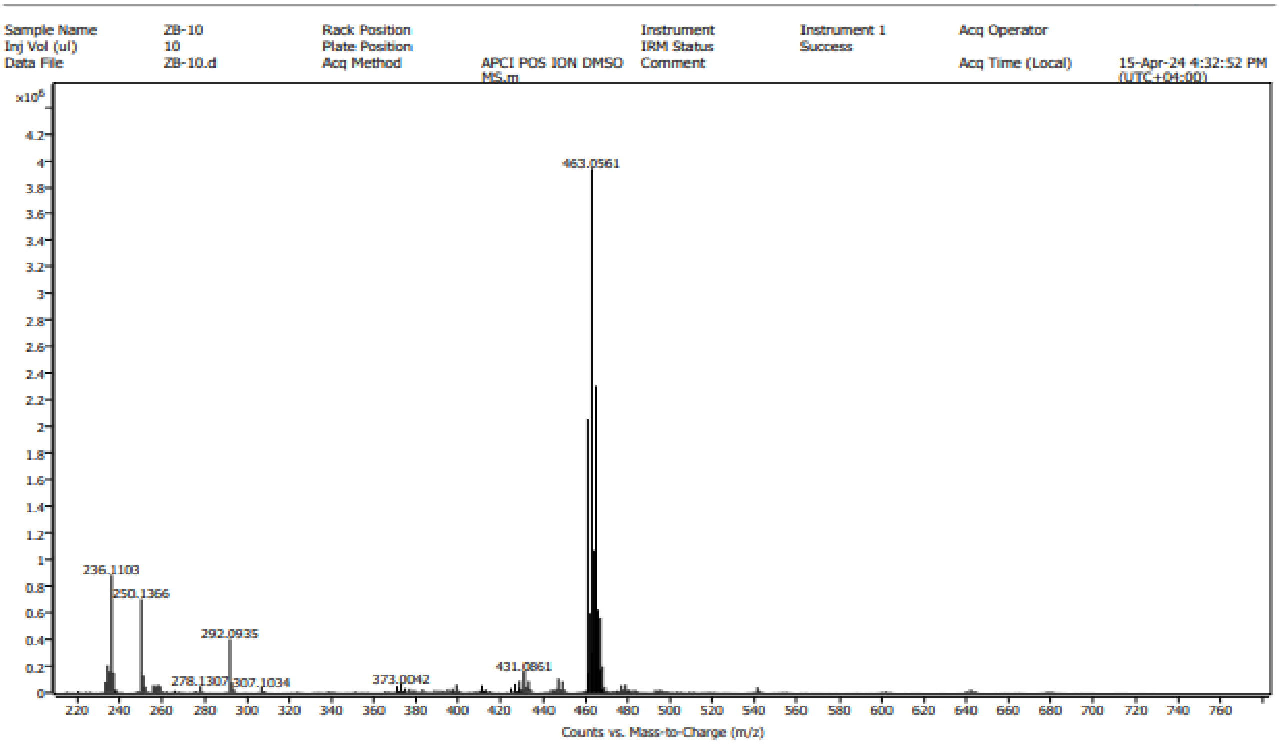

Mass spectrum of 5k

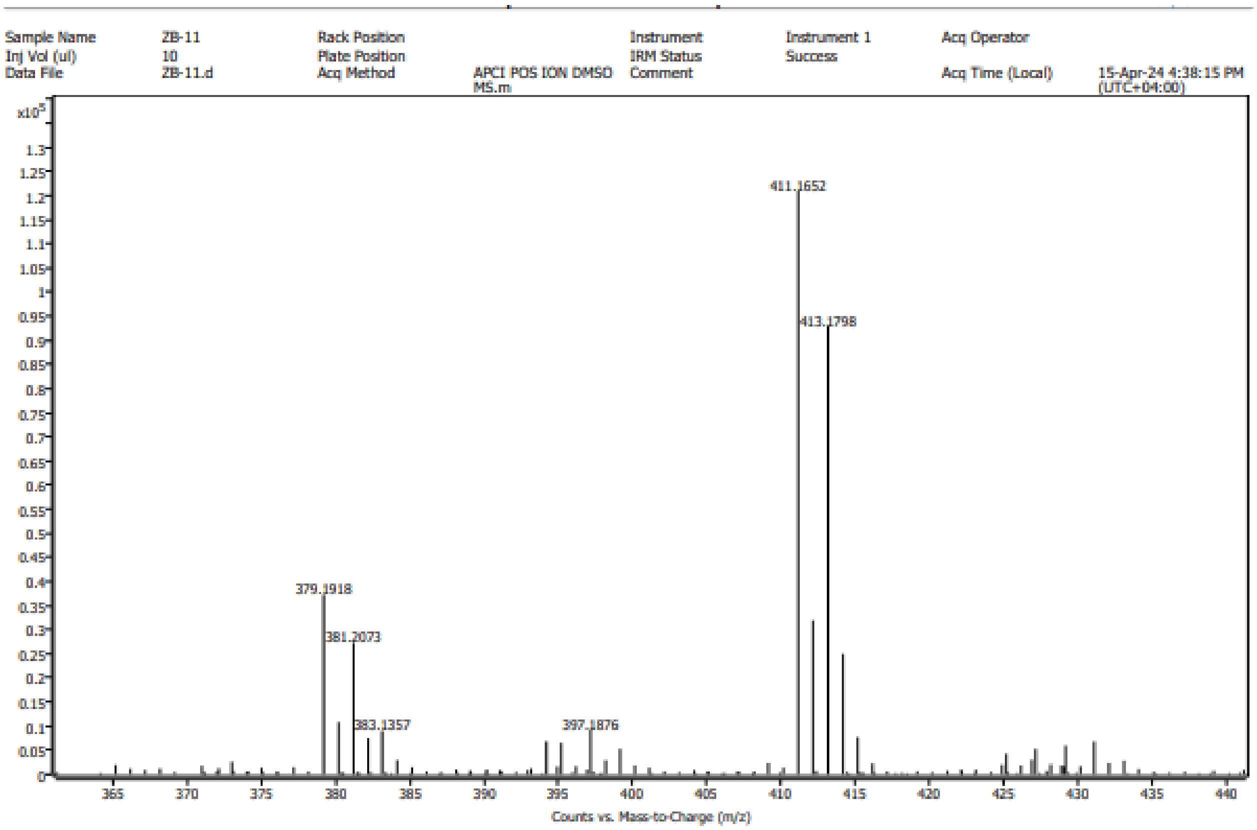

Mass spectrum of 5l

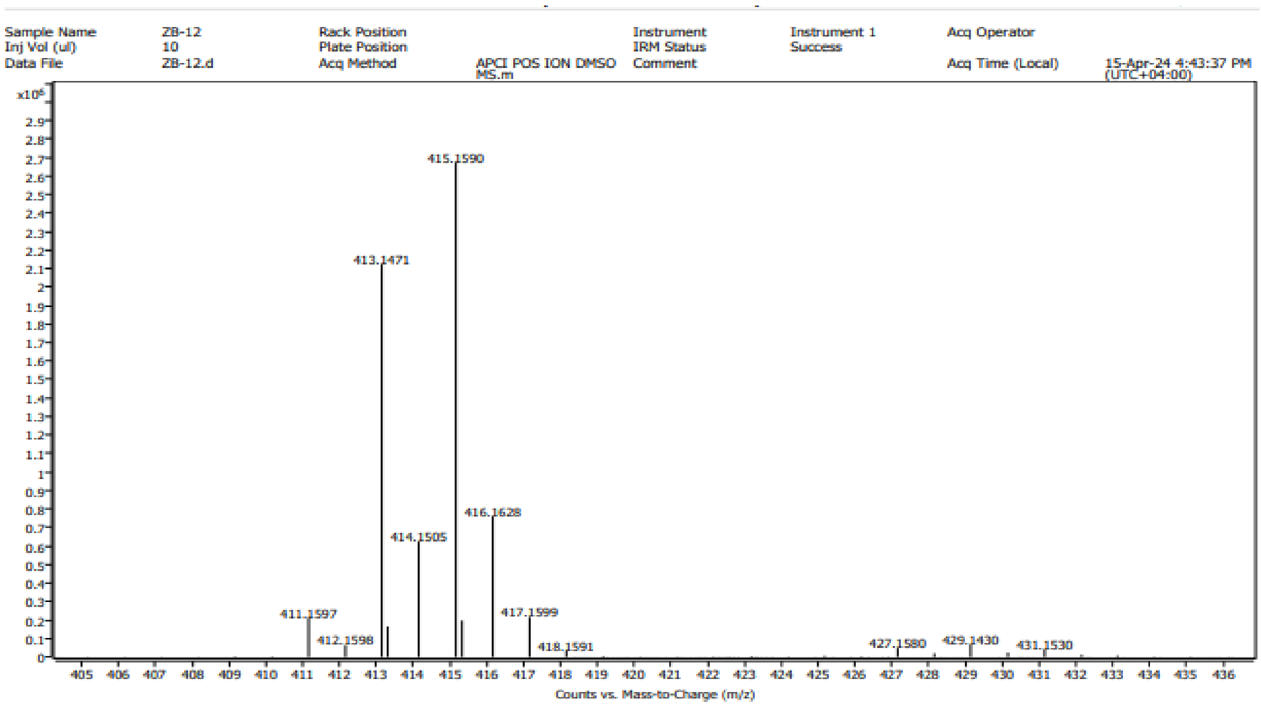

Mass spectrum of 5m

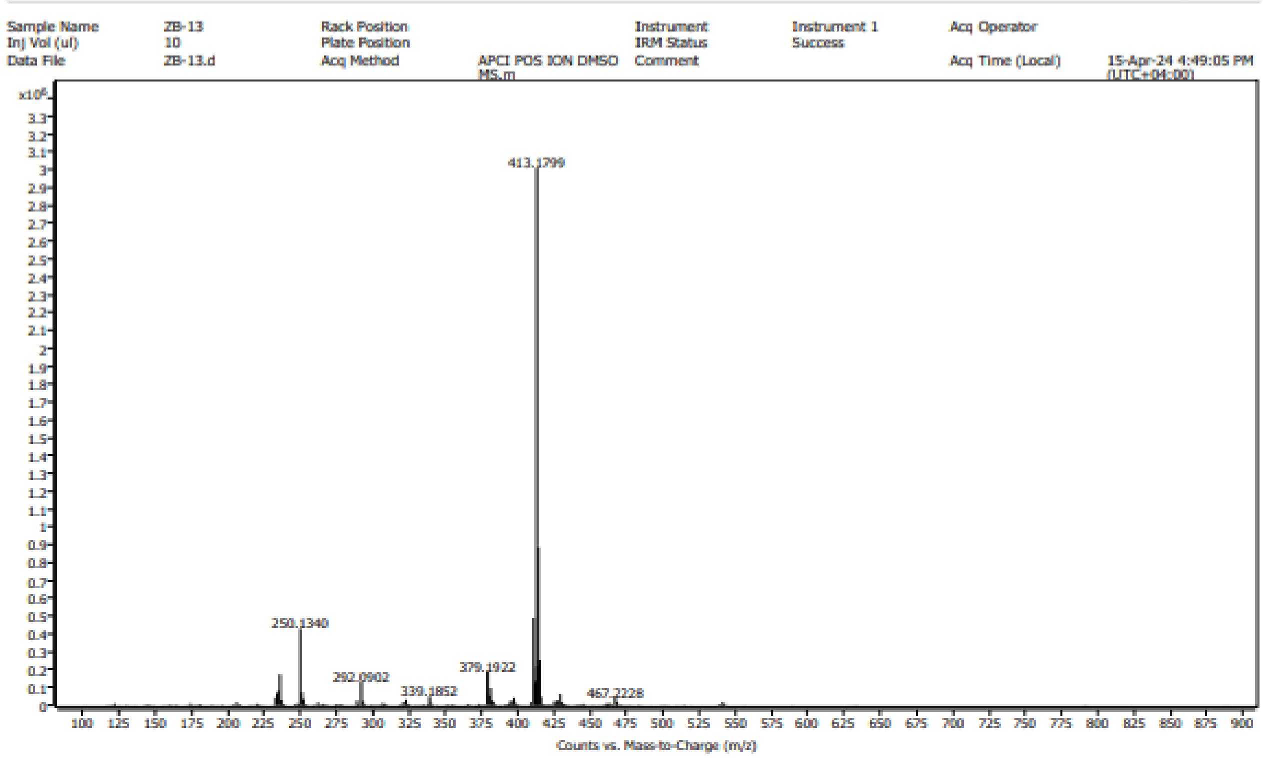

Mass spectrum of 5n

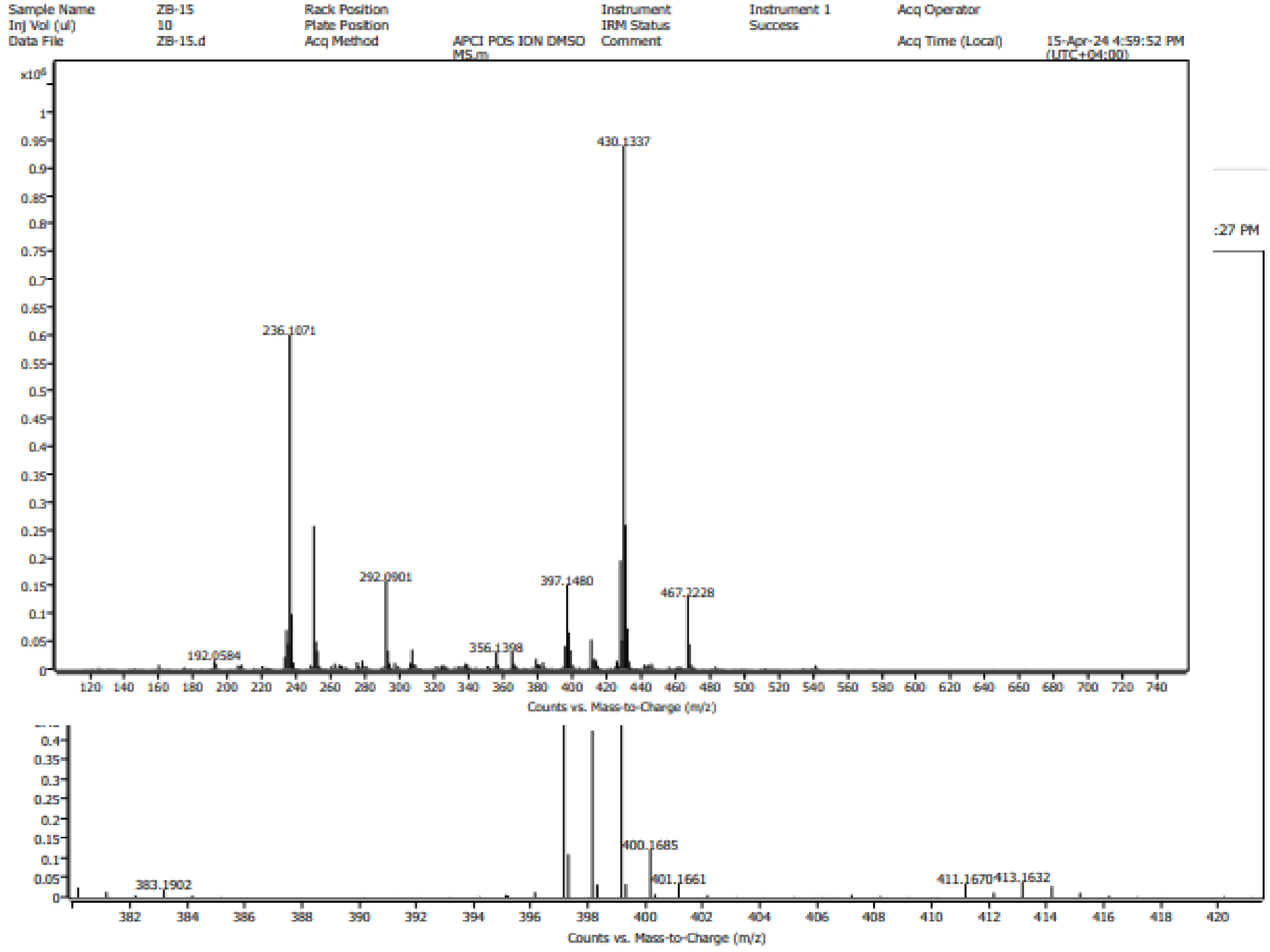

Mass spectrum of 5o

Mass spectrum of 5p

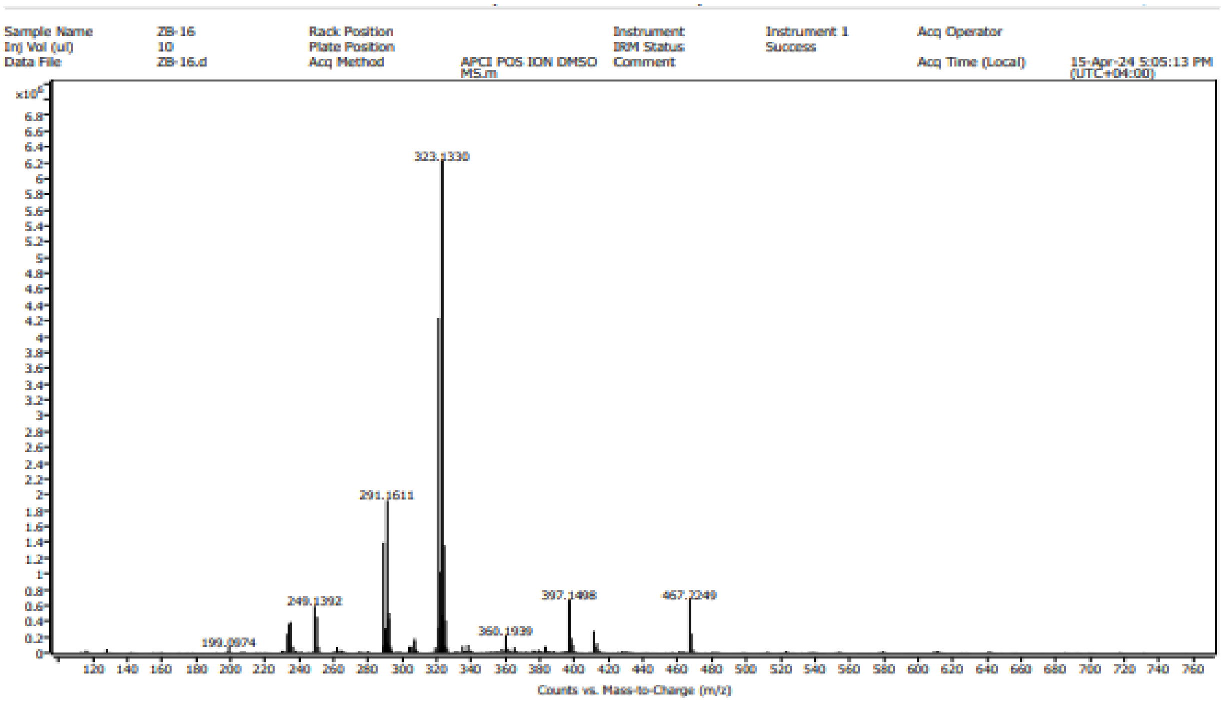

Mass spectrum of 5q

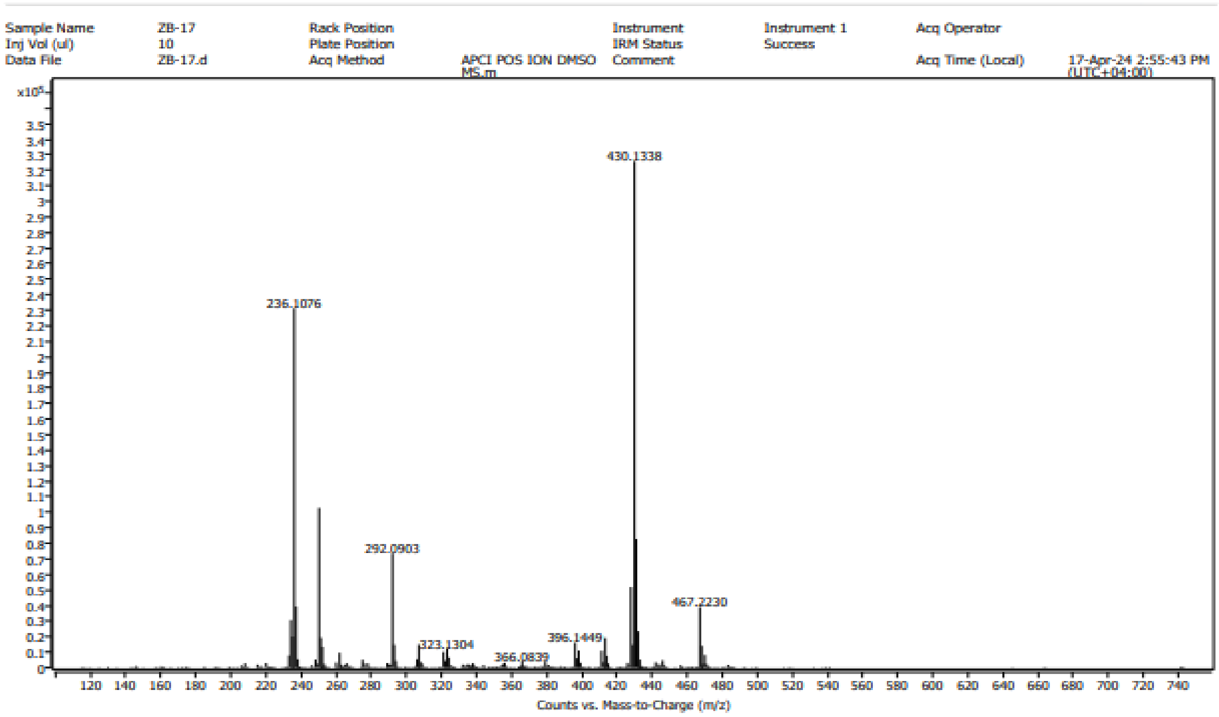

Mass spectrum of 5r

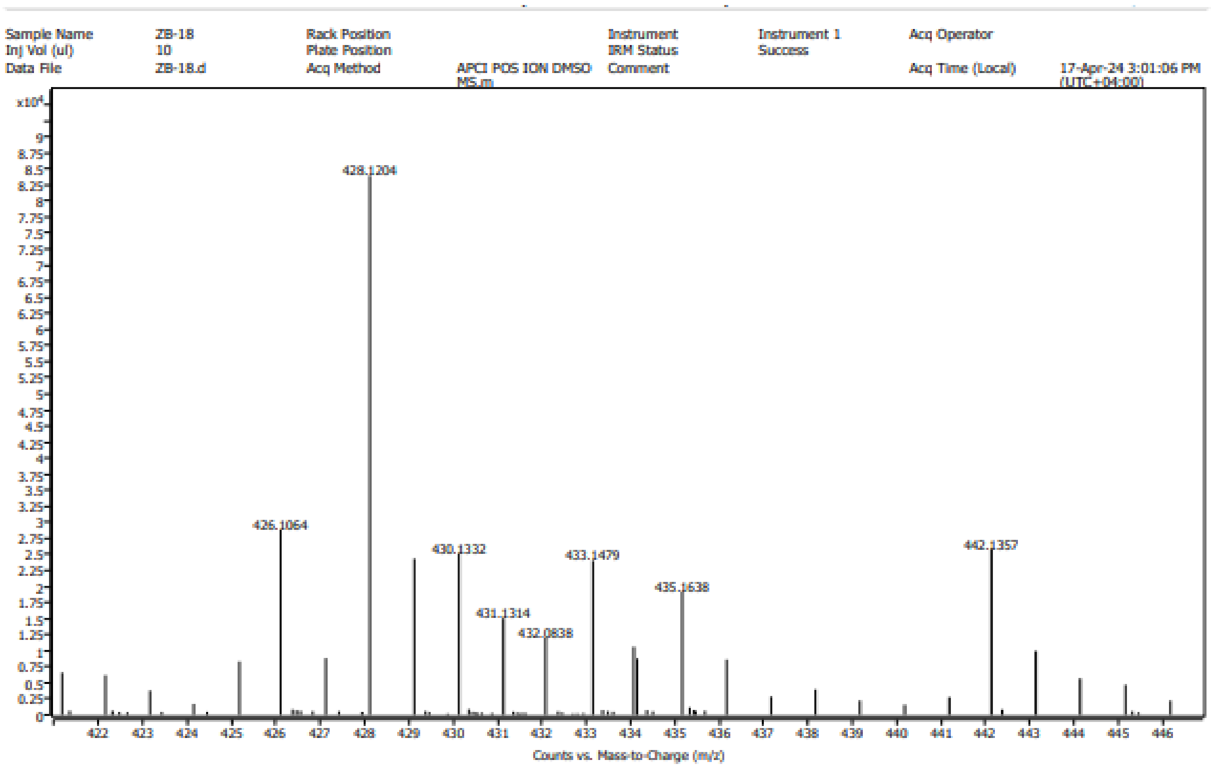

Supplement: RA-014-D4RA05015K-s002 [file RA-014-D4RA05015K-s002.pdf]
